# Effect of Smartphone App–Based Education on Clinician Prescribing Habits in a Learning Health Care System: A Randomized Cluster Crossover Trial

**DOI:** 10.1001/jamanetworkopen.2022.23099

**Published:** 2022-07-26

**Authors:** Matthew D. McEvoy, Mary Lynn Dear, Reagan Buie, David A. Edwards, Tyler W. Barrett, Brian Allen, Amy C. Robertson, Leslie C. Fowler, Cassandra Hennessy, Bonnie M. Miller, Kim V. Garvey, Robert P. Bland, Geoffrey M. Fleming, Don Moore, Todd W. Rice, Gordon R. Bernard, Christopher J. Lindsell

**Affiliations:** 1 Department of Anesthesiology, Vanderbilt University Medical Center, Nashville, Tennessee; 2 Vanderbilt Institute for Clinical and Translational Research, Vanderbilt University Medical Center, Nashville, Tennessee; 3 Episodes of Care, Vanderbilt University Medical Center, Nashville, Tennessee; 4 Department of Emergency Medicine, Vanderbilt University Medical Center, Nashville, Tennessee; 5 Department of Biostatistics, Vanderbilt University Medical Center, Nashville, Tennessee; 6 Department of the Office of Health Sciences Education, Vanderbilt University Medical Center, Nashville, Tennessee; 7 Department of HealthIT Architecture and Integration, Vanderbilt University Medical Center, Nashville, Tennessee; 8 Department of Pediatrics, Vanderbilt Children’s Hospital, Nashville, Tennessee; 9 Professor of Medical Education and Administration, Emeritus, Vanderbilt University School of Medicine, Nashville, Tennessee; 10 Division of Pulmonary, Allergy, and Critical Care Medicine, Department of Medicine, Vanderbilt University Medical Center, Nashville, Tennessee

## Abstract

**Importance:**

Effective methods for engaging clinicians in continuing education for learning-based practice improvement remain unknown.

**Objective:**

To determine whether a smartphone-based app using spaced education with retrieval practice is an effective method to increase evidence-based practice.

**Design, Setting, and Participants:**

A prospective, unblinded, single-center, crossover randomized clinical trial was conducted at a single academic medical center from January 6 to April 24, 2020. Vanderbilt University Medical Center clinicians prescribing intravenous fluids were invited to participate in this study.

**Interventions:**

All clinicians received two 4-week education modules: 1 on prescribing intravenous fluids and 1 on prescribing opioid and nonopioid medications (counterbalancing measure), over a 12-week period. The order of delivery was randomized 1:1 such that 1 group received the fluid management module first, followed by the pain management module after a 4-week break, and the other group received the pain management module first, followed by the fluid management module after a 4-week break.

**Main Outcomes and Measures:**

The primary outcome was evidence-based clinician prescribing behavior concerning intravenous fluids in the inpatient setting and pain medication prescribing on discharge from the hospital.

**Results:**

A total of 354 participants were enrolled and randomized, with 177 in group 1 (fluid then pain management education) and 177 in group 2 (pain management then fluid education). During the overall study period, 16 868 questions were sent to 349 learners, with 11 783 (70.0%) being opened: 10 885 (92.4%) of those opened were answered and 7175 (65.9%) of those answered were answered correctly. The differences between groups changed significantly over time, indicated by the significant interaction between educational intervention and time (*P* = .002). Briefly, at baseline evidence-concordant IV fluid ordered 7.2% less frequently in group 1 than group 2 (95% CI, −19.2% to 4.9%). This was reversed after training at 4% higher (95% CI, −8.2% to 16.0%) in group 1 than group 2, a more than doubling in the odds of evidence-concordant ordering (OR, 2.56, 95% CI, 0.80-8.21). Postintervention, all gains had been reversed with less frequent ordering in group 1 than group 2 (−9.5%, 95% CI, −21.6% to 2.7%). There was no measurable change in opioid prescribing behaviors at any time point.

**Conclusions and Relevance:**

In this randomized clinical trial, use of smartphone app learning modules resulted in statistically significant short-term improvement in some prescribing behaviors. However, this effect was not sustained over the long-term. Additional research is needed to understand how to sustain improvements in care delivery as a result of continuous professional development at the institutional level.

**Trial Registration:**

ClinicalTrials.gov Identifier: NCT03771482

## Key Points

**Question:**

Can delivery of educational content via a smartphone-based app using spaced education and retrieval practice change patient care practices?

**Findings:**

In this randomized clinical trial comprising 354 clinicians, use of spaced education with retrieval practice delivered via a webapp was associated with a statistically significant doubling of the odds of fluid prescribing behaviors being consistent with evidence-based standards. However, the improvement was not sustained after 8 to 12 weeks.

**Meaning:**

The findings of this trial show that disseminating learning at scale to frontline clinicians is possible through spaced education via an app.

## Teaser

This randomized clinical trial examines the effect on clinician prescribing habits of a smartphone-based app that uses spaced education with retrieval practice to educate clinicians on evidence-based prescribing of balanced crystalloids vs saline and opioid vs nonopioid medications.

## Introduction

Timely implementation of new evidence remains suboptimal in health care.^1^ Effective approaches for rapid dissemination and education on the latest evidence may help bridge this implementation gap.^2,3^ However, the best method for engaging clinicians in ongoing education for practice-based learning and improvement remains unknown.^4,5,6,7,8,9,10,11^ Research has demonstrated that passive learning is insufficient to produce practice change,^12^ yet most continuing education offerings in health care rely on passive learning models through lecture-style presentations.

Spaced education and retrieval practice with feedback have been reported to be effective methods of education.^13,14,15^ Spaced education distributes learning over multiple shorter encounters.^16^ Retrieval practice actively engages the learner by querying their knowledge base and then giving feedback about their responses.^11^ The combination of spaced education and retrieval practice can be better than traditional pedagogical approaches for clinicians, with evidence suggesting improved knowledge acquisition, knowledge retention, and, in some cases, clinical practice.^17,18,19,20,21,22,23,24,25,26,27,28^ The combination of spaced education and retrieval practice also has the potential for deployment at the institutional level for large learner groups. Whether spaced education with retrieval practice can change prescribing patterns at the institutional level is unknown.

To disseminate learning at scale using spaced education with retrieval practice, Vanderbilt University School of Medicine created QuizTime, a web-based smartphone app (eAppendix 2 in Supplement 2).^3^ This study evaluated the effect of using QuizTime on clinician prescribing patterns for intravenous fluids and for pain medications. Fluid prescribing patterns were selected because a large pragmatic clinical trial performed at our institution demonstrated a 0.9% mortality benefit of balanced crystalloids (lactated Ringer’s solution or Plasma-Lyte A [Normosol]) compared with saline (0.9% NaCl) when used as the routine fluid choice in the intensive care unit environment.^29^ To avoid threats to internal validity when a learning intervention is tested only against no intervention, a counterbalancing condition was warranted.^30^ We selected pain management prescribing practices for this purpose based on an institutional priority promoting evidence-based use of pain management strategies with avoidance of opioid monotherapy for pain management.^31^

We hypothesized that, following the educational intervention on intravenous fluid management, there will be an increase in the proportion of balanced crystalloids ordered compared with saline, consistent with published results.^29,32^ Similarly, we hypothesized a decrease in prescribing of opioid monotherapy at hospital discharge following delivery of that education content.

## Methods

### Trial Design and Study Population

**Figure 1.**
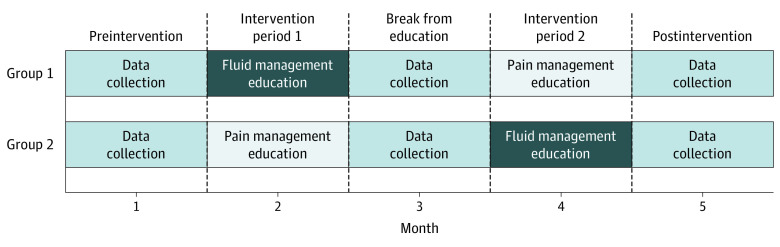
Study Design Group 1 received the fluid management module followed by the pain management module. Group 2 received the pain management module followed by the fluid management module.

This single-center, pragmatic, randomized cluster crossover clinical trial was designed to test the efficacy of QuizTime in influencing prescribing behavior (protocol presented in Supplement 1). Briefly, the study delivered 1 educational module on prescribing intravenous fluids and 1 on prescribing opioid and nonopioid medications to enrolled clinicians over a 12-week period (Figure 1). Clinicians were randomized to receive fluids education first followed by opioid prescribing or to receive opioid prescribing education first followed by fluids education. The first education module was deployed to all clinicians simultaneously, then the clusters were crossed over at the same time to the second educational module. The trial was considered exempt from continuing review by the local institutional review board. This study followed the Consolidated Standards of Reporting Trials (CONSORT) reporting guideline.

### Recruitment, Randomization, and Educational Intervention

After institutional review board approval by the Vanderbilt University Human Research Protections Program, an invitation to participate in training was extended to all prescribing clinicians (faculty physicians, residents, fellows, and advanced practice clinicians) at Vanderbilt University Medical Center via email with an embedded sign-up link (eAppendix 1 in Supplement 2). Enrollment in the study through the online REDCap system was voluntary. As the clinical data evaluated in this study were existing data, the institutional review board granted the study exempt status. Participants had 3 weeks to enroll and were offered the American Medical Association Physician's Recognition Award category 1 credit (hereafter, credit) for completing each module. Module content, based on existing evidence, was developed by a team of physicians and advanced practice clinicians (M.D.M., D.A.E., T.W.B., B.A., A.C.R., L.C.F., K.V.G., G.M.F., T.W.R., and G.R.B.) using a previously described 4-step model.^29,31,32,33,34,35,36,37,38^

Participants were randomized 1:1 to either the fluid management module followed by the pain management module (group 1) or the pain management module followed by the fluid management module (group 2). After completing the first module, a 4-week period with no question delivery was implemented before starting the second module. Clinicians who initially received the pain management module received the fluid management module, and those who had received the fluid management module then received the pain management module.

Each module delivered 1 multiple choice question per day on weekdays (ie, Monday through Friday) to participants over a 4-week period with 20 question items per module. Clinicians were able to self-select question receipt by email or text message. Participants were given 24 hours to answer a question after delivery. The question display showed participants a question stem and 4 possible answers. Participants could select an answer by choosing 1 of the unnumbered and unlabeled radio buttons.

On submission of a correct answer, participants received a green background version of the explanation that included the question’s key point, rationale, and references. Participants read through and acknowledged the green background version of the explanation to receive credit for completing the question item and to be eligible for credit. If the participant opened the question and answered it incorrectly, they received the same feedback with key point, rationale, and references as if answered correctly, but with a red background displayed. If participants read through this feedback material and acknowledged having done so, they were given an opportunity to reattempt answering the question and obtain credit. Participants were given 24 hours to submit a question reattempt. On resubmission of a correct answer, participants were sent the green background version of the explanation. If the answer submitted on the second attempt was again incorrect, the red background explanation was displayed again, no credit was given, and no further opportunity to answer the question was provided (eFigure in Supplement 2). Participants were eligible for 0.25 credits per question answered correctly and reviewed.

No other fluid or pain medication prescribing intervention was implemented during the study period. Importing the educational content (eg, question stems, answers, rationales) into the QuizTime system, management of the enrollment process, and management of feedback/queries from participants required approximately 0.2 full-time equivalents of administrative support for 3 weeks before the study and 0.1 full-time equivalent throughout the duration of the study period.

### Outcomes

The primary study outcome was prescribing behavior. Specifically, we evaluated the percentage of all inpatient orders for balanced intravenous fluid solutions (ie, not saline) as well as the median morphine milligram (MME) equivalents per opioid prescription. The initial statistical plan was for the primary outcome to be the proportion of patient visits attended that resulted in an opioid prescription and secondarily the average MME prescribed. We switched the median MME per order to primary because we realized that there were insufficient orders per individual clinician for proportion of orders to be meaningful (eg, if there are only 5 orders, possible outcomes are 0, 20, 40, 60, 80, or 100. If there are 2 orders, possible outcomes are 0, 50, or 100). This was updated to clinicaltrials.gov and reported on the study record. These metrics were estimated for each participant for the 4-week period preceding training, the 4-week interval between training periods, and the 4-week period after completion of the second training period. A fluid order was considered evidence-concordant if it was for lactated Ringer’s solution or Plasma-Lyte A (Normosol), but not if it was saline. Other fluid orders (eg, albumin, blood products) were not scored. As a secondary outcome, we analyzed the percentage of discharge orders that included the scheduled use of a nonopioid analgesic medication.

A set of discharge orders for analgesics was considered evidence-concordant if any nonopioid analgesic was prescribed with or without an opioid prescription, but not if only opioids were prescribed. This scoring of clinician behavior was consistent with the material presented in the education modules for fluids and analgesics but was not stated in the educational content.

### Data Collection

Data on prescribing practices were collected as part of routine clinical care and extracted from electronic health records. Participant characteristics were self-reported and collected during enrollment. In addition to the electronic health record data extract, the Vanderbilt Committee on Opioid Monitoring and Stewardship systematically curates opioid prescribing data, including clinician details, prescribing location, prescription details for opioid and nonopioid pain medications, and morphine milligram equivalent conversions of daily opioid prescriptions. The Vanderbilt Committee on Opioid Monitoring and Stewardship database was used for opioid prescribing. Learner analytics for the educational modules were extracted from the QuizTime database and included number of questions delivered, opened, and answered, as well as sent and attempted a second time. The proportion of questions that were answered correctly and the number of second attempts that were opened and correctly answered were collected.

### Statistical Analysis

Descriptive statistics were used to summarize participant characteristics and question response rates and accuracy by trial group. To explore the influence of training on clinician ordering behavior, groups were compared using a linear regression model. Subsequently, a mixed-effects logistic regression model was constructed with individual order status (consistent or not consistent with evidence) as the outcome and with clinician modeled as a random effect. Separate models were constructed for fluid-ordering and opioid-ordering behavior. Group assignment and the period (baseline, after first training, and after second training) were used as estimators in the model. An interaction between period and group assignment was used to evaluate whether differences over time were affected by group membership; an overall effect of the group by period interaction is presented. For models exploring the fluid-ordering behavior, group 1 (fluid management first) is the intervention group and group 2 (pain management first) is the control group. This sequence was reversed for opioid-ordering behavior. In all models, we hypothesized (1) there would be no statistically significant difference between groups at baseline, (2) after the first training period there would be improved ordering practices in the intervention group compared with the control group and compared with baseline, and (3) after the second training period there would be no statistically significant difference between groups, but both the intervention and control group experience would show improved ordering practices compared with baseline.

Clinicians who ordered at least 1 fluid were included in the main analysis exploring the effectiveness of fluid management training. Those who prescribed at least 1 opioid were included in the main analysis exploring the effectiveness of opioid training. A sensitivity analysis was conducted using only clinicians with at least 5 orders for the given outcome.

Analyses were performed using R, version 4.0.2 (R Foundation for Statistical Computing), including the lme4 package. The significance threshold was 2-sided and unpaired with a *P* = 05.

## Results

**Figure 2.**
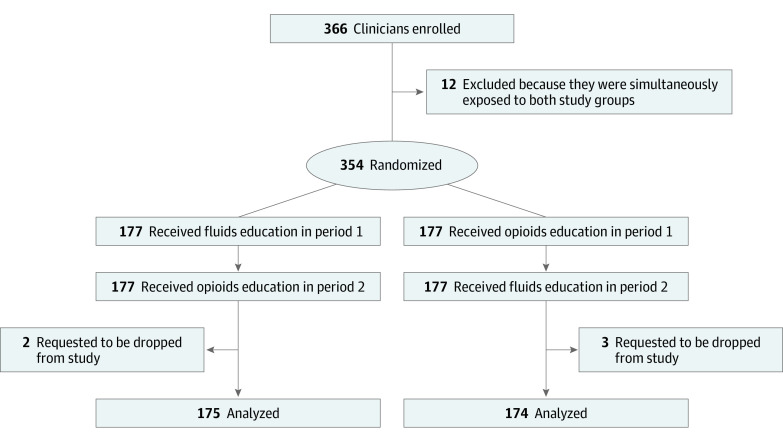
Participant Flow Diagram All clinicians were analyzed by their treatment assignment.

A total of 354 clinicians were enrolled and randomized, with 177 in group 1 (intervention: fluid followed by opioid education) and 177 in group 2 (control: opioid followed by fluid education). Five individuals requested to be withdrawn from the study owing to not wanting to receive the daily questions (2 in group 1 and 3 in group 2). Therefore, final analysis included 175 participants in group 1 and 174 participants in group 2 (Figure 2).

Table 1 describes the characteristics of the participants, which included 140 physician and 209 nonphysician prescribers. During the overall study period (January 6 to April 24, 2020), 16 868 questions were sent to the 349 learners. Of those questions 11 783 (70.0%) were opened, 10 885 of 11 783 were answered (92.4% of opened), and 7175 of 10 885 were answered correctly (65.9% of those opened and answered). Learner analytics by group and by educational module are presented in the eTable in Supplement 2.

**Table 1.** Clinician Characteristics

Characteristic	No. (%)		
	Overall (N = 349)	Group 1	Group 2
		Fluid management first (n = 175)	Pain management first (n = 174)
Profession			
Physician (faculty/resident/fellow)	140 (40.1)	66 (37.7)	74 (42.5)
Physician assistant	14 (4.0)	8 (4.6)	6 (3.4)
Nurse practitioner	152 (43.6)	78 (44.6)	74 (42.5)
CRNA	33 (9.5)	17 (9.7)	16 (9.2)
Pharmacist	1 (0.3)	1 (0.6)	0
Other	9 (2.6)	5 (2.9)	4 (2.3)
Degree			
APRN	91 (26.1)	47 (26.9)	44 (25.3)
CNM	3 (0.9)	0	3 (1.7)
CRNA	29 (8.3)	16 (9.1)	13 (7.5)
DNP	27 (27.7)	14 (8.0)	13 (7.5)
DO	3 (0.9)	2 (1.1)	1 (0.6)
FNP	11 (3.2)	7 (4.0)	4 (2.3)
MD	138 (39.5)	64 (36.6)	74 (42.5)
NP	26 (7.4)	12 (6.9)	14 (8.0)
PA	14 (4.0)	8 (4.6)	6 (3.4)
Other	7 (2.0)	5 (2.9)	2 (1.1)
Specialties			
Anesthesiology	59 (16.9)	31 (17.7)	28 (16.1)
Cardiac surgery	10 (2.9)	6 (3.4)	4 (2.3)
Emergency medicine	13 (3.7)	5 (2.9)	8 (4.6)
Family medicine	13 (3.7)	8 (4.6)	5 (2.9)
Medicine	88 (25.2)	41 (23.4)	47 (27.0)
Neurologic surgery	4 (1.1)	1 (0.6)	3 (1.7)
Neurology	4 (1.1)	3 (1.7)	1 (0.6)
Obstetrics and gynecology	8 (2.3)	2 (1.1)	6 (3.4)
Ophthalmology and visual sciences	1 (0.3)	1 (0.6)	0
Oral maxillofacial surgery	3 (0.9)	2 (1.1)	1 (0.6)
Orthopedic surgery and rehabilitation	4 (1.1)	2 (1.1)	2 (1.1)
Otolaryngology	4 (1.1)	2 (1.1)	2 (1.1)
Pathology, microbiology, and immunology	2 (0.6)	0	2 (1.1)
Pediatric surgery	6 (1.7)	4 (2.3)	2 (1.1)
Pediatrics	73 (20.9)	35 (20.0)	38 (21.8)
Physical medicine and rehabilitation	2 (0.6)	0	2 (1.1)
Psychiatry	8 (2.3)	6 (3.4)	2 (1.1)
Radiation oncology	2 (0.6)	1 (0.6)	1 (0.6)
Radiology and radiologic sciences	7 (2.0)	4 (2.3)	3 (1.7)
Surgery	31 (8.9)	15 (8.6)	16 (9.2)
Thoracic surgery	1 (0.3)	1 (0.6)	0
Urologic surgery	3 (0.9)	3 (1.7)	0
Other	3 (0.9)	2 (1.1)	1 (0.6)
Top 3 primary areas of practice			
Ambulatory	38 (10.9)	19 (10.9)	19 (20.9)
Emergency department	42 (12.0)	21 (12.0)	21 (12.1)
Perioperative area (preoperative, OR, PACU)	92 (26.4)	45 (25.7)	47 (27.0)
Procedural area (GI suite, radiology)	39 (11.2)	20 (11.4)	19 (10.9)
ICU (all except neurocare)	132 (37.8)	63 (36.0)	69 (39.7)
Neurocare ICU	20 (5.7)	12 (6.9)	8 (4.6)
Medical unit/floor (non-ICU)	116 (33.2)	54 (30.9)	62 (35.6)
Surgical unit/floor (non-ICU)	55 (15.8)	29 (16.6)	26 (14.9)
Other	25 (7.2)	18 (10.3)	7 (4.0)
Clinician orders: fluids			
At least 1 order, No.	206	100	106
Orders per clinician, mean (SD)	39 (45)	37 (45)	42 (46)
Orders per clinician, median (IQR)	22 (8-57)	21 (8-51)	25 (9-62)
Clinician orders: discharge			
At least 1 order	140	70	70
Orders per clinician, mean (SD)	16 (23)	15 (23)	16 (24)
Orders per clinician, median (IQR)	7 (2-20)	8 (2-20)	7 (3-19)

Abbreviations: APRN, advanced practice registered nurse; CNM, certified nurse midwife; CRNA, certified registered nurse anesthetist; CRNP, certified registered nurse practitioner; DNP, doctor of nursing practice; DO, doctor of osteopathic medicine; FNP, family nurse practitioner; GI, gastrointestinal; ICU, intensive care unit; MD, doctor of medicine; NP, nurse practitioner; OR, operating room; PA, physician’s assistant; PACU, postanesthesia care unit.

### Fluid Prescribing

There were 206 participants who prescribed fluids at least once and were thus included in the fluid management analysis. At baseline, the control group ordered balanced crystalloids in 65.9% of the orders (averaged over clinician), and only 57.7% of orders were for balanced crystalloids in the intervention group (Table 2). At baseline, the intervention group demonstrated a 7.2% lower rate of evidence-concordant ordering intravenous fluids consistent with evidence than the control group (95% CI, −19.2% to 4.9%). After training, the rate of evidence-concordant ordering was 3.9% higher (95% CI, −8.2% to 16.0%). This improvement was reversed after intervention (−9.5%; 95% CI, −21.6% to 2.7%). The interaction between intervention and time was significant (*P* = .002). Similarly, at baseline the odds ratio (OR) for ordering balanced crystalloids was 0.53 (95% CI, 0.17-1.71) for those who received fluid management training first (intervention group, 70.7% of orders were for balanced crystalloids at baseline) compared with those who received pain management training first (control group, 66.8% of orders were for balanced crystalloids at baseline). Immediately after the initial training, the control group ordered balanced crystalloids in 61.0% of the orders and the intervention group ordered balanced crystalloids in 78.6% of the orders (OR, 2.56; 95% CI, 0.80-8.21). Four weeks later, after both groups had received fluid management training, the control group ordered balanced crystalloids 70.8% of the time and the intervention group had reverted to 67.6% orders for balanced crystalloids (OR, 0.44; 95% CI, 0.14-1.43). The interaction between intervention and time was significant (*P* < .001), showing a positive effect of the training on prescribing behavior immediately and up to 4 weeks after training, but that the effect did not persist 8 to 12 weeks after training.

**Table 2.** Primary Outcome Results

Variable	No. (%)^a^	
	Group 1 (fluid management first)	Group 2 (pain management first)
**Proportion of evidence-concordant balanced crystalloids ordered**		
Preintervention	897 (70.7)	1011 (66.8)
After period 1	1112 (78.6)	960 (61.0)
After period 2	656 (67.6)	966 (70.8)
**Proportion of evidence-concordant discharge orders**		
Preintervention	385 (94.1)	413 (90.6)
After period 1	384 (92.3)	405 (93.1)
After period 2	211 (94.2)	235 (92.2)

^a^ Percentages come from the total number of orders in each cell (897/1268 = 70.7% of the total number of fluids orders in the preintervention time frame for clinicians in group 1 are balanced crystalloids).

Our sensitivity analysis including only clinicians with more than 5 fluid orders showed similar results. The ORs for guideline-adherent ordering were 0.53 (95% CI, 0.15-1.84) at baseline, 2.44 (95% CI, 0.70-8.48) immediately after the initial training, and 0.43 (95% CI, 0.12, 1.51) at training.

### Pain Management

There were 140 clinicians who placed at least 1 opioid order at discharge and were thus included in the analysis. There was no significant difference in the morphine milligram equivalents per prescription between groups (group by interaction *P* = .71; between group contrasts at each time period *P* > .05). Clinicians increased the average proportion of discharge opioids orders rated as evidence-concordant in the periods immediately following pain management training. At baseline, the intervention group prescribed 90.6% of discharge orders in an evidence-concordant manner compared with 94.1% of orders in the control group. The OR for writing a discharge order that was evidence-concordant was 0.68 (95% CI, 0.26-1.83) for those who received pain management training first compared with those who received fluid management training first. Following the first training period, the proportion of discharge orders written by the intervention group that were evidence-concordant slightly increased from baseline to 93.1%, while the proportion in the control group decreased to 92.3% (OR, 1.12; 95% CI, 0.42-3.04). After both groups received pain management training, the intervention group had 92.2% of discharge orders that were evidence-concordant according to the criteria and the control group, who had just completed the pain management training, increased the proportion of discharge orders that were evidence-concordant to 94.2% (OR, 0.85; 95% CI, 0.28-2.60). The interaction between intervention and time was not significant (*P* = .54).

Our sensitivity analysis including only clinicians with more than 5 fluid orders showed similar results, with the ORs for guideline-adherent ordering being 0.74 (95% CI, 0.28-1.99) at baseline, 1.21 (95% CI, 0.45-3.30) immediately after the initial training, and 0.85 (95% CI, 0.27-2.64) after final training.

## Discussion

An idealized learning health care system is designed to learn from what it does and then do what it learns, thereby closing the evidence-to-practice gap.^3,39^ Great strides have been made in knowledge generation, yet considerable challenges remain in the dissemination and uptake of that new knowledge into routine practice.^40^ We evaluated whether the provision of spaced education with retrieval practice to frontline clinicians could change prescribing practices. We found that engagement with QuizTime learning modules doubled the odds of clinicians ordering intravenous fluids in a manner consistent with published data for 4 weeks after the intervention and this finding was statistically significant. However, this effect was not sustained 8 to 12 weeks later. Similar effects were not observed for the balancing condition of opioid prescribing. We suspect this lack of a change was because opioid prescribing practices have been a focus of education at our institution and across the state of Tennessee, and thus there may have been less room for practice change given such ongoing attention.

The link between spaced education through multiple-choice questions and improvements in clinical performance is not new. Shaw et al^6^ demonstrated that primary care clinicians who received spaced education self-reported substantially more clinical practice change than those who received only standard didactics, and Barrett et al^28^ showed a change among emergency department clinicians who received training with QuizTime. Kerfoot et al^19^ reported that spaced education improved clinical behaviors related to prostate cancer screening in a trial of 95 primary care clinicians. Our study builds on these findings in 2 ways. First, we performed a much larger trial with an interprofessional group of participants from multiple different medical specialties. Second, we demonstrated that this educational intervention can be implemented at scale across our entire enterprise, including medical and surgical wards, perioperative areas (eg, operating room and recovery room), and the intensive care units. As such, this study provides evidence that this pedagogical approach is feasible and can result in evidence-based improvements in clinician ordering practices across a health system.

### Limitations

This study has limitations. Although we showed some change in clinician practice, this change might have been increased by incorporating feedback through real-time decision support in the electronic health records or through a dashboard summarizing clinician performance.^41,42,43,44^ Also, this study was not able to assess the clinical context in which individual decisions were being made; there are circumstances in which saline or opioid monotherapy would be appropriate, although our experience is that such relevance is present in approximately less than 5% of cases.

## Conclusions

Creating a sustained learning effect that results in continued practice improvement remains a challenge in continuing medical education; traditional didactics involving passive learning does not generally create sustained change.^12,45,46^ To our knowledge, only 1 study^19^ has reported a durable effect from an initial educational intervention on sustained practice change, with effect observed as far as a year out. Future trials should assess the optimal dose (number of question items), timing (initial and follow-up booster training), and duration of education to promote sustained practice change. In addition, research is needed to determine how educational interventions should be paired with other implementation strategies, such as performance feedback, appropriate incentives, and removal of barriers for performing evidence-based care. Implementation strategies ranging from real-time best-practice advisories during electronic ordering, easy updating of order sets to reflect best practice, and personalized feedback in relation to unit and hospital goals for specific process metrics might all contribute to effective practice change.

In this study, we performed a large, pragmatic crossover randomized clinical trial and demonstrated that learning at scale among frontline clinicians is possible when using an innovative technological approach based on modern learning theory. This pedagogical approach may lead to significant, albeit transient, improvements in prescriber behavior.

## Notes

Supplement 1.
Trial Protocol and Statistical Analysis Plan

Supplement 2. **eAppendix 1.** Communication Announcement **eAppendix 2.** QuizTime Module **eFigure.** Example of QuizTime User Interface **eTable.** Learner Analytics

Supplement 3. **Nonauthor Collaborators.** The Vanderbilt Learning Healthcare System Platform Investigators and the Vanderbilt Committee on Opioid Monitoring and Stewardship

Supplement 4.
Data Sharing Statement
