# Supplementary material for: Effect of Smartphone App–Based Education on Clinician Prescribing Habits in a Learning Health Care System: A Randomized Cluster Crossover Trial
Source: JAMA Netw Open. 2022 Jul 26;5(7):e2223099. doi: 10.1001/jamanetworkopen.2022.23099 (PMC9327570; doi:10.1001/jamanetworkopen.2022.23099)
Supplement: Supplement 1. — Trial Protocol and Statistical Analysis Plan [file jamanetwopen-e2223099-s001.pdf]

Study Type and Performance Site Information

Type of study:

[ ] Standard or Expedited
[x] **Exempt**
[ ] Umbrella Review for funds release
[ ] Comparative Effectiveness Research
[ ] Non-Human Subject Determination
[ ] Quality Improvement/Non-Research Determination
[ ] Request review by another IRB
[ ] Coordinating Center ONLY

Please indicate which Committee is most appropriate to review your project:

[ ] Social and Behavioral Sciences
[x] **Health Sciences**

Are there any international sites involved in this study in which the PI is responsible?

[ ] Yes
[x] **No**

Is this project cancer-related?

[ ] Yes
[x] **No**

Study Purpose and Description

Provide a brief abstract of the study in lay language. The IRB Committees are comprised of scientists with varied backgrounds, non-scientists, and community members.

This study aims to evaluate the impact of spaced education, delivered via a smartphone application, on provider prescribing patterns.

Does your study fit into one or more of the listed categories of exemption (45 CFR 46.104)?

[ ] (d)(1) Research involving normal educational practices that are not likely to adversely impact students' opportunity to learn required educational content or the assessment of educators who provide instruction such as: (1) Most research on regular and special education instructional strategies; or (2) Research on the effectiveness of or the comparison among instructional techniques, curricula, or classroom management.
[**x**] **(d)(2) Research that only includes interaction involving the use of educational tests (cognitive, diagnostic, aptitude, achievement), survey procedures, interview procedures or observation of public behavior (including visual or auditory recording) if at least one of the following is met: (1) Information obtained is recorded by the investigator in such a manner that the identity of human subjects cannot be readily ascertained; or (2) Any disclosure of the human subjects' responses outside of the research would not reasonably place the subjects at risk or; (3) The information obtained is recorded by the investigator in a manner that could identify the human subjects directly or through identifiers linked to the subjects.**
[ ] (d)(3): Research involving benign behavioral interventions in conjunction with the collection of information from an adult subject through verbal written responses (including data entry) or audiovisual recording if the subject prospectively agrees to the intervention and information collected and at least one of the following is met; (1) The information obtained is recorded by the investigator in such a manner that the identity of the human subjects cannot be readily ascertained; (2) Any disclosure of the human subjects' responses outside the research would not reasonably place the subjects at risk; or (3) The information obtained is recorded in a manner that could identify the human subjects directly or through identifiers linked to the subjects. Note: Children may not be included in research under this exemption.
[ ] (d)(4): Secondary research for which consent is not required: Secondary research uses of identifiable private information or identifiable biospecimens, if at least one of the following criteria are met: (1) The identifiable private information or biospecimens are publically available; or (2) Information, which may include information about biospecimens, is recorded by the investigator in such a manner that the identity of the human subjects cannot readily be ascertained, the investigator does not contact the subjects, and the investigator will not re-identify subjects; or (3) The research involves only information, collection, and analysis involving the investigator's use of identifiable health information when that use is regulated under HIPAA for the purposes of "health care operations" or "research", or for "public health activities and purposes" under HIPAA; or (4) The research is conducted by, or on behalf of a Federal department or agency using government-generated or government-collected information obtained for non-research activities.
[ ] (d)(5) Research and demonstration projects which are conducted by or subject to the approval of federal department or agency heads, and which are designed to study public benefit or service programs for federally supported projects and most appropriately invoked with authorization by the funding agency, procedures for obtaining benefits or services under those programs, possible changes in or alternatives to those programs or possible changes in methods or levels of payment for benefits or services under those programs.
[ ] (d)(6) or 21 CFR 56.104(d) Taste and food quality evaluation.
[ ] No category fits what I want to do.

Will you be recording or keeping any of the 18 HIPAA identifiers?

[**x**] **Yes**
[ ] No

Indicate how appropriate protections are incorporated to ensure the privacy of subjects and confidentiality of data:

After the educational intervention has been delivered and prescribing data has been merged with educational participation data, all identifiers will be destroyed. To protect the participant confidentiality, only a limited data set will be created for analysis. Each participant will be given a unique identifier that contains no personal information. The PI will check the data for completeness after it has been extracted and verify that all subject identifiers (e.g. email or phone numbers for text messages) have been destroyed once the study is concluded. All data will be stored on password protected computers in a locked area at VUMC. This data will only be accessible by the PI and KSP.

All reasonable efforts will be made to keep a participant's information private and confidential. Data will be stored in secured areas on password protected Vanderbilt computers and servers conforming to the latest Vanderbilt IS security policies. Computer databases will be maintained on password protected Vanderbilt PCs. All study staff have completed employee education regarding patient confidentiality and have completed Human Subjects protection education (CITI course) as specified by the Vanderbilt IRB.

Describe any procedures to be used during the study:

As part of a VUMC educational initiative, two educational modules will be pushed out to prescribing providers. The first educational module consists of 20 questions around best practices for prescribing intravenous fluids, derived from the Isotonic Solutions and Major Adverse Renal Events Trial (SMART) and Saline against Lactated Ringer's or Plasma-Lyte in the Emergency Department (SALT-ED) trials, both published in the New England Journal of Medicine in 2018 and led by Vanderbilt investigators. Questions have been curated and reviewed by a panel of experts and piloted within VUMC for feasibility and acceptability. Similarly, an opioid prescribing model, Function First, Opioids Last, consisting of 20 questions designed by a panel of experts to increase knowledge around prescribing best practices, has been piloted within VUMC for feasibility and acceptability. Logistically, a question is delivered to a participant's smartphone each day and Continuing Medical Education credits are awarded for question completion. The completion of the module on intravenous fluids prescribing would therefore take approximately 4 weeks (1 question per day x 5 work days per week) and the module on opioid prescribing would take approximately 4 weeks to complete. Concepts are repeated as questions are strategically ordered throughout each module. Knowledge acquisition is postulated to occur through participation in answering the question, at which point a rational and opportunity to learn more about the topic is pushed to the participant's phone.

Will children be the subjects of your study?

[ ] Yes
[**x**] **No**

Date of IRB Approval: 08/26/2019
Institutional Review Board
VANDERBILT

Recruitment

Will the study provide compensation to research participants?

[ ] Yes
[**x**] **No**

PHI/Consent

Are you requesting a waiver of authorization to use/record protected health information?

[**x**] **Yes**
[ ] No

Please describe the plan to protect the identifiers from improper use and disclosure.

Identifiers are necessary to identify inpatient prescribing providers (name, role) and to contact them (email, text). After the educational intervention has been delivered and prescribing data has been merged with educational participation data, names and contact information will be deleted prior to analysis. To protect the participant confidentiality, only a limited data set will be created for analysis. Each participant will be given a unique identifier that contains no personal information. The PI will check the data for completeness after it has been extracted and verify that all subject identifiers (e.g. email or phone numbers for text messages) have been destroyed once the study is concluded. All data will be stored on password protected computers in a locked area at VUMC. This data will only be accessible by the PI and KSP.

Please describe how the privacy risks to individuals whose protected health/private information is to be used are reasonable in relation to the anticipated benefits (if any) and the importance of the knowledge expected from the research.

Identifiers are necessary to identify inpatient prescribing providers (name, role) and to contact them (email, text). Once the participant list has been curated, the prescribing data will be destroyed by the study team as a final de-identified dataset will be created for analysis after the educational intervention is deployed. Use of PHI for identification of eligible participants involves no more than minimal risk and this information will not be shared outside of the study team, prohibiting inappropriate use of prescribing data that could potentially adversely affect the privacy rights of individuals.This study has the potential to benefit participants through changing their prescribing patterns to align with best practices that have important quality implications for their practice and patients.

Please indicate the source of the PHI to be collected.

Provider identifiers are necessary to identify inpatient prescribing providers (name, role) and to contact them (email, text). Provider identification information will be collected when they register to participate in the educational exercise and prescribing behavior will be extracted from electronic medical records accordingly.

Testing the hypothesis that providing spaced education on appropriate fluid administration and opioid prescribing practices impacts provider prescribing behavior and by extension patient outcomes necessitates access to extractable information from within the provider's patients' medical records.

Please indicate when PHI will no longer be accessed.

Testing the hypothesis that providing spaced education on appropriate fluid administration and opioid prescribing practices impacts provider prescribing behavior and by extension patient outcomes necessitates access to extractable information from within the provider's patients' medical records. Provider and patient PHI will be maintained only as long as required to collect the outcome measurements from the electronic medical record, ensure providers receive appropriate CME credit, perform the study analyses, and publish the results. After that time, data collection will be concluded and the online database within REDCap will be modified to remove any protected health information.

Date of IRB Approval: 08/26/2019
Institutional Review Board
VANDERBILT

Please explain why the research could not practicably be conducted without accessing/using the protected health information:

Identifiers are necessary to identify inpatient prescribing providers (name, role) and to contact them (email, text). It would be impracticable to identify eligible providers without the waiver of alteration as there is no way to identify inpatient providers who have prescribed an opioid or IV fluid in the past 12 months without accessing prescribing data which contains PHI.

Please describe the plan to destroy the identifiers at the earliest opportunity consistent with the conduct of the research, unless there is a health or research justification for retaining the identifiers or such retention is otherwise required by law.

After the educational intervention has been delivered and prescribing data has been merged with educational participation data, names and contact information will be deleted prior to analysis. To protect the participant confidentiality, only a limited data set will be created for analysis. Each participant will be given a unique identifier that contains no personal information. The PI will check the data for completeness after it has been extracted and verify that all subject identifiers (e.g. email or phone numbers for text messages) have been destroyed once the study is concluded. All data will be stored on password protected computers in a locked area at VUMC. This data will only be accessible by the PI and KSP.

Please verify that the protected health information will not be reused or disclosed to any other person or entity, except as required by law, for authorized oversight of the research project, or for other research.

Protected health information will not be reused or disclosed to any other person or entity, except as required by law, for authorized oversight of the research project, or for other research.

Does this research disclose Protected Health Information (PHI)?

[ ] Yes
[**x**] **No**

Conflict of Interest Disclosure

Is there a potential conflict of interest for the Principal Investigator or key personnel? • The PI is responsible for assuring that no arrangement has been entered into where the value of the ownership interests will be affected by the outcome of the research and no arrangement has been entered into where the amount of compensation will be affected by the outcome of the research. • Assessment should include anyone listed as Principal Investigator, or other research personnel on page 1 of this application. Please note that ownership described below apply to the aggregate ownership of an individual investigator, his/her spouse, domestic partner and dependent children). Do not consider the combined ownership of all investigators.

[ ] Yes
[**x**] **No**

Detailed Protocol Narrative
Full Study Title: Education of providers on prescribing best practices
Primary Investigator: Matthew McEvoy, MD

1. Background

Implementation of known best practices remains suboptimal in healthcare with an average time from knowledge gained to implementation in practice settings noted as 17 years. To bridge this implementation gap, we need mechanisms for timely dissemination and education on the latest research in a convenient way that engages busy healthcare providers.

Spaced Education can be an impactful approach to improving knowledge acquisition, with studies even noting a change in clinical practice patterns.[1-7] Furthermore, the development of QuizTime, a smartphone application created at Vanderbilt, has ushered in the opportunity to deliver Spaced Education with unprecedented convenience. This initiative aims to utilize QuizTime to deliver Spaced Education around best practices on two very timely topics: opioid prescribing and prescribing of intravenous fluids.

2. Purpose

This study aims to evaluate the impact of spaced education, delivered via a smartphone application, on provider prescribing patterns.

3. Protocol Methodology

Part I: Educational initiative

As part of a VUMC educational initiative, two educational modules will be pushed out to prescribing providers. The first educational module consists of 20 questions around best practices for prescribing intravenous fluids, derived from the Isotonic Solutions and Major Adverse Renal Events Trial (SMART) and Saline against Lactated Ringer's or Plasma-Lyte in the Emergency Department (SALT-ED) trials, both published in the New England Journal of Medicine in 2018 and led by Vanderbilt investigators. Questions have been curated and reviewed by a panel of experts and piloted within VUMC for feasibility and acceptability. Similarly, an opioid prescribing model, Function First, Opioids Last, consisting of 20 questions designed by a panel of experts to increase knowledge around prescribing best practices, has been piloted within VUMC for feasibility and acceptability. Logistically, a question is delivered to a participant's smartphone each day and Continuing Medical Education credits are awarded for question completion. The completion of the module on intravenous fluids prescribing would therefore take approximately 4 weeks (1 question per day x 5 work days per week) and the module on opioid prescribing would take approximately 4 weeks to complete. Concepts are repeated as questions are strategically ordered throughout each module. Knowledge acquisition is postulated to occur through participation in answering the question, at which point a rational and opportunity to learn more about the topic is pushed to the participant's phone.

Part II Evaluation:

While evidence exists around the impact of Spaced Education on knowledge acquisition and pilot data has demonstrated the feasibility of using QuizTime as a delivery mechanism for this type of education, the impact of Spaced Education on prescribing behaviors, particularly at an institutional level, has not been demonstrated. This study aims to evaluate the impact of spaced education, delivered via a smartphone application, on provider prescribing patterns.

In order to account for potential confounders in observed prescribing trends of either intravenous fluids or opioids over time, education will be pushed to a random half of eligible prescribers on intravenous fluids

while education will be pushed to the other randomized half on opioid prescribing best practices (Table 1). This ensures two key points:

1. **Every prescriber will receive both educational modules, ensuring that while randomization will be used to determine the order of education received, the education will not be withheld from any group.**

2. **Changes in prescribing behaviors can be attributed to the delivered education as each group serves as a comparison group.** Evidence shows that individuals receiving any intervention, such as education, behave differently from individuals who are not receiving the intervention. This design allows for comparison of prescribing rates of opioids among those participating in the opioid educational module to prescribers who are not participating in the opioid educational module BUT who are also receiving similar education. This holds for intravenous fluids as well.

Using data from VUMC's electronic health records, prescribing data for both opioids and intravenous fluids will be analyzed for all participating prescribers. Associations between completion of QuizTime questions, the act of which is postulated to improve knowledge acquisition, and prescribing behaviors will then be made. Additionally, trends in prescribing behaviors will be compared to the group receiving the other educational module in order to control for confounding factors over time.

Probable Duration

The duration of this initiative is anticipated to be 1 year to complete both education and evaluation.

4. Location of Study

Vanderbilt University Medical Center

5. Special Precautions

There will be no medical procedures performed on the participants as part of the study. Using questions for learning and knowledge assessment contains no actual risk to the participant as user performance will not be shared with employers or national licensing boards. There is a potential for a participant to experience emotional stress from the questions. As such, each question contains a field where they can send feedback or concerns to the study team.

Identifiers are necessary to identify inpatient prescribing providers (name, role) and to contact them (email, text). After the educational intervention has been delivered and prescribing data has been merged with educational participation data, names and contact information will be deleted prior to analysis. To protect the participant confidentiality, only a limited data set will be created for analysis. Each participant will be given a unique identifier that contains no personal information. The PI will check the data for completeness

after it has been extracted and verify that all subject identifiers (e.g. email or phone numbers for text messages) have been destroyed once the study is concluded. All data will be stored on password protected computers in a locked area at VUMC. This data will only be accessible by the PI and KSP.

6. Experimental Controls

This is a cohort study with a cross-over design; therefore, individuals will be assigned to both an intervention and control group.

7. Type and Number of Subjects

All inpatient prescribing providers who have prescribed EITHER an opioid or intravenous fluid in the past 12 months at VUMC are eligible.

8. Data Sources

Data will be extracted from the QuizTime application regarding participation in the educational modules.

Prescribing data from the EHR will be merged with this data to evaluate impact on behaviors. VAPIR and VUMC HealthIT partners will provide the dataset(s) to the study team after removing provider-level identifiers for analysis.

To determine eligibility, data from the EHR, via the Electronic Data Warehouse, will be extracted and analyzed according to VAPIR and CTSA security protocols to identify eligible providers for recruitment (inpatient prescribing providers who have prescribed either an opioid or IV fluids in the past 12 months). Eligible providers will be identified and reached out to via email for recruitment into the study, making access of PHI necessary.

9. Statistical Analysis

This study is designed to integrate two separate randomized controlled trials, one for opioid education and the other for fluid education. Participants are randomly allocated to receive fluids education or opioid training. Training runs for four weeks. Then, there is a four-week gap during which we will measure prescribing practices for both fluids and opioids. Then, we will switch and those who previously got fluids will now get opioids training and vice versa. A four-week data collection period will follow.

For each trial, each provider will contribute three measurements to the analysis reflecting prescribing behavior: the four weeks prior to education, the four weeks after the initial intervention period when half of the participants have been exposed to training, and then the four weeks after second intervention period when all participants have been exposed to the training, but one half received their training a month prior so that we can explore whether training effects are sustained. The measurement of interest for the fluids education trial will be proportion of total fluids ordered that are for balanced crystalloids during the four-week period. For the opioids education trial, it will be the proportion of patient visits attended that resulted in an opioid prescription, and secondarily the average MME prescribed. We have used a summary statistic to generate a measure of prescribing behavior at the level of the provider (e.g. proportion of orders). This is because we expect providers will vary greatly in their patient volume, types of patients seen, and opportunity for prescribing.

The design for both trials is similar. We expect that prior to any training, prescribing practices are similar between groups. We expect that after the first four weeks of training, fluid prescribing practices will favor the use of balanced crystalloids among the group receiving fluids training and that there will be a reduction in the prescribing of opioids in the intervention group. We expect that after both groups have received training, prescribing practices will no longer differ between groups.

Initial analyses will characterize the provider cohort and uptake of education. We will also describe our outcome variables graphically and using summary statistics.

We will next use a mixed effects proportional odds model to model ordering practices as a function of period (baseline, after the first group is trained, and after both groups are trained). We will include subject

as a random effect to take into account the repeated measurement. The primary variables will include whether or not training has been completed, and the period. We do not expect to observe a systematic change over time that is unaccounted for by training. We will separately model, instead of training, which group the provider is in. In this model, we will test the interaction between group and period as we expect differences between groups to be minimal before and after, and maximal when only one of the groups has undergone training. We do expect differences between the last period and the first period in this model, with the latter time periods favoring balanced crystalloids (for fluids) and reduced opioid prescribing (for opioids).

STATA and R will be used to analyze the data with a p-value of 0.05 deemed statistically significant.

10. Potential Risks and Benefits

Benefits: This study has the potential to reveal successful approaches to improving quality of care through provider-based education.

Risks: All risk identified in this study relates to a potential breach of confidentiality. All information will be handled and stored according to applicable rules and regulations.

11. Monitoring of Safety

Concerns expressed by participants will be monitored daily throughout the study. These can include technical problems or concerns as noted above under ‘Risks.’ All events will be monitored until resolution. If a concern expressed by a participant remains unresolved at the conclusion of the study, the investigator will assess whether continued follow-up of the event is warranted. In those cases where the investigator deems that there is a need for further follow-up, the investigator will establish a plan for follow-up and communicate that directly to the study subject.

12. Payment

There will be no compensation offered to participants.

13. Obtain and Record Informed Consent

This is a secondary analysis of already existing data. As such, we are requesting a waiver of consent.

14. Confidentiality

All reasonable efforts will be made to keep a participant’s information private and confidential. Data will be stored in secured areas on password protected Vanderbilt computers and servers conforming to the latest Vanderbilt IS security policies. Computer databases will be maintained on password protected Vanderbilt PCs. All study staff have completed employee education regarding patient confidentiality and have completed Human Subjects protection education (CITI course) as specified by the Vanderbilt IRB.

15. Recruiting Methods

Faculty listservs will be used in accordance with institutional educational protocols to recruit eligible participants to enroll via REDCap. Subsequently participant enrollment data will be pushed into the QuizTime application.

16. How patient’s primary physician will be notified

The patient’s primary physician will not be notified.

17. Anticipated Coordination

We do not anticipate any coordination necessary between our team and the primary physician.

18. Pregnancy Test

This will not be necessary to this study.

19. Rational for excluding women, minorities and/or children from participation

There is no intended exclusion of any of these groups.

Citations

1. Kerfoot BP, Baker HE, Koch MO, Connelly D, Joseph DB, Ritchey ML. Randomized, controlled trial of spaced education to urology residents in the United States and Canada. *J Urol.* 2007;177(4):1481-1487.
2. Kerfoot BP, DeWolf WC, Masser BA, Church PA, Federman DD. Spaced education improves the retention of clinical knowledge by medical students: a randomised controlled trial. *Med Educ.* 2007;41(1):23-31.
3. Kerfoot BP, Armstrong EG, O'Sullivan PN. Interactive spaced-education to teach the physical examination: a randomized controlled trial. *J Gen Intern Med.* 2008;23(7):973-978.
4. Kerfoot BP. Learning benefits of on-line spaced education persist for 2 years. *J Urol.* 2009;181(6):2671-2673.
5. Matzie KA, Kerfoot BP, Hafler JP, Breen EM. Spaced education improves the feedback that surgical residents give to medical students: a randomized trial. *Am J Surg.* 2009;197(2):252-257.
6. Kerfoot BP, Baker H. An online spaced-education game to teach and assess residents: a multi-institutional prospective trial. *J Am Coll Surg.* 2012;214(3):367-373.
7. Kerfoot BP, Turchin A, Breydo E, Gagnon D, Conlin PR. An online spaced-education game among clinicians improves their patients' time to blood pressure control: a randomized controlled trial. *Circ Cardiovasc Qual Outcomes.* 2014;7(3):468-474.

QuizTime analysis plan

This study is designed to integrate two separate randomized controlled trials, one for opioid education and the other for fluid education. Participants are randomly allocated to receive fluids education or opioid training. Training runs for four weeks. Then, there is a four-week gap during which we will measure prescribing practices for both fluids and opioids. Then, we will switch and those who previously got fluids will now get opioids training and vice versa. A four-week data collection period will follow.

For each trial, each provider will contribute three measurements to the analysis reflecting prescribing behavior: the four weeks prior to education, the four weeks after the initial intervention period when half of the participants have been exposed to training, and then the four weeks after second intervention period when all participants have been exposed to the training, but one half received their training a month prior so that we can explore whether training effects are sustained. The measurement of interest for the fluids education trial will be proportion of total fluids ordered that are for balanced crystalloids during the four-week period. For the opioids education trial, it will be the proportion of patient visits attended that resulted in an opioid prescription, and secondarily the average MME prescribed. We recognize that we have used a summary statistic to generate a measure of prescribing behavior at the level of the provider (e.g. proportion of orders). This is because we expect providers will vary greatly in their patient volume, types of patients seen, and opportunity for prescribing.

The design for both trials is similar. We expect that prior to any training, prescribing practices are similar between groups. We expect that after the first four weeks of training, fluid prescribing practices will favor the use of balanced crystalloids among the group receiving fluids training and that there will be a reduction in the prescribing of opioids in the intervention group. We expect that after both groups have received training, prescribing practices will no longer differ between groups.

Initial analyses will characterize the provider cohort and uptake of education. We will also describe our outcome variables graphically and using summary statistics.

We will next use a mixed effects proportional odds model to model ordering practices as a function of period (baseline, after the first group is trained, and after both groups are trained). We will include subject as a random effect to take into account the repeated measurement. The primary variables will include whether or not training has been completed, and the period. We do not expect to observe a systematic change over time that is unaccounted for by training. We will separately model, instead of training, which group the provider is in. In this model, we will test the interaction between group and period as we expect differences between groups to be minimal before and after, and maximal when only one of the groups has undergone training. We do expect differences between the last period and the first period in this model, with the latter time periods favoring balanced crystalloids (for fluids) and reduced opioid prescribing (for opioids).
