# Supplementary material for: Effect of Smartphone App–Based Education on Clinician Prescribing Habits in a Learning Health Care System: A Randomized Cluster Crossover Trial
Source: JAMA Netw Open. 2022 Jul 26;5(7):e2223099. doi: 10.1001/jamanetworkopen.2022.23099 (PMC9327570; doi:10.1001/jamanetworkopen.2022.23099)
Supplement: Supplement 2. — eAppendix 1. Communication Announcement eAppendix 2. QuizTime Module eFigure. Example of QuizTime User Interface eTable. Learner Analytics [file jamanetwopen-e2223099-s002.pdf]

Supplemental Online Content

McEvoy MD, Dear ML, Buie R, et al; Vanderbilt Learning Healthcare System Platform Investigators; Vanderbilt Committee on Opioid Monitoring and Stewardship. Effect of smartphone app–based education on clinician prescribing habits in a learning health care system: a randomized cluster crossover trial. *JAMA Netw Open.* 2022;5(7):e2223099. doi:10.1001/jamanetworkopen.2022.23099

eAppendix 1. Communication Announcement

eAppendix 2. QuizTime Module

eFigure. Example of QuizTime User Interface

eTable. Learner Analytics

This supplemental material has been provided by the authors to give readers additional information about their work.

© 2022 McEvoy MD et al. *JAMA Network Open.*

Communication Announcement

All VUMC prescribers,

In order to facilitate timely dissemination of the latest evidence among busy healthcare providers, we are offering text-based learning on two important topics: best practices for opioid prescribing* and use of intravenous fluids. By answering one question per day (via your smartphone or email) you can keep up to date with the latest guidelines while earning CME credit (1hr CME credit/4 questions answered). Upon enrolling in this educational study, you will receive both of the educational modules (opioid prescribing and intravenous fluids), but the order of delivery will be randomized so we can learn about the impact of this educational approach.

No information concerning your participation will be shared with your department or employer. Please click on the link <insert link> to sign up. Additional reminders will be sent from learninghealth@vumc.org.

This project is supported by the Learning Healthcare System Platform. Essential to a Learning Healthcare System is the timely dissemination and implementation of clinically relevant, actionable findings. Your participation will help bridge the gap from discovery to practice as we continue to learn from what we do and do what we learn.

Thank you for participating in this process as we work to close the loop in the research/care delivery cycle.

Sincerely,

The Learning Healthcare System team

* The opioid-prescribing module will fulfill the state requirement from the Tennessee Board of Medical Examiners. The questions in this module will be based on similar principles to other offerings from the QuizTime education group but contain updated content.
* Vanderbilt University Medical Center is accredited by the Accreditation Council for Continuing Medical Education to provide continuing medical education for physicians. Vanderbilt University Medical Center designates this enduring material for a maximum of 5 credits *AMA PRA Category 1 Credit(s)*™. Physicians should claim only the credit commensurate with the extent of their participation in the activity.

© 2022 McEvoy MD et al. *JAMA Network Open.*

QuizTime Module:

Appropriate Use of Intravenous Fluids for Adult Patients
in the Emergency Department and In-Hospital Setting:
Follow-up Education to SMART and SALT-ED Trials

Primarily based upon:
Semler MW et al. *NEJM*, 2018
Self WH et al. *NEJM,* 2018

Topic: Definition of MAKE 30 as an outcome

Question 1: Which of the following is the definition of MAKE 30 (Major Adverse Kidney Events at 30 days)?

Answers:

- **A.** Composite of death and new renal replacement therapy within 30 days of ED or ICU admission
- **B.** Composite of death, new renal replacement therapy, or serum creatinine ≥3mg/dL within 30 days of ED or ICU admission
- **C.** Composite of new renal replacement therapy or final serum creatinine ≥300% of baseline within 30 days of ED or ICU admission
- **D.** Composite of death, new renal replacement therapy, or final serum creatinine ≥200% of baseline within 30 days of ED or ICU admission

© 2022 McEvoy MD et al. *JAMA Network Open.*

Correct Answer: D

Key Point: The outcome “Major Adverse Kidney Events at 30 days” (MAKE 30) includes death, new renal replacement therapy, or serum creatinine ≥ 200% of baseline at discharge or 30 days after admission

Rationale:
In the SMART and SALT-ED trials, Major Adverse Kidney Events within 30 days (MAKE30) was defined as a composite of death, new renal replacement therapy, or final serum creatinine ≥ 200% of baseline at the earliest of either hospital discharge or 30 days after the index ED visit or admission to the ICU. MAKE30 was a dichotomous outcome. All patients in the trial were eligible for MAKE30. Patients who presented to the ED with end stage renal disease and undergoing chronic renal replacement therapy were eligible for MAKE30 only through death. All other patients were eligible for MAKE30 through all three components—death, new renal replacement therapy, and final serum creatinine ≥ 200% of baseline.

References

Self WH, et al. Balanced Crystalloids versus Saline in Noncritically Ill Adults. N Engl J Med. 2018;378:819-828

Semler MW, et al. Balanced Crystalloids versus Saline in Critically Ill Adults. N Engl J Med. 2018;378:829-839

© 2022 McEvoy MD et al. *JAMA Network Open.*

Topic: Effect of balanced crystalloid vs. saline on MAKE 30 in SALT-ED and SMART

Question 2: What is the effect on major adverse kidney events at 30 days after admission (MAKE 30) when hospitalized adult patients receive intravenous Plasma-lyte A instead of normal saline?

Answers:

- **A.** Increased rate of MAKE 30 with Plasma-Lyte A
- **B.** Decreased rate of MAKE 30 with Plasma-Lyte A
- **C.** No difference in the rate of MAKE 30 with Plasma-Lyte A

Correct Answer: B

Key Point: Based upon the recently published SALT-ED and SMART trials, administration of balanced crystalloid solutions (Plasmalyte-A, Normosol, Lactated Ringer's) causes **a lower rate of MAKE 30 as compared to administration of normal saline.**

Rationale:
In the SMART and SALT-ED trials, Major Adverse Kidney Events within 30 days (MAKE30) was defined as a composite of death, new renal replacement therapy, or final serum creatinine ≥ 200% of baseline at the earliest of either hospital discharge or 30 days after the index ED visit or admission to the ICU. MAKE30 was a dichotomous outcome. All patients in the trial were eligible for MAKE30. Patients who presented to the ED with end stage renal disease and undergoing chronic renal replacement therapy were eligible for MAKE30 only through death. All other patients were eligible for MAKE30 through all three components—death, new renal replacement therapy, and final serum creatinine ≥ 200% of baseline. Using this metric to assess the effects of balanced crystalloids versus normal saline, the use of balanced crystalloids resulted in a lower rate of MAKE30.

References

Self WH, et al. Balanced Crystalloids versus Saline in Noncritically Ill Adults. N Engl J Med. 2018;378:819-828

Semler MW, et al. Balanced Crystalloids versus Saline in Critically Ill Adults. N Engl J Med. 2018;378:829-839

© 2022 McEvoy MD et al. *JAMA Network Open.*

Topic: Effect of balanced crystalloid vs. saline on MAKE 30 in septic patients in SALT-ED and SMART

Question 3: What is the effect on MAKE 30 (major adverse kidney events at 30 days after admission) of administering Lactated Ringers to adult patients with sepsis as compared to normal saline?

Answers:

- **A.** Increased rate of MAKE 30 with Lactated Ringers
- **B.** Decreased rate of MAKE 30 with Lactated Ringers
- **C.** No difference in the rate of MAKE 30 between groups

Correct Answer: B

Key Point: Based upon the recently published SMART trial, administration of balanced crystalloid solutions (Plasmalyte-A, Normosol, Lactated Ringer's) causes a lower rate of MAKE 30 as compared to administration of normal saline, especially in patients admitted to the hospital with sepsis.

Rationale: While the exact cause is unknown, administration of balanced crystalloid solutions (Plasmalyte-A, Normosol, Lactated Ringer's) causes a lower rate of MAKE 30 as compared to administration of normal saline, especially in patients admitted to the hospital with sepsis. This was recently demonstrated in the SALT-ED and SMART trials, which together randomized ~28,000 patients to receive either normal saline or a balanced crystalloid solution. The studies did show similar findings to prior research concerning the biochemical alterations associated with normal saline administration – namely, higher chloride values and lower bicarbonate values, the former of which has been strongly associated with renal injury.

References

Self WH, et al. Balanced Crystalloids versus Saline in Noncritically Ill Adults. N Engl J Med. 2018;378:819-828

Semler MW, et al. Balanced Crystalloids versus Saline in Critically Ill Adults. N Engl J Med. 2018;378:829-839

© 2022 McEvoy MD et al. *JAMA Network Open.*

Topic: Effect of balanced crystalloid vs. saline on MAKE 30 in TBI patients in the SMART trial

Question 4: What is the effect on MAKE 30 (major adverse kidney events at 30 days after admission) of administering normal saline to adult patients with traumatic brain injury as compared to a balanced crystalloid solution?

Answers:

- **A.** Increased rate of MAKE 30 with normal saline
- **B.** Decreased rate of MAKE 30 with normal saline
- **C.** No difference in the rate of MAKE 30 between groups

Correct Answer: C

Key Point: Based upon the recently published SMART trial, there was no difference in the rate of MAKE 30 with administration of normal saline versus balanced crystalloid solutions in patients with traumatic brain injury (TBI).

Rationale: For critically ill patients with a diagnosis of traumatic brain injury (TBI), there was no difference in the rate of MAKE 30 with administration of normal saline versus balanced crystalloid solutions in the recent SMART trial, a large, prospective RCT. In fact, some evidence suggests improved outcomes with normal saline administration in patients with TBI who undergo surgery. At this time, the exact effect of crystalloid administration on patient outcomes in the setting of TBI is not known, but the evidence appears to favor using normal saline.

References

Semler MW, et al. Balanced Crystalloids versus Saline in Critically Ill Adults. N Engl J Med. 2018;378:829-839

Rowell SE, et al. The Impact of Pre-Hospital Administration of Lactated Ringer's Solution versus Normal Saline in Patients with Traumatic Brain Injury. J Neurotrauma. 2016;33:1054-9.

Topic: Composition and pH of IV fluids and definition of balanced crystalloid solutions

Question 5: Which of the following represents the elemental composition of 1 liter of Plasma-Lyte A?

Answers:

- **A.** Na 154 mEq / Cl 154 mEq / K 0 mEq / Mg 0 mEq / Ca 0 mEq

© 2022 McEvoy MD et al. *JAMA Network Open.*

B. Na 145 mEq / Cl 110 mEq / K 4 mEq / Mg 0 mEq / Ca 0 mEq
C. Na 140 mEq / Cl 98 mEq / K 5 mEq / Mg 3 mEq / Ca 0 mEq
D. Na 130 mEq / Cl 109 mEq / K 4 mEq / Mg 2 mEq / Ca 1.4 mEq

Correct Answer: C

Key Point: Normal saline (NS) contains only sodium and chloride in supraphysiologic levels and is hyperosmolar (308 Osm) compared to human plasma (290 Osm). In contrast, **balanced crystalloid solutions, such as Lactated Ringer's (LR) or Plasma-Lyte A (PLA, also known as Normosol) have electrolyte compositions closer to that of human plasma.**

Rationale: PLA contains sodium, chloride, potassium, and magnesium very similar to plasma and is iso-osmolar compared to plasma due to the addition of acetate and gluconate. LR is slightly hypo-osmolar, hyponatremic, and hyperchloremic compared to human plasma and also contain calcium. LR also contains sodium lactate as a buffer. See the chart below for complete details.

Of note, LR may slightly increase plasma lactate levels. This is not due to lactic acid production, but rather a product of the sodium lactate in LR. In addition, neither LR or PLA appreciably change glucose, bicarbonate, or lactate levels. Finally, the potassium of LR is 4 mEq/L compared to that of PLA which is 5 mEq/L. Neither solution has ever been shown to raise potassium levels.

	IV Fluid			
**(mEq/L)**	**Plasma**	**NS**	**LR**	**PLA**
**pH**	**7.4**	**5-5.5**	**6.5**	**7.4**
**Na**	135-145	154	130	140
**K**	3.5-5.0	0	4	5
**Cl**	98-104	154	109	98
**Mg**	0.8-1.2	0	0	3
**Ca**	2.2-2.6	0	1.4	0
**Lactate**	-	-	28	-
**Acetate**	-	-	-	27
**Gluconate**	-	-	-	23
**Osm**	285-295	308	274	295
**SIDa**	35	0	21	42

References:

Weinberg L, et al. Plasma-Lyte 148: A clinical review. World J Crit Care Med. 2016;5:235-250.

© 2022 McEvoy MD et al. *JAMA Network Open.*

Topic: pH of common intravenous solutions
Question 6: What are the pH and apparent strong ion difference of normal saline (0.9% NaCl)?
Answers:

- **A.** 7.4 and 35
- **B.** 6.5 and 21
- **C.** 5.5 and 0
- **D.** 7.4 and 42

Correct Answer: C

Key Point: Normal saline is very acidic (pH 5.0-5.5) with an apparent strong ion difference (SIDa) of 0, while Plasma-Lyte A has a pH equal to human plasma (7.4) and a SIDa of 42 (normal is 35).

Rationale: In addition to pH, which is based on the Henderson-Hasselbach equation, the strong ion difference (SID) is another method for estimating the acid-base status of human plasma or an IV solution. It is based on the Stewart method and considers strong ions those that are >99% dissociated at physiologic pH. Sodium and chloride account for most of the SID, and the difference between the two is considered the apparent SID (SID_a) because it estimates the overall SID very well. Normal SID_a is 35. If the SID_a is >35, then the solution is alkalotic compared to human plasma; if <35, then the solution is acidotic. Normal saline is very acidic (pH 5.0-5.5) with an apparent strong ion difference (SIDa) of 0, while Plasma-Lyte A has a pH equal to human plasma (7.4) and a SIDa of 42 (normal is 35), making it somewhat alkalotic. Lactated Ringer's is in the middle with a pH of 6.5 and SID_a of 21.

	IV Fluid			
	**Plasma**	**NS**	**LR**	**PLA**
**pH**	**7.4**	**5-5.5**	**6.5**	**7.4**
**Na**	135-145	154	130	140
**K**	3.5-5.0	0	4	5
**Cl**	98-104	154	109	98
**Mg**	0.8-1.2	0	0	3
**Ca**	2.2-2.6	0	1.4	0
**Lactate**	-	-	28	-
**Acetate**	-	-	-	27
**Gluconate**	-	-	-	23
**Osm**	285-295	308	274	295
**SIDa**	35	0	21	42

all electrolytes in mEq/L

References:
Weinberg L, et al. Plasma-Lyte 148: A clinical review. World J Crit Care Med. 2016;5:235-250.

© 2022 McEvoy MD et al. *JAMA Network Open.*

Topic: Risks of hyperchloremia

Question 7: A 62-year-old female presents for exploratory laparotomy for a small bowel obstruction. During the operation, she is given 1750 mL (20mg/kg) of normal saline (0.9% NaCl). Postoperatively, her labs reveal the following:

Na	138 mEq/L
K	4.5 mEq/L
Cl	114 mEq/L
HCO_3	17 mEq/L
BUN	14 mg/dL
Cr	1.1 mg/dL
Glc	103 mg/dL

Based on the chloride level, the patient is at increased risk for which of the following?

Answers:

- **A.** Death
- **B.** Pulmonary embolus
- **C.** Atrial fibrillation
- **D.** Myocardial Infarction

Correct Answer: A

Key Point: Patients administered normal saline during non-cardiac surgery are at increased risk for hyperchloremia (Cl > 110 mEq/L) which is associated with acute kidney injury and death when compared to patients administered isotonic crystalloids such as Plasma-Lyte A or Lactated Ringers. Studies have also reported this association in septic patients.

Rationale: Historically, normal saline (NS) has been the most commonly administered intravenous fluid. Data suggest that intravenous NS may be associated with hyperchloremic metabolic acidosis, acute kidney injury (AKI), and death. Patients who receive NS either after elective non-cardiac surgery or in the setting of sepsis are more likely to develop hyperchloremia (Cl>110 mEq/L). Importantly, the development of hyperchloremia itself has been associated with worse outcomes, including increased AKI and death. The likelihood of developing hyperchloremia is lower when balanced crystalloids (LR or PLA) are administered.

References:

McCluskey SA, et al. Hyperchloremia after noncardiac surgery is independently associated with increased morbidity and mortality: a propensity-matched cohort study. Anesth Analg 2013;117:412-21.

Raghunathan K, et al. Association between the choice of IV crystalloid and in-hospital mortality among critically ill adults with sepsis. Crit Care Med 2014;42:1585-91.

© 2022 McEvoy MD et al. *JAMA Network Open.*

Shaw AD, et al. Association between intravenous chloride load during resuscitation and in-hospital mortality among patients with SIRS. Intensive Care Med. 2014;40:1897-905.

© 2022 McEvoy MD et al. *JAMA Network Open.*

Topic: Effect of IV fluids on serum potassium and acid-base status

Question 8: Administration of which intravenous crystalloid solution is MOST likely to increase serum potassium?

Answers:

- **A.** Lactated Ringers
- **B.** Normal saline
- **C.** Normosol
- **D.** Plasma-lyte A

Correct Answer: B

Key Point: Administration of normal saline has been shown in multiple high-quality studies to cause more hyperkalemia compared to other crystalloids.

Rationale: Administration of normal saline has been shown in multiple high-quality studies to cause more hyperkalemia compared to other crystalloids. In contrast, there is no evidence that any potassium-containing solution (e.g. LR or PLA/Normosol) causes an increase in potassium, including patients with end-stage renal disease. There is some evidence that normal saline may cause hyperkalemia requiring treatment in patients with end-stage renal disease undergoing transplantation, whereas Lactated Ringer’s and Plasma-Lyte do not.

References:

Gonzalez-Castro A, et al. Influence of Proportion of Normal Saline Administered in the Perioperative Period of Renal Transplantation on Kalemia Levels. Transplant Proc. 2018;50:569-571

O'Malley CM, et al. A randomized, double-blind comparison of lactated Ringer's solution and 0.9% NaCl during renal transplantation. Anesth Analg. 2005; 100:1518-24.

Weinburg L. Effects of intraoperative and early postoperative normal saline or Plasma-Lyte 148® on hyperkalaemia in deceased donor renal transplantation: a double-blind randomized trial. Br J Anaesth, 2017;119:606-615.

© 2022 McEvoy MD et al. *JAMA Network Open.*

Topic: Effect of hyperchloremia on renal perfusion and urine output

Question 9: Which of the following intravenous fluids is MOST likely to decrease renal artery blood flow and renal cortical perfusion in humans?

Answers:

- **A.** Albumin 5%
- **B.** Lactated Ringers
- **C.** Normal saline
- **D.** Plasma-lyte A

Correct Answer: C

Key Point: Two trials in humans have shown that normal saline reduces renal artery flow and renal cortical perfusion while delaying urine output and resulting in more weight gain compared to balanced solutions (Plasma-Lyte A or Lactated Ringer's).

Rationale: Based upon a number of studies showing an association of renal dysfunction after normal saline administration, two prospective randomized, controlled, double-blind crossover trials were performed in humans to evaluate the effects of different crystalloid solutions on renal perfusion and urine output. These trials demonstrated that normal saline caused a reduction in renal artery flow and renal cortical perfusion, and also caused delayed urine output and more weight gain as compared to balanced solutions.

References:

Reid F, et al. (Ab)normal saline and physiological Hartmann's solution: a randomized double-blind crossover study. Clin Sci (Lond). 2003;104:17-24.

Chowdhury AH, et al. A Randomized, Controlled, Double-Blind Crossover Study on the Effects of 2-L Infusions of 0.9% Saline and Plasma-Lyte 148 on Renal Blood Flow Velocity and Renal Cortical Tissue Perfusion in Healthy Volunteers. Ann Surg. 2013;258:1118.

© 2022 McEvoy MD et al. *JAMA Network Open.*

Topic: Compatibility of fluids with blood and medications

Question 10: A 73-year-old woman presents with severe anemia due to an upper GI bleed and requires packed red blood cell transfusion. Which crystalloid solution should NOT be administered in the same IV line as transfused blood?

Answers:

- **A.** Normal Saline
- **B.** Plasma-Lyte A
- **C.** Lactated Ringer's
- **D.** Normal Saline with 5% dextrose

Correct Answer: C

Key Point: Lactated Ringer's (LR) is not compatible with packed red blood cell transfusion.

Rationale: The calcium content in LR binds the preservative citrate, causing clotting/precipitation with blood products. Therefore, Lactated Ringer's (LR) is not compatible with packed red blood cell transfusion. Normal saline, Plasma-Lyte A, and other solutions that lack calcium are compatible with blood co-administration.

References:

Strautz RL, et al. Compatibility of ADSOL-stored red cells with intravenous solutions. Am J Emerg Med, 1989;7:162-4.

© 2022 McEvoy MD et al. *JAMA Network Open.*

Topic: Effect of balanced crystalloid vs. saline on chloride and acidemia in SALT-ED and SMART

Question 11:, Which of the following changes in serum chemistry are MOST likely to occur if a patient is administered normal saline instead of a balanced crystalloid?

Answers:

- **A.** Increased chloride and increased bicarbonate
- **B.** Increased chloride and decreased bicarbonate
- **C.** Decreased chloride and decreased bicarbonate
- **D.** Decreased chloride and increased bicarbonate

Correct Answer: B

Key Point: Based upon the recently published SALT-ED and SMART trials, administration of normal saline results in higher chloride and lower bicarbonate levels versus Plasma-Lyte A or other balanced crystalloid solutions. The magnitude of the effect is volume/dose related.

Rationale: Numerous studies have shown that the development of hyperchloremia is associated with worse renal outcomes and increased mortality. Based upon these trials, administration of normal saline results in higher chloride and lower bicarbonate levels versus Plasma-Lyte A or other balanced crystalloid solutions. The magnitude of the effect is volume/dose related. Furthermore, the administration of normal saline was associated with increased renal injury and death.

References

Self WH, et al. Balanced Crystalloids versus Saline in Noncritically Ill Adults.
N Engl J Med. 2018;378:819-828

Semler MW, et al. Balanced Crystalloids versus Saline in Critically Ill Adults. N Engl J Med. 2018;378:829-839

McCluskey SA, et al. Hyperchloremia after noncardiac surgery is independently associated with increased morbidity and mortality: a propensity-matched cohort study. Anesth Analg 2013;117:412-21.

Raghunathan K, et al. Association between the choice of IV crystalloid and in-hospital mortality among critically ill adults with sepsis. Crit Care Med 2014;42:1585-91.

© 2022 McEvoy MD et al. *JAMA Network Open.*

Shaw AD, et al. Association between intravenous chloride load during resuscitation and in-hospital mortality among patients with SIRS. Intensive Care Med. 2014;40:1897-905.

© 2022 McEvoy MD et al. *JAMA Network Open.*

Topic: Effect of balanced crystalloid vs. saline on MAKE 30 with PREVIOUS RRT

Question 12: A 55-year-old man with ESRD on hemodialysis presents to the emergency department for evaluation of diarrhea and fatigue. Although IV fluid therapy with normal saline is initially ordered, the order is cancelled and the patient is given Plasma-Lyte A instead. What effect on clinical outcomes is MOST likely to result from a change to Plasma-Lyte A?

Answers:

- **A.** Increased risk of mortality
- **B.** Decreased risk of mortality
- **C.** Decreased risk of acidosis requiring treatment
- **D.** Increased risk of hyperkalemia requiring treatment

Correct Answer: B

Key Points: In the SMART trial, patients with a history of renal replacement therapy prior to enrollment in the study had an increased odds ratio of in-hospital mortality when administered normal saline instead of a balanced crystalloid solution.

Rationale: For critically ill patients with a history of renal replacement therapy prior to enrollment in the SMART trial, the odds ratio of in-hospital mortality was greatly reduced (0.61 [95% CI 0.41-0.91, p=0.01]) for patients who received balanced crystalloids rather than normal saline. The exact mechanism of this is not known. However, a number of studies have shown less biochemical perturbations for surgical patients with end-stage renal disease who received a balanced crystalloid rather than normal saline.

References:

Semler MW, et al. Balanced Crystalloids versus Saline in Critically Ill Adults. N Engl J Med. 2018;378:829-839

O'Malley CM, et al. A randomized, double-blind comparison of lactated Ringer's solution and 0.9% NaCl during renal transplantation. Anesth Analg. 2005; 100:1518-24.

Weinburg L. Effects of intraoperative and early postoperative normal saline or Plasma-Lyte 148® on hyperkalaemia in deceased donor renal transplantation: a double-blind randomized trial. Br J Anaesth, 2017;119:606-615.

© 2022 McEvoy MD et al. *JAMA Network Open.*

Topic : Patients for whom Normal Saline is of benefit and may be of benefit

Question 13: A 23 year-old woman is admitted after a motor vehicle collision with suspected traumatic brain injury and a Glasgow coma scale score of 6. Resuscitation with what IV solution is the BEST initial choice?

Answers:

- **A.** Albumin 5%
- **B.** Lactated Ringers
- **C.** Plasma-Lyte A
- **D.** Normal Saline

Correct Answer: D

Key Point: In the setting of traumatic brain injury (TBI), normal saline resuscitation with less than 2 L administered initially is associated with lower mortality than with other types of IV fluids.

Rationale: While the recent SALT-ED and SMART trials found that MAKE 30 was increased for patients who received normal saline (NS) as compared to balanced crystalloid solutions, there are still some patients for whom normal saline is indicated, specifically patients with TBI. The SAFE trial demonstrated that saline is superior to albumin in this setting and other trials have shown a possible mortality benefit with normal saline over LR in these patients, with the best outcomes being in patients who received <2L of NS as compared to higher volumes or a different crystalloid (LR). Other patients who may benefit from normal saline include patients with a hypochloremic metabolic alkalosis, such as those with intractable vomiting or nasogastric tube that is actively draining. However, no study to date has demonstrated this.

References:

Rowell SE, et al. The Impact of Pre-Hospital Administration of Lactated Ringer's Solution versus Normal Saline in Patients with Traumatic Brain Injury. J Neurotrauma. 2016;33:1054-9.

Ko A, et al. Limit Crystalloid Resuscitation after Traumatic Brain Injury. Am Surg. 2017;83:1447-1452.

SAFE Study Investigators, et al. Saline or albumin for fluid resuscitation in patients with traumatic brain injury. N Engl J Med. 2007 Aug 30;357(9):874-84.

Gennari FJ, Weise WJ. Acid-base disturbances in gastrointestinal disease. Clin J Am Soc Nephrol. 2008;3:1861-8.

© 2022 McEvoy MD et al. *JAMA Network Open.*

Topic: Effect of balanced crystalloid vs. saline on MAKE 30 in SALT-ED and SMART

Question 14: What is the effect on major adverse kidney events at 30 days after admission (MAKE 30) when hospitalized adult patients receive intravenous normal saline instead of a balanced crystalloid solution?

Answers:

- **A.** Increased rate of MAKE 30 with normal saline
- **B.** Decreased rate of MAKE 30 with normal saline
- **C.** No difference in the rate of MAKE 30 with normal saline

Correct Answer: A

Key Point: Based upon the recently published SALT-ED and SMART trials, administration of balanced crystalloid solutions (Plasmalyte-A, Normosol, Lactated Ringer's) causes **a lower rate of MAKE 30 as compared to administration of normal saline.**

Rationale:
In the SMART and SALT-ED trials, Major Adverse Kidney Events within 30 days (MAKE30) was defined as a composite of death, new renal replacement therapy, or final serum creatinine ≥ 200% of baseline at the earliest of either hospital discharge or 30 days after the index ED visit or admission to the ICU. MAKE30 was a dichotomous outcome. All patients in the trial were eligible for MAKE30. Patients who presented to the ED with end stage renal disease and undergoing chronic renal replacement therapy were eligible for MAKE30 only through death. All other patients were eligible for MAKE30 through all three components—death, new renal replacement therapy, and final serum creatinine ≥ 200% of baseline. Using this metric to assess the effects of balanced crystalloids versus normal saline, the use of balanced crystalloids resulted in a lower rate of MAKE30.

References

Self WH, et al. Balanced Crystalloids versus Saline in Noncritically Ill Adults. N Engl J Med. 2018;378:819-828

Semler MW, et al. Balanced Crystalloids versus Saline in Critically Ill Adults. N Engl J Med. 2018;378:829-839

© 2022 McEvoy MD et al. *JAMA Network Open.*

Topic: Effect of balanced crystalloid vs. saline on MAKE 30 in septic patients in SALT-ED and SMART

Question 15: What is the effect on MAKE 30 (major adverse kidney events at 30 days after admission) of administering normal saline to adult patients with sepsis as compared to a balanced crystalloid solution?

Answers:

- **A.** Increased rate of MAKE 30 with normal saline
- **B.** Decreased rate of MAKE 30 with normal saline
- **C.** No difference in the rate of MAKE 30 between groups

Correct Answer: A

Key Point: Based upon the recently published SMART trial, administration of balanced crystalloid solutions (Plasmalyte-A, Normosol, Lactated Ringer's) causes a lower rate of MAKE 30 as compared to administration of normal saline, especially in patients admitted to the hospital with sepsis.

Rationale: While the exact cause is unknown, administration of balanced crystalloid solutions (Plasmalyte-A, Normosol, Lactated Ringer's) causes a lower rate of MAKE 30 as compared to administration of normal saline, especially in patients admitted to the hospital with sepsis. This was recently demonstrated in the SALT-ED and SMART trials, which together randomized ~28,000 patients to receive either normal saline or a balanced crystalloid solution. The studies did show similar findings to prior research concerning the biochemical alterations associated with normal saline administration – namely, higher chloride values and lower bicarbonate values, the former of which has been strongly associated with renal injury.

References

Self WH, et al. Balanced Crystalloids versus Saline in Noncritically Ill Adults. N Engl J Med. 2018;378:819-828

Semler MW, et al. Balanced Crystalloids versus Saline in Critically Ill Adults. N Engl J Med. 2018;378:829-839

© 2022 McEvoy MD et al. *JAMA Network Open.*

Topic: Risks of hyperchloremia

Question 16: A 58-year-old male is admitted to the ICU with abdominal sepsis. He is given 2500mL (25 mg/kg) of normal saline (0.9% NaCl) for initial volume resuscitation. After the fluid bolus is completed, his labs reveal the following:

Na	142 mEq/L
K	4.9 mEq/L
Cl	113 mEq/L
HCO_3	14 mEq/L
BUN	21 mg/dL
Cr	1.2 mg/dL
Glc	128 mg/dL

Based on the chloride level, the patient is at increased risk for which of the following?

Answers:

- **A.** Pulmonary edema
- **B.** Atrial fibrillation
- **C.** Acute renal dysfunction
- **D.** Acute respiratory distress syndrome

Correct Answer: C

Key Point: Patients administered normal saline during non-cardiac surgery are at increased risk for hyperchloremia (Cl > 110 mEq/L) which is associated with acute kidney injury and death when compared to patients administered isotonic crystalloids such as Plasma-Lyte A or Lactated Ringers. Studies have also reported this association in septic patients.

Rationale: Historically, normal saline (NS) has been the most commonly administered intravenous fluid. Data suggest that intravenous NS may be associated with hyperchloremic metabolic acidosis, acute kidney injury (AKI), and death. Patients who receive NS either after elective non-cardiac surgery or in the setting of sepsis are more likely to develop hyperchloremia (Cl>110 mEq/L). Importantly, the development of hyperchloremia itself has been associated with worse outcomes, including increased AKI and death. The likelihood of developing hyperchloremia is lower when balanced crystalloids (LR or PLA) are administered.

References:

McCluskey SA, et al. Hyperchloremia after noncardiac surgery is independently associated with increased morbidity and mortality: a propensity-matched cohort study. Anesth Analg 2013;117:412-21.

Raghunathan K, et al. Association between the choice of IV crystalloid and in-hospital mortality among critically ill adults with sepsis. Crit Care Med 2014;42:1585-91.

© 2022 McEvoy MD et al. *JAMA Network Open.*

Shaw AD, et al. Association between intravenous chloride load during resuscitation and in-hospital mortality among patients with SIRS. Intensive Care Med. 2014;40:1897-905.

© 2022 McEvoy MD et al. *JAMA Network Open.*

Topic: Effect of IV fluids on serum potassium and acid-base status

Question 17: Administration of which of the following intravenous crystalloid to patients with ESRD increases the likelihood of a patient requiring treatment for hyperkalemia and acidosis?

Answers:

- **A.** Normal Saline
- **B.** Lactated Ringers
- **C.** Plamalyte-A
- **D.** None of the above

Correct Answer: A

Key Point: Administration of normal saline has been shown in multiple high-quality studies to cause more hyperkalemia compared to other crystalloids.

Rationale: Administration of normal saline has been shown in multiple high-quality studies to cause more hyperkalemia compared to other crystalloids. In contrast, there is no evidence that any potassium-containing solution (e.g. LR or PLA/Normosol) causes an increase in potassium, including patients with end-stage renal disease. There is some evidence that normal saline may cause hyperkalemia requiring treatment in patients with end-stage renal disease undergoing transplantation, whereas Lactated Ringer's and Plasma-Lyte do not.

References:

Gonzalez-Castro A, et al. Influence of Proportion of Normal Saline Administered in the Perioperative Period of Renal Transplantation on Kalemia Levels. Transplant Proc. 2018;50:569-571

O'Malley CM, et al. A randomized, double-blind comparison of lactated Ringer's solution and 0.9% NaCl during renal transplantation. Anesth Analg. 2005; 100:1518-24.

Weinburg L. Effects of intraoperative and early postoperative normal saline or Plasma-Lyte 148® on hyperkalaemia in deceased donor renal transplantation: a double-blind randomized trial. Br J Anaesth, 2017;119:606-615.

© 2022 McEvoy MD et al. *JAMA Network Open.*

Topic: Effect of hyperchloremia on renal perfusion and urine output

Question 18: A 20 mL/kg bolus of which intravenous fluid results in the lowest urine output, longest time to micturition after bolus completion, and the most weight gain from fluid retention?

Answers:

- **A.** Albumin 5%
- **B.** Plasma-lyte A
- **C.** Lactated Ringers
- **D.** Normal Saline

Correct Answer: D

Key Point: Two trials in humans have shown that normal saline reduces renal artery flow and renal cortical perfusion while delaying urine output and resulting in more weight gain compared to balanced solutions (Plasma-Lyte A or Lactated Ringer's).

Rationale: Based upon a number of studies showing an association of renal dysfunction after normal saline administration, two prospective randomized, controlled, double-blind crossover trials were performed in humans to evaluate the effects of different crystalloid solutions on renal perfusion and urine output. These trials demonstrated that normal saline caused a reduction in renal artery flow and renal cortical perfusion, and also caused delayed urine output and more weight gain as compared to balanced solutions.

References:

Reid F, et al. (Ab)normal saline and physiological Hartmann's solution: a randomized double-blind crossover study. Clin Sci (Lond). 2003;104:17-24.

Chowdhury AH, et al. A Randomized, Controlled, Double-Blind Crossover Study on the Effects of 2-L Infusions of 0.9% Saline and Plasma-Lyte 148 on Renal Blood Flow Velocity and Renal Cortical Tissue Perfusion in Healthy Volunteers. Ann Surg. 2013;258:1118.

© 2022 McEvoy MD et al. *JAMA Network Open.*

Topic: Effect of balanced crystalloid vs. saline on chloride and acidemia in SALT-ED and SMART

Question 19: Which of the following changes in serum chemistry are MOST likely to occur with administration of Plasma-Lyte A compared to normal saline?

Answers:

- **A.** Increased chloride and increased bicarbonate
- **B.** Increased chloride and decreased bicarbonate
- **C.** Decreased chloride and decreased bicarbonate
- **D.** Decreased chloride and increased bicarbonate

Correct Answer: D

Key Point: Based upon the recently published SALT-ED and SMART trials, administration of normal saline results in higher chloride and lower bicarbonate levels versus Plasma-Lyte A or other balanced crystalloid solutions. The magnitude of the effect is volume/dose related.

Rationale: Numerous studies have shown that the development of hyperchloremia (Cl>110mEq/L) is associated with worse renal outcomes and increased mortality. Based upon these trials, administration of normal saline results in higher chloride and lower bicarbonate levels versus Plasma-Lyte A or other balanced crystalloid solutions. The magnitude of the effect is volume/dose related. Furthermore, the administration of normal saline was associated with increased renal injury and death.

References

Self WH, et al. Balanced Crystalloids versus Saline in Noncritically Ill Adults.
N Engl J Med. 2018;378:819-828

Semler MW, et al. Balanced Crystalloids versus Saline in Critically Ill Adults. N Engl J Med. 2018;378:829-839

McCluskey SA, et al. Hyperchloremia after noncardiac surgery is independently associated with increased morbidity and mortality: a propensity-matched cohort study. Anesth Analg 2013;117:412-21.

Raghunathan K, et al. Association between the choice of IV crystalloid and in-hospital mortality among critically ill adults with sepsis. Crit Care Med 2014;42:1585-91.

Shaw AD, et al. Association between intravenous chloride load during resuscitation and in-hospital mortality among patients with SIRS. Intensive Care Med. 2014;40:1897-905.

© 2022 McEvoy MD et al. *JAMA Network Open.*

Topic: Effect of balanced crystalloid vs. saline on MAKE 30 with PREVIOUS RRT

Question 20: A 49-year-old man with CKD3 who has previously required dialysis is admitted to the ICU with pneumonia. Although IV fluid therapy with Normosol (Plasma-Lyte) is initially ordered, the order is cancelled and the patient is given normal saline instead. What effect on clinical outcomes is MOST likely to result from a change to normal saline?

Answers:

- **A.** Increased risk of acidosis requiring treatment
- **B.** Decreased risk of hyperkalemia requiring treatment
- **C.** Increased risk of mortality
- **D.** Decreased risk of mortality

Correct Answer: D

Key Points: In the SMART trial, patients with a history of renal replacement therapy prior to enrollment in the study had an increased odds ratio of in-hospital mortality when administered normal saline instead of a balanced crystalloid solution.

Rationale: For critically ill patients with a history of renal replacement therapy prior to enrollment in the SMART trial, the odds ratio of in-hospital mortality was greatly reduced (0.61 [95% CI 0.41-0.91, p=0.01]) for patients who received balanced crystalloids rather than normal saline. The exact mechanism of this is not known. However, a number of studies have shown less biochemical perturbations for surgical patients with end-stage renal disease who received a balanced crystalloid rather than normal saline.

References:

Semler MW, et al. Balanced Crystalloids versus Saline in Critically Ill Adults. N Engl J Med. 2018;378:829-839

O'Malley CM, et al. A randomized, double-blind comparison of lactated Ringer's solution and 0.9% NaCl during renal transplantation. Anesth Analg. 2005; 100:1518-24.

Weinburg L. Effects of intraoperative and early postoperative normal saline or Plasma-Lyte 148® on hyperkalaemia in deceased donor renal transplantation: a double-blind randomized trial. Br J Anaesth, 2017;119:606-615.

© 2022 McEvoy MD et al. *JAMA Network Open.*

QuizTime Module:

Appropriate Ordering of Opiod and Non-Opioid Medications:
Function First Opioids Last Program

© 2022 McEvoy MD et al. *JAMA Network Open.*

CATEGORY: acute to chronic opioid use
TOPIC: opioid prescribing
Competencies:
Authors: Allen/Rice

Question 1: Likelihood of Chronic Opioid Use
Question:
A 52-year-old opioid naive woman presents to the emergency department with a migraine headache. Evaluation and management of her condition by a physician who frequently prescribes opioids increases the likelihood of which of the following:

Answers:
A. **Long-term opioid use**
B. Future hospitalization
C. Respiratory failure
D. Cardiac arrest

Key Point:
The approach to treatment of acute pain varies widely amongst healthcare professionals. In opioid-naïve patients, treatment by a clinician who prescribes opioids more frequently than their peers is strongly correlated with subsequent long-term opioid use in patients. Additionally, risk of long-term opioid use increases rapidly with commonly prescribed doses and durations of therapy. Strategies to mitigate risk should be employed with every patient encounter in which opioids are prescribed.

Rationale:
Increasing overuse of opioids in the United States may be driven in part by prescribing habits of healthcare professionals.

In a recent study of almost 400,000 patients (Barnett ML *et al*, NEJM 2017), ED physicians were categorized as being **high-intensity** or **low-intensity opioid prescribers** according to relative quartiles of prescribing rates within their hospital. L**ong-term opioid use** was defined **≥180 days** of opioids supplied in the 12 months after the index ED visit, ***excluding prescriptions within 30 days after the index visit***. Rates of long-term opioid use were compared in patients treated by high or low-intensity prescribers. Overall, patient characteristics and diagnoses treated were similar across all prescribers. There was **>3-fold increase in opioid prescription rates** by the high-intensity prescribers **as compared to** low-intensity (24.1% vs. 7.3% of ED visits, see figure below). Patients treated by high-intensity opioid prescribers had a **30% increased risk of long-term opioid use**.

A similar study (Deyo RA *et al*, JGIM 2017) evaluating **500,000 opioid-naïve patients** demonstrated that both the number of prescriptions in the **1st month of opioid consumption** and **total morphine milligram equivalents (MMEs)**

© 2022 McEvoy MD et al. *JAMA Network Open.*

prescribed were highly correlated with **risk of long-term use**, defined as **≥6 opioid prescriptions** in the subsequent 12 months. After excluding patients with cancer pain and non-cancer chronic pain conditions, compared to the group that only filled 1 prescription in the first month, **those who filled ≥2 were 4 to 10 times as likely to become long-term users.** Additionally, those who were dispensed **>120 morphine milligram equivalents total** (MME; e.g. 120 MME = oxycodone 5mg PO q6h PRN x 4 days) were **2-16 times** as likely to be **long-term opioid users**, with increasing MME dispensed associated with increased risk.

If a prescription is given for opioids for acute non-cancer pain, **the shortest duration and the lowest number of MMEs (by total dose and pill count) possible should be given**. Per latest CDC guidelines, 3 days of opioid therapy is often sufficient and >7 days is rarely needed for acute pain.

© 2022 McEvoy MD et al. *JAMA Network Open.*

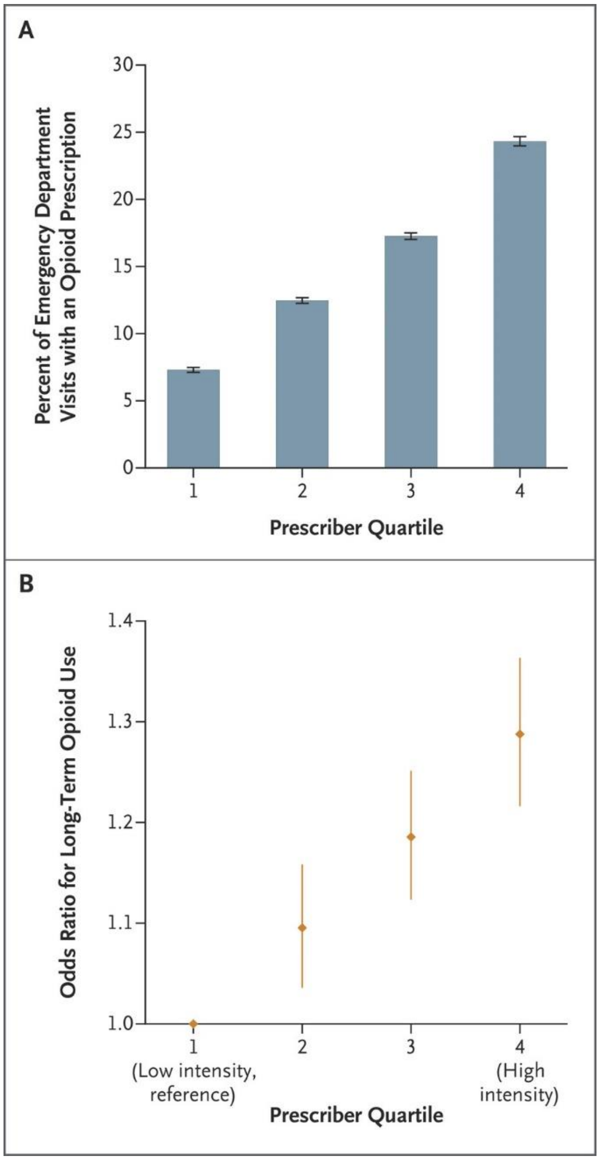

References:

Barnett ML, Olenski AR, Jena AB. Opioid-Prescribing Patterns of Emergency Physicians and Risk of Long-Term Use. *NEJM* 2017;376:663-673.

Deyo RA, Hallvik SE, Hildebran C, Marino M, Dexter E, Irvine JM, O'Kane N, Van Otterloo J, Wright DA, Leichtling G, Millet LM. Association Between Initial Opioid Prescribing Patterns and Subsequent Long-Term Use Among Opioid-Naïve Patients: A Statewide Retrospective Cohort Study. J Gen Intern Med. 2017;32:21-27.

© 2022 McEvoy MD et al. *JAMA Network Open.*

Dowell D, Haegerich TM, Chou R. CDC guideline for prescribing opioids for chronic pain – United States, 2016. JAMA. 2016;315:1624–45.

© 2022 McEvoy MD et al. *JAMA Network Open.*

CATEGORY: opioid risks
TOPIC: opioid risks – dose levels
Competencies: K1
Authors: Hayhurst/Allen

Question 2: Opioid-Related Side Effects
Question:
A 62 year-old F undergoes an emergency exploratory laparotomy for a small bowel obstruction. She has been taking 120mg of sustained release morphine twice a day for 6 months to treat pain from pancreatic cancer. She is currently in 10/10 pain and is receiving IV hydromorphone in the PACU. Compared to an opioid-naïve woman of her same age and weight undergoing the same procedure, which of the following is correct if you attempt to reduce her pain to <3/10?

Answers:
A. **The patient on chronic opioids is at higher risk for respiratory depression**
B. The opioid naïve patient is at higher risk for respiratory depression
C. Neither are likely to experience significant respiratory depression while in a monitored setting
D. These patients would be at equal risk of respiratory depression

Key Point:
There is differential tolerance to opioid effects, meaning the tolerance to some effects is faster and to a greater degree than other effects. **Tolerance to analgesia occurs the fastest**, **tolerance to respiratory depression effects of opioids is slower and limited**, and there is almost no tolerance to the constipation. Thus, attempting to achieve the same level of analgesia (e.g. <3/10 pain score) in an opioid-naïve patient and chronic opioid user puts the chronic opioid user at higher risk of respiratory depression. Thus, it is critical to consider non-opioid options (e.g. neuraxial or regional anesthesia; non-opioid pain medications) to safely achieve perioperative analgesia in these patients.

Rationale:
Common side effects from opioids include nausea, vomiting, ileus, tolerance to analgesia, and respiratory depression. Life-threatening **respiratory depression** from opioid use is likely under-reported. The **risk of respiratory depression increases** for patients that have **OSA, morbid obesity, certain neuromuscular disorders, ASA IV status, and the extremes of age**. The risk also increases when a basal rate is added to a PCA. **Of note, patients on long-term opioids often develop tolerance to the analgesic effects of opioids and several of the opioid-induced side-effects. However, tolerance to the respiratory depressant effects is more limited than the tolerance that develops to analgesic effects of opioids (see Figure).** That is, chronic opioid users can develop tolerance to the analgesic effects and not the respiratory depression,

© 2022 McEvoy MD et al. *JAMA Network Open.*

thus making it possible to have more risk of severe respiratory depression than opioid naïve patients when achieving equivalent levels of analgesia.

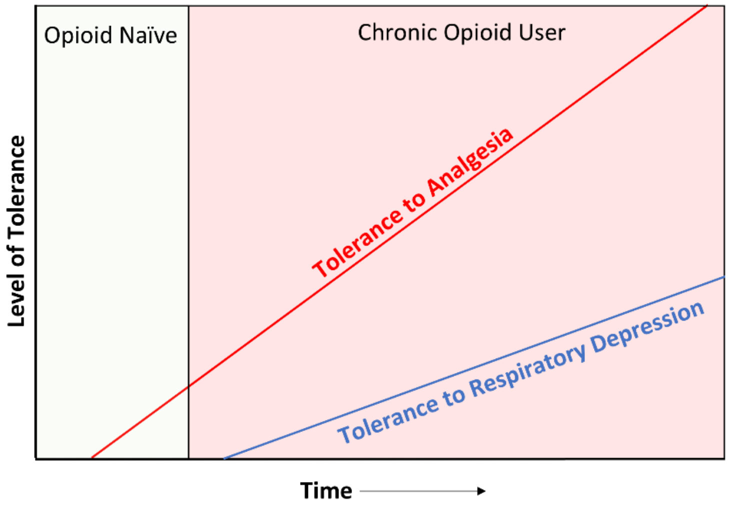

Of additional concern, **constipation** is the most common side effect of opioid administration, affecting **40-95% of patients** treated with opioids and occurring in some even after one dose. **Postoperative ileus** is associated with as little as 2 mg of hydromorphone per day and accounts for prolonged length of stay in many patients. Less common opioid-related side effects include opioid-induced hyperalgesia, sleep disturbance, hallucinations, and immunologic/hormonal dysfunction.

References:

Hayhurst CJ, Durieux ME. Differential Opioid Tolerance and Opioid-induced Hyperalgesia: A Clinical Reality. *Anesthesiology,* 2016, Vol.124, 483-488.
Benyamin R, Trescot AM, Datta S, et al. Opioid complications and side effects. *Pain Physician*, 2008 Mar;11(2 Suppl):S105-20.
Dahan A, Aarts L, Smith TW. Incidence, Reversal, and Prevention of Opioid-induced Respiratory Depression. *Anesthesiology*, 2010 Jan;112(1):226-38.

© 2022 McEvoy MD et al. *JAMA Network Open.*

CATEGORY: Medical treatment of acute pain
TOPIC: Medical treatment – acute opioid; Medical treatment – acute nonopioid
Competencies: C1,C2,C4
Authors: McEvoy/Rice

Question 3: Pain Management Goal – Optimal Analgesia
Question:
Which of the following is the most appropriate goal for managing acute pain in the hospitalized medical or surgical patient?

Answers:

A. Obtaining the lowest pain rating/score possible
B. Using the fewest number of medications possible to obtain pain relief
C. Fastest functional recovery and fewest medication side effects while optimizing patient comfort
D. Using a treatment regimen that results in the fewest calls to the supervising clinician

Key Point:
In attempting to achieve optimal analgesia for patients not on chronic, scheduled opioids at home, the guiding principles include: **1) Opioids should be "added last, prescribed only as needed (PRN), on the shortest period of time possible, and taken off of the analgesia regimen first; 2) Non-opioid analgesic medications should be "added first, ordered as scheduled medications, on longer than any other analgesic, and thus taken off of the regimen last."** We call this the "Function First, Opioids Last" Program.

Rationale:
The goal of any pain management regimen should be to deliver 'optimal analgesia,' which has ben recently defined "as a technique that **optimizes patient comfort and facilitates the fastest functional recovery with the fewest medication side effects**" (see Figure.) This can be for the **inpatient or outpatient setting and for the surgical or medical** patient. Of note, achieving the goal of 'optimal analgesia' **may not correspond with the lowest pain score/rating possible**. Overall, the combination of analgesic techniques employed is not important as long as it is effective in delivering this goal of 'optimal analgesia.' However, **guiding principles** include the following: **1) Opioids should be "added last, prescribed only as needed, on the shortest period of time possible, and taken off of the regimen first; 2) Non-opioids should be "added first, given as scheduled medications, on longer than any other analgesic, and thus taken off of the regimen last."** This includes prescriptions for the inpatient and post-discharge/outpatient settings. Of particular importance, it should be remembered that scheduled NSAIDs + acetaminophen typically result in 30-40% reduction in opioid requirements for acute pain; this goes up even further if gabapentinoids are used.

© 2022 McEvoy MD et al. *JAMA Network Open.*

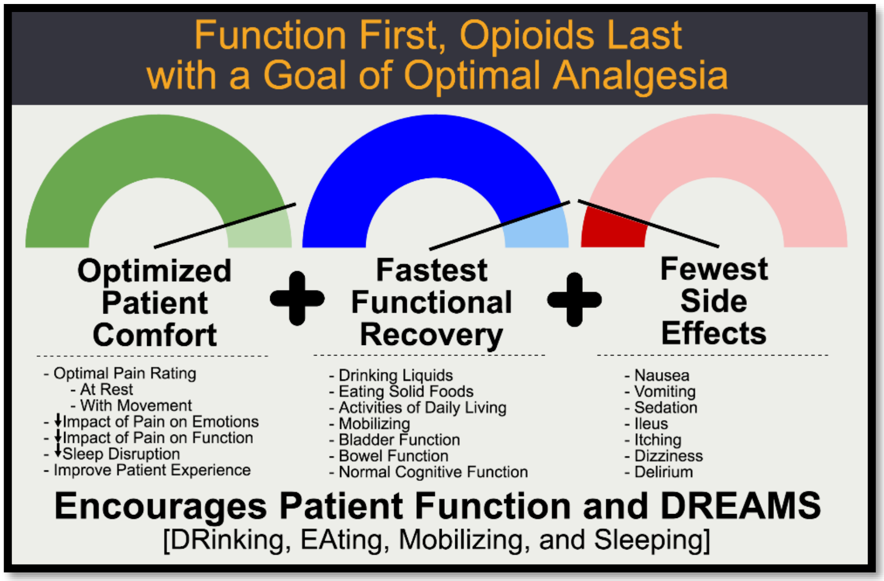

References:

McEvoy MD, et al. American Society for Enhanced Recovery (ASER) and Perioperative Quality Initiative (POQI) joint consensus statement on optimal analgesia within an enhanced recovery pathway for colorectal surgery: Part 1 - From the preoperative period to PACU. *Peioperative Med*, 2017

Scott MJ, et al. American Society for Enhanced Recovery (ASER) and Perioperative Quality Initiative (POQI) joint consensus statement on optimal analgesia within an enhanced recovery pathway for colorectal surgery: Part 2 - From PACU to the Transition Home. *Peioperative Med*, 2017

© 2022 McEvoy MD et al. *JAMA Network Open.*

CATEGORY: Opioid risk
TOPIC: Opioid risk – dose levels
Competencies: K1
Authors: Edwards/Rice

Question 4: Opioids – Dose-related Risk of Death
Question: A 30-year-old, healthy narcotic-naïve woman is discharged following a cholecystectomy with a prescription for oxycodone 10 mg PO q4h PRN x 14 days. What is her relative risk of overdose compared to a patient taking 15 morphine milligram equivalents (MME)/day?

Answers:

A. approximately half
B. approximately equal
C. **approximately 2-4 times more**
D. approximately 10 times more

Key Point:
Risk of overdose events is dose-related and greatly increased risk occurs in ranges that are commonly prescribed. Patients taking 50-99 MME/day at **2-4 times the risk for overdose** compared to <20 MME/day.

Rationale:
Risk of opioid overdose is dose related and is quantified according to the amount of morphine milligram equivalents (MME)/day that a person is taking. Oxycodone is 1.5 times as potent as morphine. If the patient uses oxycodone as prescribed, then she will be taking up to 60 mg oxycodone/day = 90 MME. Opioid doses of 50–99 MME/day are at **2-4 times the risk for overdose** compared to <20 MME/day.

One large fair-quality retrospective cohort study found that recent opioid use was associated with increased risk for any overdose events and serious overdose events versus nonuse. It also found higher doses associated with increased risk. Relative to <20 MME/day, the adjusted hazard ratio (HR) for any overdose event (consisting of mostly nonfatal overdose) was 1.44 for 20 to 49 MME/day, 3.73 for 50–99 MME/day, and 8.87 for ≥100 MME/day. A similar pattern was observed for serious overdose.

A good-quality population-based, nested case-control study also found a dose-dependent association with risk for overdose death. Relative to <20 MME/day, the adjusted odds ratio (OR) was 1.32 for 20–49 MME/day, 1.92 for 50–99 MME/day, 2.04 for 100–199 MME/day, and 2.88 for ≥200 MME/day.

References:

© 2022 McEvoy MD et al. *JAMA Network Open.*

Dunn KM, Saunders KW, Rutter CM, et al. Opioid prescriptions for chronic pain and overdose: a cohort study. Ann Intern Med 2010;152:85–92. PubMed

Gomes T, Mamdani MM, Dhalla IA, Paterson JM, Juurlink DN. Opioid dose and drug-related mortality in patients with nonmalignant pain. Arch Intern Med 2011;171:686–91.

https://www.cdc.gov/drugoverdose/pdf/calculating_total_daily_dose-a.pdf

© 2022 McEvoy MD et al. *JAMA Network Open.*

Question 5: **Opioids and CSMD Prescribing Laws [EDWARDS/ROBERTSON]**

Question: A patient is admitted for repair of a traumatic femoral fracture. He takes morphine at home for chronic pain. While at the hospital he is instead prescribed oxycodone at an increased dose to treat acute pain and chronic pain. Which of the following statements regarding Tennessee Laws for opioid prescribing is CORRECT?

Answers:

A. A prescriber must check the Tennessee Controlled Substance Database (CSMD) prior to prescribing an opioid to any patient at the time of hospital discharge.
B. A prescriber does not need to check the CSMD before prescribing opioids at discharge if the patient was already on opioids prior to hospital admission.
C. A prescriber must check the CSMD before any new opioid prescription that exceeds a 7-day supply.
D. A prescriber must check the CSMD prior to prescribing opioids to a patient currently receiving hospice care.

Key Point:

In 2016, the Tennessee House Bill 2571 was passed into law requiring all prescribers (or their designated healthcare practitioner extenders) to check the CSMD prior to prescribing or dispensing controlled substances at the beginning of a new episode of treatment, except for a non-refillable prescription as part of a treatment for a surgical procedure that occurred in a licensed healthcare facility and **is limited to a seven-day supply**

Rationale:

In 2016 Tennessee House Bill 2571 was passed into law requiring all prescribers (or their designated healthcare practitioner's extenders) to check the CSMD prior to prescribing or dispensing controlled substances at the beginning of a new episode of treatment. A new episode of treatment means a prescription that has not been prescribed or dispensed by that prescriber or dispensing practice within the previous twelve months. Controlled substances include all opioids and benzodiazepines. Prescribers are not required to check the CSMD if one or more of the following are met:

A. The controlled substance is prescribed or dispensed for a patient who is currently receiving hospice care;
B. The controlled substance is prescribed or dispensed to a patient as a non-refillable prescription as part of a treatment for a surgical procedure that occurred in a licensed healthcare facility and **is limited to a seven-day supply**; or
C. The controlled substance is prescribed for administration directly to a patient during the course of inpatient or residential treatment in a hospital or nursing home.

http://www.capitol.tn.gov/Bills/109/Bill/HB2571.pdf

Question 6: Use of Multimodal First [EDWARDS/ROBERSTON]

© 2022 McEvoy MD et al. *JAMA Network Open.*

Question: An 83-year-old male presents with severe LLQ abdominal pain consistent with diverticulosis diagnosed by CT scan. The patient's pain remains moderate (5-7/10) despite appropriate antibiotic therapy and management with oxycodone 5-10 mg PO q4 hrs PRN for pain. Labs reveal an elevated white count but no other abnormalities on CBC or BMP. What is the most appropriate next step in pain management for this patient?

Answers:

A. **Addition of scheduled acetaminophen and gabapentin**
B. Initiation of extended-release oxycodone
C. Initiation of hydromorphone IV PRN
D. Increased frequency of oral oxycodone dosing

Key point:
Patients with poorly controlled pain should have multimodal non-narcotic medications initiated BEFORE escalation of opioid therapy.

Rationale:
Ordering oxycodone 10 mg q4 hrs PRN can result in administration of up to 60 mg PO oxycodone in a 24 hour period, roughly equivalent to 90 mg morphine milligram equivalent (MME) per day. Risk of overdose death increases above 20 MME and is almost 10 time higher at >100 MME/day. Elderly patients require lower doses of opioids and are at higher risk for respiratory depression than younger patients.

Alternatively, use of scheduled non-opioid multimodal analgesics such as acetaminophen, NSAIDs, and gabapentin have been shown to decrease opioid requirements for patients with acute pain by 30-40%. Their initiation may allow better pain control and decreased opioid requirements. This approach is preferred over increasing short acting opioids or adding IV opioids to the regimen.

In the setting of acute pain, IV opioids should be reserved for scenarios in which the patient is unable to tolerate oral intake, requires strict NPO status, or for rescue analgesia if patient has severe pain. Long-acting opioids should be avoided and only reserved for treating chronic pain, if at all.

In summary, when treating medical or surgical patients with acute pain the **guiding principles** include: **1) Opioids should be "added last, prescribed only as needed, should be on the shortest period of time possible, and taken off of the regimen first; 2) Non-opioids should be "added first, given as scheduled medications, on longer than any other analgesic, and thus taken off of the regimen last."** This includes prescriptions for the inpatient and post-discharge/outpatient settings.

References:

© 2022 McEvoy MD et al. *JAMA Network Open.*

Macintyre PE, Jarvis DA. Age is the best predictor of postoperative morphine requirements. PAIN. 1996;64(2):357-364.
Cepeda M. Side effects of opioids during short-term administration: effect of age, gender, and race. Clinical Pharmacology & Therapeutics. 2003;74(2):102-112. doi:10.1016/S0009-9236(03)00152-8.
https://www.tn.gov/assets/entities/health/attachments/ChronicPainGuidelines.pdf
Kong VKF, Irwin MG. Gabapentin: a multimodal perioperative drug? Br J Anaesth. 2007;99(6):775-786. doi:10.1093/bja/aem316.

Question 7: Non-opioid analgesics [McEvoy/Allen]

Question: Using appropriate doses of a combination of SCHEDULED acetaminophen and ibuprofen for acute pain results in approximately what degree of opioid reduction?

Answers:

A. 20%
B. 40%
C. 60%
D. 80%

Key Point:
Use of a combination of scheduled non-opioid multimodal analgesics such as acetaminophen and NSAIDs have been shown to decrease opioid requirements for patients with acute pain by 30-50%.

Rationale:
Use of a combination of scheduled non-opioid multimodal analgesics such as acetaminophen, NSAIDs, and gabapentin have been shown to decrease opioid requirements for patients with acute pain by 30-50%. Their initiation may allow overall better pain control than with opioids alone. As with any medication, appropriate dosage should be administered, which is a maximum of 10mg/kg for ibuprofen and 15 mg/kg for acetaminophen, assuming no dosage adjustments are needed for liver or kidney disease. For gabapentin, starting at 300 mg TID is reasonable for patients <70 yo, and 100 mg TID for those >70 yo. However, as gabapentin clearly has a dose-response effect, rapid escalation in the inpatient setting to the highest does without side effects (e.g. sedation, dizziness) is appropriate. Alpha-2 agonists have also shown benefit, but caution should be taken with patients at risk of hypotension and/or bradycardia.

In summary, ibuprofen (or other NSAID) and acetaminophen should be scheduled at the maximum appropriate dose for patient weight, whereas gabapentin can be started at a reasonable dose and quickly titrated up to the optimal dose, which is the highest dose without side effects. Additionally, when treating medical or surgical patients with acute or chronic pain, the **guiding principles** for use of multimodal medications include that they **should be a) added first, b) given as scheduled medications, c) on longer than any other analgesic, and d) taken off of the pain regimen last.**

These principles include orders/prescriptions for the inpatient and post-discharge/outpatient settings.

References:

© 2022 McEvoy MD et al. *JAMA Network Open.*

Sutton CD, et al. Optimal Pain Management After Cesarean Delivery. Anesthesiol Clin. 2017;35:107-124 (https://www-ncbi-nlm-nih-gov.proxy.library.vanderbilt.edu/pubmed/28131114)

Li JMW. Pain management in the hospitalized patient. Med Clin North Am. 2008;92:371-385 (https://www-ncbi-nlm-nih-gov.proxy.library.vanderbilt.edu/pubmed/18298984)

Scott MJ, et al. American Society for Enhanced Recovery (ASER) and Perioperative Quality Initiative (POQI) joint consensus statement on optimal analgesia within an enhanced recovery pathway for colorectal surgery: Part 2 - From PACU to the Transition Home. *Peioperative Med*, 2017

© 2022 McEvoy MD et al. *JAMA Network Open.*

Question 8: NSAIDS and safe dose range [ROBERTSON/FLEMING]
Question:
In which of the following clinical scenarios is IV ketorolac an appropriate choice?
Answers:

A. 55 yo M admitted with right upper quadrant abdominal pain and dehydration from 2 days of vomiting
B. 75 yo F POD0 after arteriovenous fistula creation for hemodialysis; she cannot tolerate opioids due to nausea
C. 36 yo M admitted with a small subarachnoid hemorrhage 5 days ago; no neurological deficits and is complaining of severe headache.
D. **41 yo obese F POD1 after hysterectomy who has incisional pain**

Key point: Ketorolac is an NSAID that is indicated to treat moderate to severe pain, and is widely used after surgery. Its use in multimodal analgesia regimens provides significant opioid reduction and reduced opioid-related side effects. Ketorolac should be avoided in patients with cerebrovascular bleeding, active peptic ulcer disease/GI bleeding, or those in renal failure or risk of renal failure.

Rationale:
Ketorolac is an NSAID with high COX-1 selectivity that is indicated to treat moderate to severe pain, and is widely used after surgery. It is available in IV, IM, and PO forms. Prior studies have found 30mg of IM ketorolac to have similar analgesic efficacy as 12mg of IV Morphine.[1] Its use in multimodal analgesia regimens provides significant opioid reduction and reduced side effects.[2]

Table 2. Adult Dosing and Administration of Parenteral NSAIDs Used for Pain Control

Generic	Formulation	Dosing	Max Daily Dose
Ketorolac tromethamine	IM[a]	*Single dose:* Age <65 y: one 60 mg dose; age ≥65 y, renally impaired and/or BW <50 kg: one 30 mg dose<br>*Multiple dose:* Age <65 y: 30 mg every 6 h	If receiving multiple-dose treatment and age <65 y: 120 mg<br>If receiving multiple-dose treatment and age ≥65 y: 60 mg
Ketorolac tromethamine	IV[b]	*Single dose:* Age <65 y: one 30 mg dose; age ≥65 y, renally impaired and/or BW <50 kg: one 15 mg dose<br>*Multiple dose:* Age <65 y: 15 mg every 6 h	If receiving multiple-dose treatment and age <65 y: 120 mg<br>If receiving multiple-dose treatment and age ≥65 y: 60 mg
Ibuprofen (IVib)	IV[c]	400-800 mg every 6 h as needed	3,200 mg

BW: body weight; max: maximum. [a] Inject slowly and deeply in the muscle. [b] Bolus push over at least 15 seconds. [c] Infuse over at least 30 minutes. Source: References 8, 9.

Due to its nature as an NSAID however, there are several side effects and contraindications to use, especially in the post surgical population. It is only approved for 5 days of total use, due to increased risk of GI bleeding. Dose reduction should be considered when prescribing to those over 65 (see table) and the oral form is not approved for children. Ketorolac is contraindicated in renal failure or risk of renal failure, in patients with active peptic ulcer disease or recent GI bleed, suspected or confirmed cerebrovascular bleeding or concurrently with other NSAIDs. There is controversy surrounding whether ketorolac inhibits bone healing after orthopedic procedures. Additionally, ketorolac is contraindicated after CABG due to a black box warning surrounding all NSAIDs. Some reports suggest safety, and even better outcomes, with its use.

In many patients, ketorolac is a very effective non-opioid analgesic for use in medical or surgical patients with moderate to severe pain.

References:

© 2022 McEvoy MD et al. *JAMA Network Open.*

1 Yee JP, Koshiver JE, Allbon C, Brown CR. Comparison of intramuscular ketorolac tromethamine and morphine sulfate for analgesia of pain after major surgery. *Pharmacotherapy* 1986; 6: 253-61
2 Cepeda MS, Carr DB, Miranda N, Diaz A, Silva C, Morales O. Comparison of morphine, ketorolac, and their combination for postoperative pain: results from a large, randomized, double-blind trial. *Anesthesiology* 2005; 103: 1225-32

Oliveri L[1], Jerzewski K[1], Kulik A[2]. Black box warning: is ketorolac safe for use after cardiac surgery? J Cardiothorac Vasc Anesth. 2014;28:274-9.

Question 9: Multimodal – Use as Scheduled [McEvoy/Robertson]

Question: An opioid-naïve 28 yo F who is G2P1 undergoes cesarean delivery. Which of the following represents the best initial plan for postoperative analgesia?

Answers:

A. intravenous patient-controlled analgesia (PCA) for 24 hours
B. oral opioids, acetaminophen, and ibuprofen PRN
C. scheduled acetaminophen and NSAIDs with oral opioids PRN
D. scheduled acetaminophen, NSAIDs, and gabapentin with oral opioids PRN

Key Point: Multimodal non-opioid analgesics, particularly acetaminophen and ibuprofen, have been shown to reduce opioid consumption by 30-40% when used in a scheduled regimen. These medications should be scheduled prior to initiating any opioid therapy and opioids should initially be ordered PRN. Gabapentinoids can be used safely in almost all patients, including the peripartum period. But, recommendations are to add if initial first-line scheduled use of multimodal analgesics is not sufficient.

Rationale:
Use of scheduled non-opioid multimodal analgesics such as acetaminophen, NSAIDs (e.g. ibuprofen or ketorolac), and gabapentin have been shown to decrease opioid requirements when used at appropriate doses. As with any medication, appropriate dosage should be administered, which is a maximum of 10mg/kg for ibuprofen and 15 mg/kg for acetaminophen, assuming no dosage adjustments are needed for liver or kidney disease. For most women, this will be ibuprofen 600-800mg PO q6h (ketorolac 15-30 mg IV q6h) and acetaminophen 650-1000 mg PO q6h. For gabapentin, in the peripartum period it is reasonable to start with an initial dose of 600 mg PO and then order 300 mg TID. However, as gabapentin clearly has a dose-response effect, rapid escalation in the inpatient setting to the highest does without side effects (e.g. sedation, dizziness) is appropriate.

In summary, ibuprofen (or other NSAID) and acetaminophen should be scheduled at the maximum appropriate dose for patient weight, whereas

© 2022 McEvoy MD et al. *JAMA Network Open.*

gabapentin can be started at a reasonable dose and quickly titrated up to the optimal dose if the initial multimodal agents are not sufficient (see Table).

Table 2
Suggested analgesic protocol for post–cesarean delivery in-hospital pain management

Setting	Drug	Dose and Route	Prescribing Information
Standard care[a] (prescribed at time of surgery)	Neuraxial morphine	Preferred: intrathecal morphine 100–150 µg or epidural morphine 2–3 mg after delivery	With intrathecal hyperbaric bupivacaine 12 mg and fentanyl 15 µg<br>With epidural 2% lidocaine 15–25 mL (±bicarbonate and epinephrine) ± fentanyl 50–100 µg
	NSAIDs	Ibuprofen 600 mg PO (or ketorolac 15 mg IV if NPO)	Every 6 h (scheduled) for 48–72 h after cesarean delivery
	Acetaminophen	Acetaminophen 650 mg PO (or IV if NPO)	Every 6 h (scheduled) for 48–72 h after cesarean delivery
	Oral opioids	Oxycodone 5–10 mg PO	As needed for breakthrough pain:<br>VNPS ≤4/10: 5 mg<br>VNPS >4/10: 10 mg
Ongoing or severe postoperative pain[b]	IV opioids	IV morphine, fentanyl, or hydromorphone	Intermittent IV boluses or IV patient-controlled analgesia
	Regional anesthesia	Bilateral TAP block	0.25% ropivacaine 20–25 mL per side
	Oral adjuvants	Gabapentin	600 mg PO rescue dose (300 mg PO every 8 h for ongoing severe pain)
		Dexamethasone	4–8 mg PO

When treating medical or surgical patients with acute or chronic pain, the **guiding principles** for use of multimodal medications include that they **should be a) added first, b) given as scheduled medications, c) on longer than any other analgesic, and d) taken off of the pain regimen last.** These principles include orders/prescriptions for the inpatient and post-discharge/outpatient settings.

References:

Sutton CD, et al. Optimal Pain Management After Cesarean Delivery. Anesthesiol Clin. 2017;35:107-124 (https://www-ncbi-nlm-nih-gov.proxy.library.vanderbilt.edu/pubmed/28131114)

© 2022 McEvoy MD et al. *JAMA Network Open.*

Question 10: APAP and safe dose question [ROBERTSON/FLEMING]

Question:
A 32 yo male was discharged from the hospital after being treated for cellulitis. Which of the following analgesic regimens is most likely to be associated with a drug-related complication after discharge?

Answers:

A. Percocet 5/325mg, 1-2 tabs PO Q6h PRN and ibuprofen 600 mg PO q6h
B. Percocet 5/325mg, 1 tab PO Q6h PRN and acetaminophen 1000mg PO Q6h
C. Acetaminophen 1000 mg PO Q6h and ibuprofen 600mg PO q6h
D. Percodan 10mg, 1 tab PO Q6h PRN and acetaminophen 1000mg PO Q6

Key Point: In order to avoid potential overdose, patients should be clearly educated about combining over the counter pain (OTC) medications with medications that contain combined formulations, such as Percocet (oxycodone and acetaminophen).

Rationale:
Acetaminophen is one of the most widely used drugs in the world. Overdose can lead to serious liver injury and death. While exceeding the recommended limit of 4g per 24 hours of acetaminophen appears to be relatively rare in the outpatient setting (3% of usage days), it is more likely to occur when patients combine prescribed medications containing acetaminophen and OTC medications with acetaminophen. This could also occur with combinations containing NSAIDs, such as percodan (oxycodone plus aspirin), that are taken with other NSAIDs. Accordingly, in order to avoid potential overdose, patients should be clearly educated about the risks of combining over the counter pain medications with medications that contain combined formulations, such as Percocet (oxycodone and acetaminophen).

Of note, in a prospective, randomized, double-blind, placebo-controlled study of adults with post-surgical pain, 1000mg of acetaminophen was superior to 650mg dose for pain control.[2] The recommended dosing schedule for adults and children over the age of 12 is 650mg every 4 to 6 hours or 1000mg every 6 hours, not to exceed 4 grams in a 24-hour period. Thus, in order to maximize the effects of acetaminophen, prescribing the patient a scheduled regimen of acetaminophen along with an opioid PRN for rescue analgesia may be safer and of greater benefit than using a combination medication such as Percocet. Such an approach would follow the pain management principles outlined in the Function First, Opioids Last program: **1) Opioids should be added last, prescribed only as needed, on for the shortest period of time possible, and removed from the regimen first; 2) Non-opioids should be added first, given as scheduled medications, on for a longer timeframe than any other analgesic, and thus removed from the regimen last.**

© 2022 McEvoy MD et al. *JAMA Network Open.*

References:
1. Shiffman S, Rohay JM, Battista D, et al. Patterns of acetaminophen medication use associated with exceeding the recommended maximum daily dose. *Pharmacoepidemiol Drug Saf* 2015; **24**: 915-21

2. Qi DS, May LG, Zimmerman B, et al. A randomized, double-blind, placebo-controlled study of acetaminophen 1000 mg versus acetaminophen 650 mg for the treatment of postsurgical dental pain. *Clin Ther* 2012; **34**: 2247-58 e3

3. McEvoy MD, et al. American Society for Enhanced Recovery (ASER) and Perioperative Quality Initiative (POQI) joint consensus statement on optimal analgesia within an enhanced recovery pathway for colorectal surgery: Part 1 - From preoperative period to PACU. *Perioperative Med*, 2017

Question 11: Gabapentinoid Dosing [WALTERS/FLEMING]
A 65 y/o male who is admitted for acute on chronic CHF exacerbation also complains that his feet are "numb" and it feels like he is "walking on pins and needles." You diagnose diabetic peripheral neuropathy and decide to prescribe gabapentin. What are the dosing considerations for gabapentin and pregabalin for this patient?

Answers:
A. Gabapentin and pregabalin are not appropriate for patients with CKD who suffer from neuropathic pain, even with dosing adjustments.
B. An appropriate starting dose in most patients is gabapentin 300mg PO TID.
C. Gabapentin has a higher bioavailability after oral administration than pregabalin.
D. The dose of gabapentin and pregabalin should only be increased once per month due to safety concerns.

Key Point: Based on prior research, a safe and effective starting dose in most patients is gabapentin 300mg PO TID or pregabalin 75mg PO BID. Doses can be uptitrated daily in the inpatient setting if monitoring for side effects (e.g. sedation/dizziness) and weekly in the outpatient setting.

Rationale: Gabapentin and pregabalin function as calcium channel antagonists, blocking the release of neurotransmitters. These medications are indicated in the treatment of neuropathic pain, including diabetic peripheral neuropathy, postherpetic neuralgia, central neuropathic pain and fibromyalgia. They have also been shown to be beneficial analgesic adjuncts in a number of postoperative settings. Despite the same mechanism of action, gabapentin and pregabalin have differing pharmacokinetic profiles, which alter effective dosing regimens. Following oral administration, gabapentin has zero-order absorption and its bioavailability varies from approximately 60% at a total daily dose of 900mg to only 33% at doses up to 3600mg per day. Pregabalin follows a linear absorption pattern with a bioavailability of over 90% at all doses. Both gabapentin and

© 2022 McEvoy MD et al. *JAMA Network Open.*

pregabalin have a half-life of 6 hours and **primarily undergo renal excretion**. This indicates that increasing doses of pregabalin will likely produce more predictable changes in pain improvement.

Based on prior research, a safe and effective starting dose in most patients is gabapentin 300mg PO TID. Doses can be uptitrated daily in the inpatient setting if monitoring for side effects (e.g. sedation/dizziness) and weekly in the outpatient setting. Serpell conducted a gabapentin dosing study with an initial total daily dose of 300mg, divided into three doses, and titrated up to a **total daily dose** of 900mg per day over the course of 3 days. There was minimal side effects for most patients at this dosing level. For patients who reported an improvement in pain at 900mg per day, further titration was increased over the course of 5 weeks to 2400mg per day. Patients receiving gabapentin showed a statistically significant improvement in pain scores compared to placebo with only mild to moderate side effects reported. The maximum recommended dosing for gabapentin is currently 3600mg per day. Additionally, *Freynhagen et al.* investigated pregabalin dosing and determined that doses ranging from **150-600mg per day provided statistically significant improvement in pain scores**. Patients were started at 150mg per day divided into two doses and titrated weekly based on response and tolerability. The study showed a low dropout rate due to side effects. The current maximum dose of pregabalin is 600mg per day. **Due to primary renal excretion with both gabapentin and pregabalin, dosing should be reduced based on creatinine clearance with additional doses given after hemodialysis**. When ordering as an inpatient at VUMC, q8h is preferred (i.e. 0600, 1400, 2200) due to uneven dosing intervals with TID (i.e. 1000, 1400, 2200).

References:

1. Serpell MG. Gabapentin in neuropathic pain syndromes: a randomised, double blind, placebo-controlled trial. *Pain* 2002; 99(3): 557-566.
2. Freynhagen R, Strojek K, Griesing T, Whalen E, Balkenohl M. Efficacy of pregabalin in neuropathic pain evaluated in a 12-week, randoised, double blind, multicenter, placebo-controlled trial of flexible-and fixed dose regimens. *Pain* 2005; 115(3): 254-63.
3. Moore RA, Wiffen PJ, Derry S, Toelle T, Rice A. Gabapentin for chronic neuropathic pain and fibromyalgia in adults. *Cochrane Database Syst Rev* 2014; 27(4).
4. Moore RA, Straube S, Wiffen PJ, Derry S, McQuay HJ. Pregabalin for acute and chronic pain in adults. *Cochrane Database Syst Rev* 2009; 8(3).

© 2022 McEvoy MD et al. *JAMA Network Open.*

Question 12: Gabapentinoids Side Effects [WALTERS/FLEMING]
Question 12: Gabapentinoids Side Effects
Question: A 70 y/o male with a h/o smoking and lumbar stenosis with radiculopathy is POD 1 s/p L3-L5 laminectomy and fusion. Postoperatively he is started on acetaminophen 1000mg PO q8h, gabapentin 600mg q8h, and hydromorphone 0.5mg IV q4h PRN. On morning rounds, the patient appears somnolent. Which statement is TRUE regarding side effects of pregabalin and gabapentin therapy?

Answers:

A. **Dizziness and somnolence are the most common side effects with both gabapentin and pregabalin**
B. Respiratory depression is common with the use of gabapentin or pregabalin.
C. Significant hyponatremia (sodium <130mEq/L) develops in approximately 5% of patients taking gabapentin
D. Side effects occur in a dose-dependent phenomenon for patients administered pregabalin.

Key Point: Systematic reviews of the literature concerning gabapentinoids use in medical and surgical patients support substantial analgesic benefit (30-50% reduction in pain scores) with relatively common minor side effects, but no serious adverse events (e.g. respiratory depression) when compared to placebo.

Rationale: A Cochrane review involving pregabalin demonstrated benefit at doses ranging from **300-600mg per day** for diabetic peripheral neuropathy (DPN), postherpetic neuralgia (PHN), central neuropathic pain (CNP) and fibromyalgia. Lower dosing regimens were typically required for PHN and DPN compared to fibromyalgia in order to reach a **30-50% reduction in pain scores**. At a dose of 600mg/day, dizziness was the most common side effect, occurring in 27-46% of patients, with rates of somnolence ranging from 15-25%. Patient withdrawal rates due to side effects ranged were approximately 25%. The pregabalin study also stated that side effects were surprisingly *not dose-dependent*. A similar systematic review involving **gabapentin** reported an average dosing regimen of **1200mg per day** with a significant portion of the studies focusing on the treatment of neuropathic pain conditions. The NNT to provide a **50% pain reduction** was 5.9 for DPN and 8.0 for PHN. An average of 11% of patients discontinued treatment due to side effects. Dizziness was the most common side effect occurring in 19% of patients with somnolence reported in 14% of patients. Additional side effects reported at much lower rates in this review included peripheral edema and gait disturbance. Both systematic reviews reported rates of serious adverse events that were no more common than placebo.

Similar findings have been reported in surgical patients in a wide variety of surgeries (e.g. abdominal, orthopedic). The dose-response relationship is also very clear in these populations with substantial benefit starting with single preoperative doses of ≥600 mg PO or with daily dosing regimens as noted

© 2022 McEvoy MD et al. *JAMA Network Open.*

above. Of note, approximately 1% of patients taking gabapentin will develop significant hyponatremia (<130mEq/L). This typically resolves within 24 hours of stopping therapy.

In summary, systematic reviews of the literature concerning gabapentinoids use in medical and surgical patients support substantial analgesic benefit (30-50% reduction in pain scores) at higher dosing regimens with relatively common minor side effects, but no serious adverse events (e.g. respiratory depression) when compared to placebo.

References:

1. Moore RA, et al. *Cochrane Database Syst Rev* 2014; 27(4).
2. Moore RA, et al. *Cochrane Database Syst Rev* 2009; 8(3).
3. Tiippana EM, et al. *Anesth & Analgesia* 2007; 104:1545-56.

© 2022 McEvoy MD et al. *JAMA Network Open.*

Question 13: [ALLEN/FLEMING]
Question: A 27 yo female undergoes surgical treatment for a dental abscess. She receives a prescription for oxycodone 5 mg PO q6h PRN pain (90 tablets) from the oral surgeon on discharge. After 3 days of taking 2 to 4 oxycodone tablets per day, her pain is well-controlled with acetaminophen. What strategy should be used to discontinue oxycodone?

Answers:

A. Taper dosage to 2.5 mg oxycodone four times a day for three days, then discontinue
B. Taper frequency to 5 mg oxycodone twice a day for three days, then discontinue
C. Stop oxycodone, take clonidine 0.1 mg PO twice a day for withdrawal symptoms
D. Stop oxycodone, no tapering necessary

Key point:
After low-dose, short-duration therapy, opioids may be stopped without tapering. Additionally, per latest evidence, prescriptions should be planned to be as short as possible to prevent both a lower likelihood of patients becoming chronic opioid users and the risk of having unused pills in the community.

Rationale:
Per latest CDC guidelines, 3 days of opioid therapy is often sufficient and >7 days is rarely needed for acute pain. If these recommendations are followed, opioid withdrawal is unlikely. Stopping opioids without tapering is acceptable under these circumstances. The incidence of withdrawal increases with longer durations and higher dosages of opioids. Unlike benzodiazepine or alcohol withdrawal, opioid withdrawal is not life-threatening.

In attempting to achieve optimal analgesia for opioid-naïve patients in acute pain, the guiding principles include: **1) Opioids should be "added last, prescribed only as needed (PRN), on the shortest period of time possible, and taken off of the analgesia regimen first; 2) Non-opioid analgesic medications should be "added first, ordered as scheduled medications, on longer than any other analgesic, and thus taken off of the regimen last."** We call this the **"Function First, Opioids Last" Program**.

For this patient, that would include scheduling acetaminophen and ibuprofen (or other NSAID) while using opioids only as needed and giving as few pills as possible. This patient likely would have had adequate analgesia if the same prescription was given with only 10 pills total.

References:
https://www.tn.gov/assets/entities/health/attachments/ChronicPainGuidelines.pdf

https://www.cdc.gov/mmwr/volumes/66/wr/mm6610a1.htm?s_cid=mm6610a1_e

© 2022 McEvoy MD et al. *JAMA Network Open.*

Question 14: Duration of prescription - Frequency and number of pills given [McEvoy/Fleming]

Question: When prescribing opioids to opioid-naïve medical patients without cancer, the shortest duration of initial opioid prescription associated with a *doubling* of the probability of continued opioid use after 1 year is:

Answers:

A. **5 days**
B. 10 days
C. 14 days
D. 30 days

Key Point: In a recent report from the CDC**,** the likelihood of chronic opioid use (defined as opioid use 1 year after the initial opioid prescription) increased with *each additional day* of medication supplied, starting with the 3rd day. Probability of chronic use was doubled at the 5th day (see figure).

Rationale: Based on the CDC Guideline for Prescribing Opioids for Chronic Pain, literature supporting long-term opioid therapy for pain is limited; in fact, research suggests an increased risk for harms with long-term opioid use. Early opioid prescribing patterns for opioid-naïve patients have been found to be associated with the likelihood of long-term use. A recent report noted that in a representative sample of ~1.3 million opioid naïve, cancer-free adults who received at least a 1-day prescription for opioid pain relievers, the likelihood of chronic opioid use increased with each additional day of medication supplied starting with the third day and risk of use at 1-year doubled with a 5-day prescription. The ***sharpest increases in chronic opioid use*** observed: 1) after the 5th and 31st day on therapy (see figure), 2) if a patient received a second opioid prescription, 3) an initial 10- or 30-day supply, or 4) if the prescription was over a 700 morphine milligram equivalents cumulative dose (e.g. oxycodone 10mg PO q6h PRN pain x 14 days = 840 MME). The highest probability of continued opioid use at 1 and 3 years was observed among patients who started on a long-acting opioid. Of note, in this large population, approximately 70% of patients had an initial duration of opioids of ≤7 days, but 7.3% were initially prescribed opioids for ≥31 days.

The rates are not as high after surgery, but a recent report notes that approximately 5-7% of opioid-naïve patients undergoing major or minor surgery will become persistent opioid users (continued use >3 months from surgery).

In summary, for cancer-free, previously opioid-naïve medical patients receiving a 10-day supply of opioids, approximately 20% are still using opioids after 1 year, and for patients receiving a 30-day supply as their initial opioid prescription, approximately 35% are still using opioids after 1 year. These rates are approximately 5-7% for surgical patients. Thus, care should be taken to a) maximize use of scheduled non-opioid analgesics and b) use opioids for the shortest duration possible.

© 2022 McEvoy MD et al. *JAMA Network Open.*

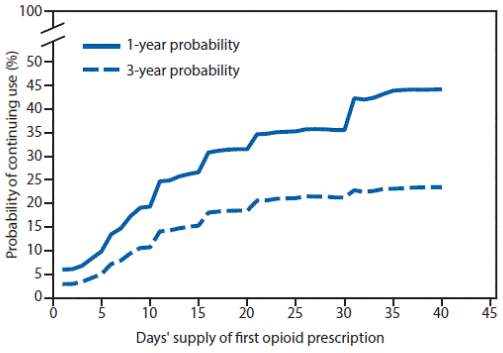

References:
https://www.cdc.gov/mmwr/volumes/66/wr/mm6610a1.htm?s_cid=mm6610a1_e
Brummett CM, et al. JAMA Surg. 2017;[Epub ahead of print]

Question 15: Avoid Long-acting – [ALLEN/RICE]
Question: A 60-year-old man with chronic pancreatic pain who takes immediate release morphine is admitted with complaints of poorly controlled pain. Initiation of controlled release morphine compared to starting gabapentin will increase the likelihood of which of the following adverse events?

Answers:

A. Pulmonary aspiration event
B. Intentional overdose
C. Cardiovascular death
D. Reduced pain control

Key point: Initiation of long acting opioids results in higher mortality from out-of-hospital death, cardiovascular death, unintentional overdose, and other causes of death. There is no evidence that long-acting opioids results in improved pain control, but evidence does exist for use of multimodal analgesics to improve pain control in non-cancer pain.

Rationale: Long-acting opioids increase the risk of unintentional overdose deaths but also may increase mortality from cardiorespiratory and other causes. A recent study was performed in Tennessee (see Reference) to compare all-cause mortality for patients with chronic non-cancer pain and no evidence of palliative or end-of-life care who were prescribed either long-acting opioids or alternative medications for moderate to severe chronic pain. Propensity score-matching was used to compare new episodes of prescriptions for long-acting opioids versus either analgesic anticonvulsants (e.g. gabapentinoids) or low-dose cyclic antidepressants (control medications). Total and cause-specific mortality were determined from death certificates. Of 22,912 new episodes of prescribed therapy for both long-acting opioids and control medications, the HR for total

© 2022 McEvoy MD et al. *JAMA Network Open.*

mortality was 1.64 (95% CI, 1.26-2.12) for those prescribed long-acting opioids. Increased risk was due to out-of-hospital deaths (HR, 1.90; 95% CI, 1.40-2.58). For out-of-hospital deaths other than unintentional overdose, the HR was 1.72 (95% CI, 1.24-2.39). The HR for cardiovascular deaths was 1.65 (95% CI, 1.10-2.46). The HR during the first 30 days of therapy was 4.16 (95% CI, 2.27-7.63).

This recent data, taken in context of all that is known about chronic opioid use, would suggest that maximizing non-opioid analgesics (e.g. acetaminophen, NSAIDs, gabapentinoids, and antidepressants), is preferable to starting long-acting opioids for non-cancer, non-palliative pain management. As this form of pain management can be complex and nuanced, it is appropriate to seek a consult from a pain management expert prior to initiating long-acting opioids. In summary, prescription of long-acting opioids for chronic non-cancer pain compared to anticonvulsants or cyclic antidepressants was associated with a significantly increased risk of all-cause mortality, including deaths from causes other than overdose.

Reference:
Ray WA, Chung CP, Murray KT, Hall K, Stein CM. Prescription of Long-Acting Opioids and Mortality in Patients With Chronic Noncancer Pain. JAMA. 2016;315(22):2415-2419.

© 2022 McEvoy MD et al. *JAMA Network Open.*

Question 16: opioid schedules II v IV
Question: Which of the following statements is correct concerning prescriptions for Schedule I-V medications in routine situations?

Answers:

A. Schedule I narcotic prescriptions must be written/printed to be filled or refilled
B. Schedule II narcotic prescriptions can be written, faxed, or phoned in to be filled or refilled
C. Schedule III narcotic prescriptions can be refilled up 11 times in 12 months
D. Schedule IV narcotic prescriptions can be written, faxed, or phoned in to be filled

Key Point: Prescriptions for **schedules III through V** controlled substances may by **written, oral or transmitted by fax.** Prescriptions for **schedules III and IV** controlled substances may be **refilled up to 5 times in 6 months.** All prescriptions for schedule II-IV medications should include the **dose, frequency, and number of pills to be filled,** as well as the **maximum number of pills to be taken in a 24 hour period**.

Rationale: Drugs and other substances that are considered controlled substances under the Controlled Substances Act (CSA) are divided into five schedules.

Schedule V has low risk of abuse and prescriptions for **schedule V** controlled substances may be **refilled as authorized by the practitioner.**

Alternatively, **Schedule I** narcotics have no currently accepted medical use in the United States, a lack of accepted safety for use under medical supervision, and a high potential for abuse (e.g. heroin, LSD).

Schedule II narcotics have a high potential for abuse which may lead to severe psychological or physical dependence (e.g. hydrocodone, hydromorphone (Dilaudid®), oxycodone (OxyContin®, Percocet®)). **Prescriptions for schedule II controlled substances must be written and be signed by the practitioner. Prescriptions for schedule II controlled substances cannot be refilled. A new prescription must be issued. In emergency situations, a prescription for a schedule II controlled substance may be telephoned to the pharmacy and the prescriber must follow up with a written prescription being sent to the pharmacy within 7 days.**

Schedule III substances have a potential for abuse less than substances in Schedules I or II and abuse may lead to moderate or low physical dependence or high psychological dependence (e.g. products containing not more than 90 milligrams of codeine per dosage unit (Tylenol with Codeine®), and buprenorphine (Suboxone®).

Schedule IV substances have a low potential for abuse relative to substances in Schedule III (e.g. tramadol (Ultram), alprazolam (Xanax®), carisoprodol (Soma®), clonazepam (Klonopin®)). However, the combination of Schedule II and IV medications puts a patient at greater risk of drug-related overdose.

Prescriptions for **schedules III through V** controlled substances may by **written, oral or transmitted by fax.** Prescriptions for **schedules III and IV** controlled substances may be **refilled up to 5 times in 6 months.** All prescriptions for schedule II-IV medications should include the **dose, frequency, and number of pills to be filled,** as well as the **maximum number of pills to be taken in a 24 hour period**.

© 2022 McEvoy MD et al. *JAMA Network Open.*

For treating acute conditions, the fewest number of pills expected to be needed should be prescribed in order to reduce potential for abuse, dependence, or diversion. **One potential aid in such a strategy would be to describe tramadol for short courses of therapy (Schedule IV) rather than oxycodone (Schedule II), as a refill of tramadol could be given without necessitating a return to clinic or hospital (via fax or phone).**

References: **https://www.deadiversion.usdoj.gov/schedules/index.html**

© 2022 McEvoy MD et al. *JAMA Network Open.*

Question 17: Tramadol – benefits [MCEVOY/RICE]

Question: Which of the following is TRUE of tramadol (Ultram) compared to either morphine or oxycodone:

Answers:

A. Increased risk of constipation
B. Increased risk of respiratory depression
C. **Increased risk of serotonin syndrome**
D. Increased risk of dependance

Key Point:
There is a risk of serotonin syndrome with tramadol use for patients who are taking SSRIs, SNRIs, and MAOIs.

Rationale: In the setting of musculoskeletal pain, the atypical, centrally acting analgesic tramadol (Ultram) offers important benefits. Its multi-modal effect results from a dual mode of action, ie, opioid and monoaminergic mechanisms, with efficacy in both nociceptive and neuropathic pain. Moreover, fewer instances of side effects such as constipation, respiratory depression, and sedation occur than with traditional opioids, and tramadol has been prescribed for 30 years for a broad range of indications. Tramadol is now regarded as the first-line analgesic for many musculoskeletal and post-surgical indications.

Additional benefits of using tramadol for acute postoperative or non-surgical pain in addition to scheduled non-narcotic multimodal analgesics is that it is a Schedule IV medication, which allows for refills to be authorized by phone. Thus, a prescription for a very short course of tramadol could be given at discharge (e.g. 50mg q6h PO x 2-3 days, total pills = 12) and then refilled over the phone without the patient having to return for a written prescription, if needed. This would allow for safer narcotic prescribing for the patient and the community.

However, it should be noted that there is a risk of serotonin syndrome with tramadol use for patients who are taking SSRIs, SNRIs, and MAOIs. Even with the benefits listed above, tramadol use should be avoiding in patients taking these medications.

References:

Qaseem A, et al. Noninvasive Treatments for Acute, Subacute, and Chronic Low Back Pain: A Clinical Practice Guideline From the American College of Physicians. Ann Intern Med. 2017 (https://www-ncbi-nlm-nih-gov.proxy.library.vanderbilt.edu/pubmed/28192789)

© 2022 McEvoy MD et al. *JAMA Network Open.*

Derry S, et al. Single fixed-dose oral dexketoprofen plus tramadol for acute postoperative pain in adults. Cochrane Database Syst Rev. 2016:CD012232. (https://www-ncbi-nlm-nih-gov.proxy.library.vanderbilt.edu/pubmed/27654994)

© 2022 McEvoy MD et al. *JAMA Network Open.*

Question 18: Tramadol contraindications [MCEVOY/FLEMING]

Question: Which of the following medications is safe to use in a pain regimen along with tramadol?

Answers:

A. **Pregabalin (Lyrica)**
B. Duloxetine (Cymbalta)
C. Citalopram (Celexa)
D. Rasagiline (Azilect)

Key Point: Tramadol use with SSRIs, SNRIs, and MAOIs should be undertaken with caution, if at all.

Rationale: Since chronic pain syndromes and depression simultaneously occur in many patients, it is likely that tramadol and an SSRI, SNRI, or MAOI could be used together. Concomitant use of these drugs is known to increase the risk for seizures and serotonin syndrome. Tramadol is a mu-opioid receptor agonist as well as an inhibitor of the reuptake of norepinephrine and serotonin in the central nervous system (CNS). SSRIs contribute to this interaction by increasing serotonin levels in the CNS and may also inhibit the metabolism of tramadol via CYP2D6, thereby increasing the concentration of tramadol. The coadministration of tramadol and SSRI antidepressants should be prescribed with caution, if at all. This interaction is also possible with SNRIs and MAOIs.

References:

Beakley BD. Tramadol, Pharmacology, Side Effects, and Serotonin Syndrome: A Review. Pain Physician 2015; 18:395-400 (http://www.painphysicianjournal.com/current/pdf?article=MjM3OA%3D%3D&journal=89)

http://www.ebmconsult.com/articles/tramadol-interaction-ssri-serotonin-syndrome-mechanism

© 2022 McEvoy MD et al. *JAMA Network Open.*

Question 19: OPIOIDS: RISK WITH OTHER SEDATIVES [LONERGAN/FLEMING]

Question: A 32 yo female is being discharged home after a cesarean delivery two days ago. She has taken hydrocodone and alprazolam for over two years for low back pain and severe muscle spasms. Which of the following statements is correct regarding the combination of hydrocodone and alprazolam as you prepare her post-discharge analgesic regimen?

Answers:

A. This combination is an effective treatment for long-term musculoskeletal pain and increasing the hydrocodone dose temporarily for acute postoperative pain does not increase patient risk
B. As this combination has been used for long-term treatment of pain in this patient, there is no benefit of adding non-opioid multimodal analgesics for acute postoperative pain
C. **This combination increases the risk for serious adverse effects such as respiratory depression and death and thus any increase of opioid dosing for acute or chronic pain should be undertaken with caution**
D. As this combination is not an effective treatment for musculoskeletal pain and poses risk to the patient, these should be discontinued and a different analgesic combination should be used.

Key Point: Combinations of opioids and benzodiazepines places patients at greater risk of serious adverse events and death than the use of either alone. In the acute perioperative period, increasing doses of these medications, especially upon discharge, can place the patient at increased risk of adverse events.

Rationale: In 2016, the FDA required changes to drug labeling to require box warnings to health professionals and patients regarding the serious risks associated with combined use of opioids and benzodiazepines. Neither opioids or benzodiazepines have been shown to be an effective treatment for chronic low back pain and their use, especially in combination, is associated with serious risk for respiratory depression and death. Utilizing these medications for physiologic “legitimate” pain does not reduce the risk for physiologic dependence, addiction, or serious adverse events. In fact, combinations of opioids and benzodiazepines places patients at greater risk serious adverse events and death than the use of either alone. Based upon these risks, the most recent guidelines from the American Society of Interventional Pain Physicians recommends to “not combine opioids with sedative hypnotics, benzodiazepines, or barbiturates for chronic, non-cancer pain unless there is a specific medical indication for the combination.”

In the acute perioperative period, increasing doses of these medications, especially upon discharge to a non-monitored setting, can place the patient at increased risk of adverse events. Although continuing this medication combination would not be “absolutely contraindicated,” in this scenario the physician should encourage a multidisciplinary treatment plan to address the

acute postoperative pain in the setting of chronic low back pain. The long-term plan should explore addiction risk factors and transition away from potentially harmful and addictive medications. The short-term plan should take into account the increased risk of adverse events with these combinations and consider: 1) appropriate scheduled use of non-sedating opioid adjuncts, 2) coordinating with the healthcare professional who is providing the chronic narcotics to the patient, 3) patient education concerning the goal of optimal analgesia, which takes into account functional recovery and side effects in addition to pain ratings, and 4) consideration of consultation with a chronic pain medicine expert.

References

Jones J, et al. *Drug and Alcohol Dependence,* 2012;125:8-18
Manchikanti L, et al. *Pain Physician,* 2017;20:S3-S92.
Scott MJ, et al. *Peioperative Med*, 2017

Question 20: OPIOIDS: CHRONIC PAIN/OPIOID USE [LONERGAN/FLEMING]

Question 20: A patient is admitted to your in-patient service. Review of her medical record reveals that she has used extended-release morphine for several years to manage chronic low back pain. Based on this information alone, what is the approximate likelihood that this patient has an opioid addiction?

Answers:
A. <5%
B. 10%
C. 25%
D. 40%

Key Point: Opioid **<u>addiction</u>** is a primary, chronic, and relapsing brain disease characterized by an individual pathologically pursuing reward and/or relief by substance use. The term addiction implies a pattern of maladaptive behavior characterized by ongoing use despite harm or potential for harm. This is present in about 10% of chronic opioid users.

Rationale:
Current research suggests that about 1 in 10 (8-12%) chronic pain patients manifest behavior consistent with opioid addiction. Of chronic pain patients, 21-29% have a recent history of opioid misuse. Medical terminology in the field of addiction medicine is rapidly evolving and sometimes confusing.

Opioid **<u>misuse</u>** implies a use that is contrary to what is recommended, such as taking a medication more often than prescribed.

Opioid **<u>abuse</u>** is the intentional use of an opioid for nonmedical purposes, for the euphoric effects or to “get high.”

© 2022 McEvoy MD et al. *JAMA Network Open.*

Opioid **<u>addiction</u>** is a primary, chronic and relapsing brain disease characterized by an individual pathologically pursuing reward and/or relief by substance use. The term addiction implies a pattern of maladaptive behavior characterized by ongoing use despite harm or potential for harm, and this is present in about 10% of chronic opioid users.

<u>Physical dependence</u> upon an opioid is a physiological consequence of chronic use and is not necessarily indicative of behavioral patterns of misuse, abuse, or addiction. The DSM-5 utilizes terminology focused upon "<u>substance use disorders</u>", rather than addiction, and divides these into categories based upon severity: mild, moderate, and severe.

References

Vowles et al. Pain, 2015;156:4
Webster L, et al. Avoiding Opioid Abuse While Managing Pain; 2007.

****

© 2022 McEvoy MD et al. *JAMA Network Open.*

Section 2 Questions: 21-40
[Second exposure to topics]

© 2022 McEvoy MD et al. *JAMA Network Open.*

SECOND STEMS

Question 21:
Question: A 28-year-old opioid-naïve male presents to the emergency department (ED) with a tooth abscess. Evaluation and management of his condition by a physician who frequently prescribes opioids increases the likelihood of which of the following:

Answers:

A. Repeat visit to the ED within 14 days
B. Subsequent use of opioids for more than 180 days after initial ED visit
C. Cardiopulmonary arrest
D. Intentional overdose

Key Point:
In opioid-naïve patients, treatment by a clinician who prescribes opioids more frequently than their peers is **strongly correlated with subsequent long-term opioid use in patients**. Additionally, risk of long-term opioid use increases rapidly even with commonly prescribed doses and durations of therapy.

Rationale:
Increasing overuse of opioids in the United States may be driven in part by prescribing habits of healthcare professionals.

In a recent study of almost 400,000 patients (Barnett ML *et al*, NEJM 2017), ED physicians were categorized as being **high-intensity** or **low-intensity opioid prescribers** according to relative quartiles of prescribing rates within their hospital. L**ong-term opioid use** was defined **≥180 days** of opioids supplied in the 12 months after the index ED visit, ***excluding prescriptions within 30 days after the index visit***. Rates of long-term opioid use were compared in patients treated by high or low-intensity prescribers. Overall, patient characteristics and diagnoses treated were similar across all prescribers. There was **>3-fold increase in opioid prescription rates** by the high-intensity prescribers **as compared to** low-intensity (24.1% vs. 7.3% of ED visits, see figure below). Patients treated by high-intensity opioid prescribers had a **30% increased risk of long-term opioid use**.

A similar study (Deyo RA *et al*, JGIM 2017) evaluating **500,000 opioid-naïve patients** demonstrated that both the number of prescriptions in the **1st month of opioid consumption** and **total morphine milligram equivalents (MMEs) prescribed** were highly correlated with **risk of long-term use**, defined as **≥6 opioid prescriptions** in the subsequent 12 months. After excluding patients with cancer pain and non-cancer chronic pain conditions, compared to the group that only filled 1 prescription in the first month, **those who filled ≥2 were 4 to 10 times as likely to become long-term users.** Additionally, those who were

© 2022 McEvoy MD et al. *JAMA Network Open.*

dispensed **>120 morphine milligram equivalents total** (MME; e.g. 120 MME = oxycodone 5mg PO q6h PRN x 4 days) were **2-16 times** as likely to be **long-term opioid users**, with increasing MME dispensed associated with increased risk.

If a prescription is given for opioids for acute non-cancer pain, **the shortest duration and the lowest number of MMEs (by total dose and pill count) possible should be given**. Per latest CDC guidelines, 3 days of opioid therapy is often sufficient and >7 days is rarely needed for acute pain.

© 2022 McEvoy MD et al. *JAMA Network Open.*

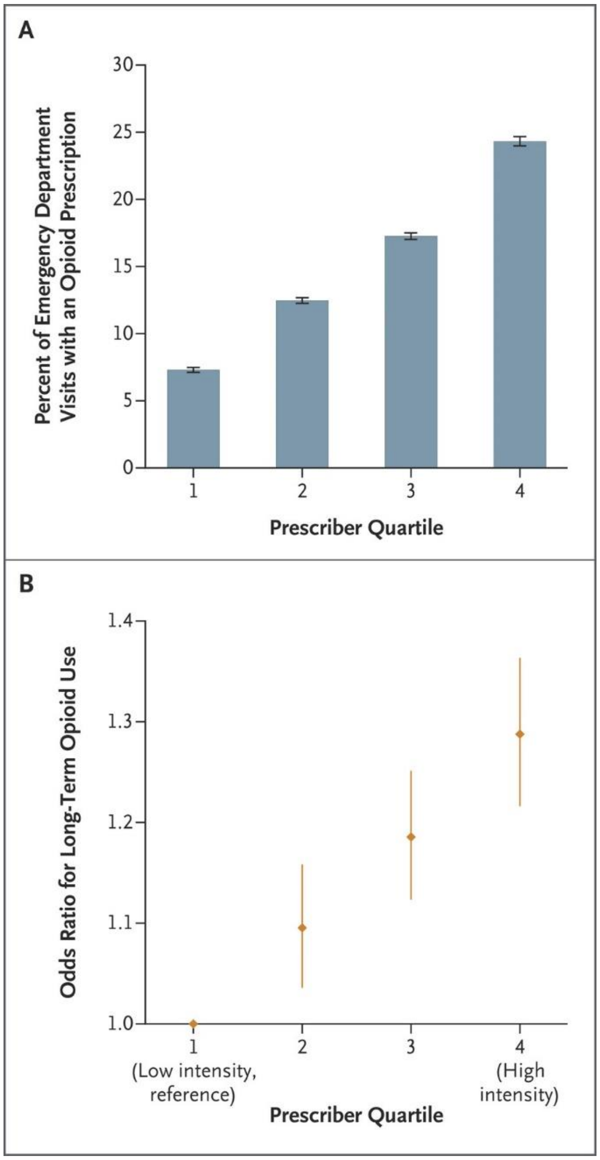

References:

Barnett ML, Olenski AR, Jena AB. Opioid-Prescribing Patterns of Emergency Physicians and Risk of Long-Term Use. *NEJM* 2017;376:663-673.

Deyo RA, Hallvik SE, Hildebran C, Marino M, Dexter E, Irvine JM, O'Kane N, Van Otterloo J, Wright DA, Leichtling G, Millet LM. Association Between Initial Opioid Prescribing Patterns and Subsequent Long-Term Use Among Opioid-Naïve Patients: A Statewide Retrospective Cohort Study. J Gen Intern Med. 2017;32:21-27.

© 2022 McEvoy MD et al. *JAMA Network Open.*

Dowell D, Haegerich TM, Chou R. CDC guideline for prescribing opioids for chronic pain – United States, 2016. JAMA. 2016;315:1624–45.

© 2022 McEvoy MD et al. *JAMA Network Open.*

Question 22:
Question: A 62 year-old F undergoes an emergency exploratory laparotomy for a small bowel obstruction. She has been taking 120mg of sustained release morphine twice a day for 6 months to treat pain from pancreatic cancer. She is currently receiving IV hydromorphone in the PACU with a pain score goal of <4/10. Which of the following statements regarding opioid tolerance in this scenario is correct?

Answers:

A. Constipation is unlikely to occur.
B. Nausea is unlikely to occur.
C. Tolerance to the analgesic effects of the hydromorphone is unlikely to occur.
D. Severe respiratory depression may occur while still experiencing inadequate pain control.

Key Point:
There is differential tolerance to opioid effects, meaning the tolerance to some effects is faster and to a greater degree than other effects. **Tolerance to analgesia occurs the fastest**, **tolerance to respiratory depression effects of opioids is slower and limited**, and there is almost no tolerance to the constipation. Thus, attempting to achieve the same level of analgesia (e.g. <3/10 pain score) in an opioid-naïve patient and chronic opioid user puts the chronic opioid user at higher risk of respiratory depression.

Rationale:
Common side effects from opioids include nausea, vomiting, ileus, tolerance to analgesia, and respiratory depression. Life-threatening **respiratory depression** from opioid use is likely under-reported. The **risk of respiratory depression increases** for patients that have **OSA, morbid obesity, certain neuromuscular disorders, ASA IV status, and the extremes of age**. The risk also increases when a basal rate is added to a PCA. **Of note, patients on long-term opioids often develop tolerance to the analgesic effects of opioids and several of the opioid-induced side-effects. However, tolerance to the respiratory depressant effects is more limited than the tolerance that develops to analgesic effects of opioids (see Figure).** That is, chronic opioid users can develop tolerance to the analgesic effects and not the respiratory depression, thus making it possible to have more risk of severe respiratory depression than opioid naïve patients when achieving equivalent levels of analgesia.

© 2022 McEvoy MD et al. *JAMA Network Open.*

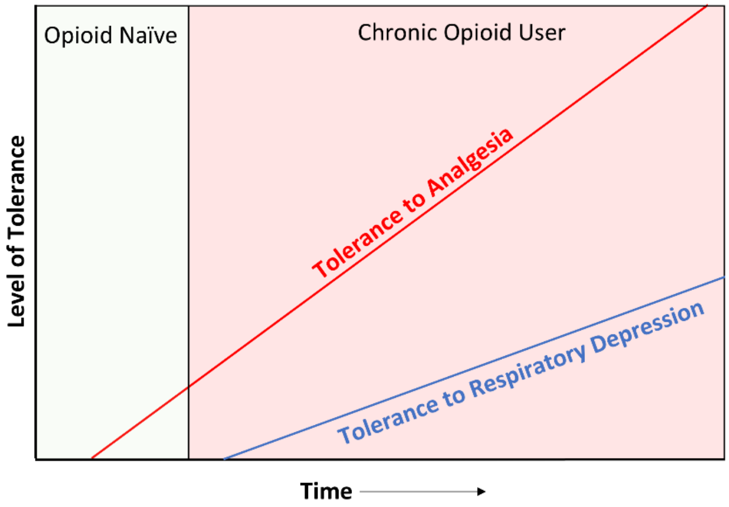

Of additional concern, **constipation** is the most common side effect of opioid administration, affecting **40-95% of patients** treated with opioids and occurring in some even after one dose. **Postoperative ileus** is associated with as little as 2 mg of hydromorphone per day and accounts for prolonged length of stay in many patients. Less common opioid-related side effects include opioid-induced hyperalgesia, sleep disturbance, hallucinations, and immunologic/hormonal dysfunction.

References:

Hayhurst CJ, Durieux ME. Differential Opioid Tolerance and Opioid-induced Hyperalgesia: A Clinical Reality. *Anesthesiology,* 2016, Vol.124, 483-488.
Benyamin R, Trescot AM, Datta S, et al. Opioid complications and side effects. *Pain Physician*, 2008 Mar;11(2 Suppl):S105-20.
Dahan A, Aarts L, Smith TW. Incidence, Reversal, and Prevention of Opioid-induced Respiratory Depression. *Anesthesiology*, 2010 Jan;112(1):226-38.

© 2022 McEvoy MD et al. *JAMA Network Open.*

Question 23:
Question: Which of the following is the most appropriate strategy for managing acute pain in the hospitalized medical or surgical patient?

Answers:

A. Simplifying the pain management regimen by using the fewest medications possible
B. Adding non-opioid medications if pain is not controlled with opioids
C. Reducing medication side effects while targeting the fastest functional recovery
D. Employing a treatment regimen that results in the fewest calls to the supervising clinician

Key Point:
In attempting to achieve optimal analgesia for patients not on chronic opioids at home, the guiding principles include: **1) Opioids should be "added last, prescribed only as needed (PRN), on the shortest period of time possible, and taken off of the analgesia regimen first; 2) Non-opioid analgesic medications should be "added first, ordered as scheduled medications, on longer than any other analgesic, and thus taken off of the regimen last."** We call this the “Function First, Opioids Last” Program.

Rationale:
The goal of any pain management regimen should be to deliver ‘optimal analgesia,’ which has ben recently defined "as a technique that **optimizes patient comfort and facilitates the fastest functional recovery with the fewest medication side effects**" (see Figure.) This can be for the **inpatient or outpatient setting and for the surgical or medical** patient. Of note, achieving the goal of 'optimal analgesia' **may not correspond with the lowest pain score/rating possible**. Overall, the combination of analgesic techniques employed is not important as long as it is effective in delivering this goal of ‘optimal analgesia.’ However, **guiding principles** include the following: **1) Opioids should be "added last, prescribed only as needed, on the shortest period of time possible, and taken off of the regimen first; 2) Non-opioids should be "added first, given as scheduled medications, on longer than any other analgesic, and thus taken off of the regimen last."** This includes prescriptions for the inpatient and post-discharge/outpatient settings. Of particular importance, it should be remembered that scheduled NSAIDs + acetaminophen typically result in 30-40% reduction in opioid requirements for acute pain; this goes up even further if gabapentinoids are used.

References:

McEvoy MD, et al. *Peioperative Med*, 2017
Scott MJ, et al. *Peioperative Med*, 2017

© 2022 McEvoy MD et al. *JAMA Network Open.*

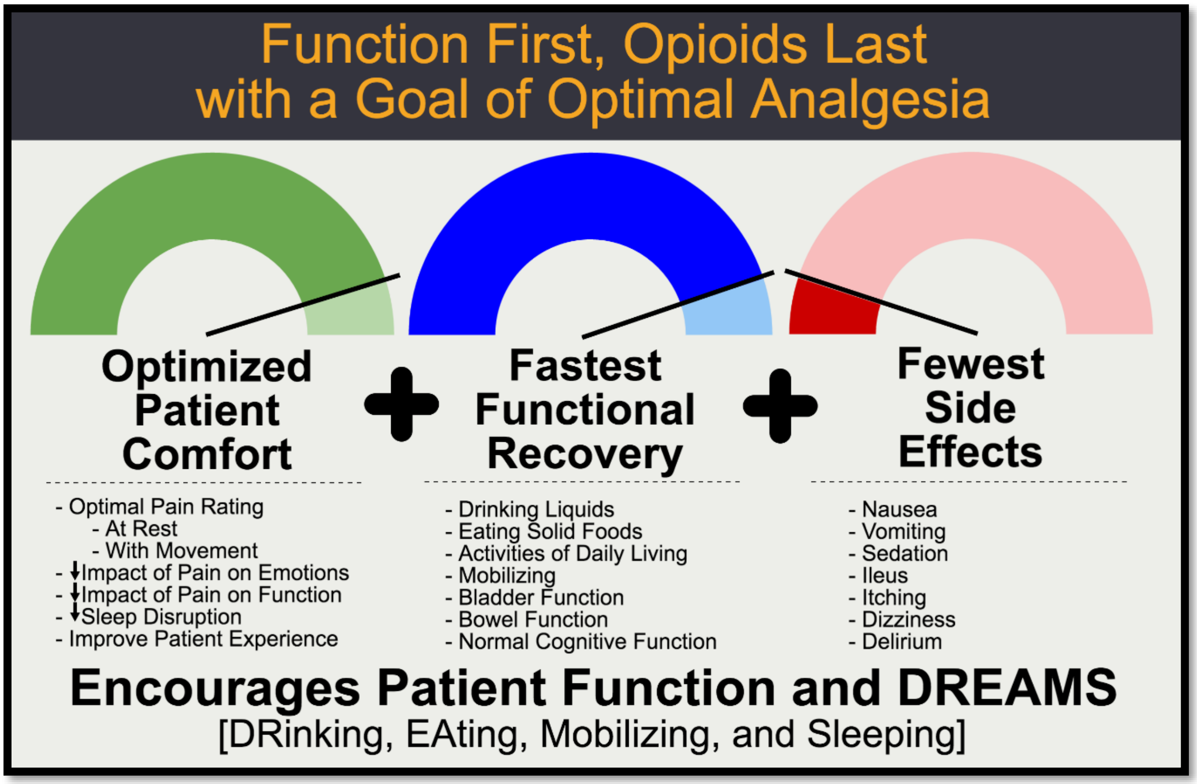

© 2022 McEvoy MD et al. *JAMA Network Open.*

Question 24: Dose-related Risk of Death

Question: A 60 yo male is discharged home after a femur fracture repair with a prescription for "oxycodone 20mg PO q4hrs PRN pain." Compared to a person taking <20 morphine milligram equivalents (MME)/day, his relative increased risk of overdose is approximately?

Anwers:

A. None, since it treating acute pain
B. Approximately 5 times greater risk
C. Approximately 10 times greater risk
D. Approximately 15 times greater risk

Key Point: Oxycodone is 1.5 times as potent as morphine. If the patient uses oxycodone as prescribed then he will be taking 120mg oxycodone per day, or 180 milligram morphine equivalents (MME). Opioid doses >100 MME/day are associated with 2 to 8 times the risk for overdose compared to people taking <20 MME/day.

Rationale:

To assess patient risk for prescribing opioids, clinicians should convert to milligram morphine equivalents (MME)/day. Oxycodone is 1.5 times as potent as morphine. If the patient uses oxycodone as prescribed then he will be taking 120mg oxycodone per day, or 180 milligram morphine equivalents (MME).

One good-quality population-based, nested case-control study also found a dose-dependent association with risk for overdose death. Relative to 1–19 MME/day, the adjusted odds ratio (OR) was 1.32 for 20–49 MME/day, 1.92 for 50–99 MME/day, **2.04 for 100–199 MME/day**, and 2.88 for ≥200 MME/day.

One large fair-quality retrospective cohort study found that recent opioid use was associated with increased risk for any overdose events and serious overdose events versus nonuse. It also found higher doses associated with increased risk. Relative to 1–19 MME/day, the adjusted hazard ratio (HR) for any overdose event (consisting of mostly nonfatal overdose) was 1.44 for 20 to 49 MME/day, 3.73 for 50–99 MME/day, and **8.87 for ≥100 MME/day**. A similar pattern was observed for serious overdose.

Based on available literature, opioid doses >100 MME/day are associated with approximately 5 times the risk for overdose compared to people taking <20 MME/day. Of note, <20 MME/day is equal to <3 oxycodone 5mg tablets per day.

References:

Dunn KM, et al. Ann Intern Med 2010;152:85–92. PubMed
Gomes T, et al. Arch Intern Med 2011;171:686–91. PubMed
https://www.cdc.gov/drugoverdose/pdf/calculating_total_daily_dose-a.pdf

© 2022 McEvoy MD et al. *JAMA Network Open.*

Question 25– Opioids – CSMD/prescribing laws [Edwards/Ehrenfeld]
Question: A 43-year old female undergoes a right radial fracture repair with open reduction internal fixation. The patient does not take opioid medications at home. After her procedure, she is prescribed hydrocodone for pain management. Choose the correct statement regarding the Tennessee Laws on opioid prescribing:

Answers:

A. **A prescriber must check the CSMD for any new opioid or benzodiazepine prescription provided for longer than 7 days.**
B. A prescriber does not need to check the CSMD before prescribing opioids at discharge because this patient was already on opioids prior to being admitted to the hospital.
C. A prescriber must check the Tennessee Controlled Substance Database (CSMD) before prescribing opioid to any patient at hospital discharge.
D. A prescriber does not need to check the CSMD prior to giving this patient a discharge benzodiazepine prescription.

Key Point:
In 2016, the Tennessee House Bill 2571 was passed into law requiring all prescribers (or their designated healthcare practitioner extenders) to check the CSMD prior to prescribing or dispensing controlled substances at the beginning of a new episode of treatment, except for a non-refillable prescription as part of a treatment for a surgical procedure that occurred in a licensed healthcare facility and **is limited to a 7-day supply.**

Rationale:
In 2016 Tennessee House Bill 2571 was passed into law requiring all prescribers (or their designated healthcare practitioner's extenders) to check the CSMD prior to prescribing or dispensing controlled substances at the beginning of a new episode of treatment. A new episode of treatment means a prescription that has not been prescribed or dispensed by that prescriber or dispensing practice within the previous twelve months. Controlled substances include all opioids and benzodiazepines. Prescribers are not required to check the CSMD if one or more of the following are met:

A. The controlled substance is prescribed or dispensed for a patient who is currently receiving hospice care;
B. The controlled substance is prescribed or dispensed to a patient as a non-refillable prescription as part of a treatment for a surgical procedure that occurred in a licensed healthcare facility and **is limited to a 7-day supply**; or
C. The controlled substance is prescribed for administration directly to a patient during the course of inpatient or residential treatment in a hospital or nursing home.

http://www.capitol.tn.gov/Bills/109/Bill/HB2571.pdf

© 2022 McEvoy MD et al. *JAMA Network Open.*

Question 26: Use of Multimodal First [EDWARDS/Ehrenfeld]
Question: A 34-year-old female presents with severe right-sided lower quadrant abdominal pain consistent with uterine fibroid pain diagnosed by pelvic exam and ultrasound. The patient's pain remains severe despite mifepristone and with oxycodone 10 mg PO q4 hrs PRN for pain. Labs reveal mild anemia no abnormalities on CBC or BMP. What is the most appropriate next step in pain management for this patient?

Answers:

A. Initiation of extended-release oxycodone
B. **Addition of scheduled acetaminophen and gabapentin**
C. Initiation of hydromorphone IV PRN
D. Increased frequency of oral oxycodone dosing

Key point: Patients with poorly controlled pain should have multimodal non-narcotic medications initiated BEFORE escalation of opioid therapy.

Rationale:
Ordering oxycodone 10 mg q4 hrs PRN can result in administration of up to 60 mg PO oxycodone in a 24 hour period, roughly equivalent to 90 mg morphine milligram equivalent (MME) per day. Risk of overdose death increases above 20 MME and is almost 2-8 times higher at >100 MME/day. Of note, elderly patients require lower doses of opioids and are at higher risk for respiratory depression than younger patients.

Alternatively, use of scheduled non-opioid multimodal analgesics such as acetaminophen, NSAIDs, and gabapentin have been shown to decrease opioid requirements for patients with acute pain by 30-50%. Their initiation may allow better pain control and decreased opioid requirements. This approach is preferred over increasing short acting opioids or adding IV opioids to the regimen.

In the setting of acute pain, IV opioids should be reserved for scenarios in which the patient is unable to tolerate oral intake, requires strict NPO status, or for rescue analgesia if patient has severe pain. Long-acting opioids should be avoided and only reserved for treating chronic pain, if at all.

In summary, when treating medical or surgical patients with acute pain the **guiding principles** include: **1) Opioids should be "added last, prescribed only as needed, should be on the shortest period of time possible, and taken off of the regimen first; 2) Non-opioids should be "added first, given as scheduled medications, on longer than any other analgesic, and thus taken off of the regimen last."** This includes prescriptions for the inpatient and post-discharge/outpatient settings.

References:

© 2022 McEvoy MD et al. *JAMA Network Open.*

Macintyre PE, Jarvis DA. PAIN. 1996;64(2):357-364.
Cepeda M. Clinical Pharmacology & Therapeutics. 2003;74(2):102-112.
https://www.tn.gov/assets/entities/health/attachments/ChronicPainGuidelines.pdf
Kong VKF, Irwin MG. Br J Anaesth. 2007;99(6):775-786

© 2022 McEvoy MD et al. *JAMA Network Open.*

Question 27: Non-opioid analgesics [McEvoy/Ehrenfeld]
Question: Using appropriate doses of a combination of SCHEDULED acetaminophen and ibuprofen for acute pain results in approximately what degree of opioid reduction?

Answers:

A. 10%
B. 25%
C. 40%
D. 70%

Key Point:
Use of a combination of scheduled non-opioid multimodal analgesics such as acetaminophen and NSAIDs have been shown to decrease opioid requirements for patients with acute pain by 30-50%.

Rationale:
Use of a combination of scheduled non-opioid multimodal analgesics such as acetaminophen, NSAIDs, and gabapentin have been shown to decrease opioid requirements for patients with acute pain by 30-50%. Their initiation may allow overall better pain control than with opioids alone. As with any medication, appropriate dosage should be administered, which is a maximum dose of 800mg 10mg/kg q6h for ibuprofen and 1000mg or 15 mg/kg q6h for acetaminophen, assuming no dosage adjustments are needed for liver or kidney disease. For gabapentin, starting at 300 mg TID is reasonable for patients <70 yo, and 100 mg TID for those >70 yo. However, as gabapentin clearly has a dose-response effect, rapid escalation in the inpatient setting to the highest does without side effects (e.g. sedation, dizziness) is appropriate. Alpha-2 agonists have also shown benefit, but caution should be taken with patients at risk of hypotension and/or bradycardia.

In summary, ibuprofen (or other NSAID) and acetaminophen should be scheduled at the maximum appropriate dose for patient weight, whereas gabapentin can be started at a reasonable dose and quickly titrated up to the optimal dose, which is the highest dose without side effects. Additionally, when treating medical or surgical patients with acute or chronic pain, the **guiding principles** for use of multimodal medications include that they **should be a) added first, b) given as scheduled medications, c) on longer than any other analgesic, and d) taken off of the pain regimen last.**

These principles include orders/prescriptions for the inpatient and post-discharge/outpatient settings.

References:

Sutton CD, et al. Anesthesiol Clin. 2017;35:107-124 (https://www-ncbi-nlm-nih-gov.proxy.library.vanderbilt.edu/pubmed/28131114)

© 2022 McEvoy MD et al. *JAMA Network Open.*

Li JMW. Med Clin North Am. 2008;92:371-385 (https://www-ncbi-nlm-nih-gov.proxy.library.vanderbilt.edu/ pubmed/18298984)

Scott MJ, et al. *Peioperative Med*, 2017;6:7

© 2022 McEvoy MD et al. *JAMA Network Open.*

Question 28: NSAIDS and safe dose range [ROBERTSON/MCEVOY]
Question: A 71 yo M with history of hypertension is POD1 after colectomy. Labs and hydration status are normal. Which of the following is the recommended maximum daily dose for this patient?

Answers:

A. 120 mg
B. 60 mg
C. 30 mg
D. ketorolac should be avoided in this patient

Key point: Dose reductions for ketorolac should occur with patients >65y, weight <50kg, and renal impairment (CrCl<30).

Rationale:
Ketorolac is an NSAID with high COX-1 selectivity that is indicated to treat moderate to severe pain, and is widely used after surgery. It is available in IV, IM, and PO forms. Prior studies have found 30mg of IM ketorolac to have similar analgesic efficacy as 12mg of IV Morphine.[1] Its use in multimodal analgesia regimens provides significant opioid reduction and reduced side effects.[2]

Due to its nature as an NSAID however, there are several side effects and contraindications to use, especially in the post-surgical population. It is only approved for 5 days of total use, due to increased risk of GI bleeding. Dose reduction should be considered when prescribing to those over 65 (see table) and the oral form is not approved for children. Ketorolac is contraindicated in renal failure or risk of renal failure, in patients with active peptic ulcer disease or recent GI bleed, suspected or confirmed cerebrovascular bleeding or concurrently with other NSAIDs. There is controversy surrounding whether ketorolac inhibits bone healing after orthopedic procedures. Additionally, ketorolac is contraindicated after CABG due to a black box warning surrounding all NSAIDs. However, some reports suggest safety, and even better outcomes, with its use.

Table 2. Adult Dosing and Administration of Parenteral NSAIDs Used for Pain Control

Generic	Formulation	Dosing	Max Daily Dose
Ketorolac tromethamine	IM[a]	*Single dose:* Age <65 y: one 60 mg dose; age ≥65 y, renally impaired and/or BW <50 kg: one 30 mg dose *Multiple dose:* Age <65 y: 30 mg every 6 h	If receiving multiple-dose treatment and age <65 y: 120 mg If receiving multiple-dose treatment and age ≥65 y: 60 mg
Ketorolac tromethamine	IV[b]	*Single dose:* Age <65 y: one 30 mg dose; age ≥65 y, renally impaired and/or BW <50 kg: one 15 mg dose *Multiple dose:* Age <65 y: 15 mg every 6 h	If receiving multiple-dose treatment and age <65 y: 120 mg If receiving multiple-dose treatment and age ≥65 y: 60 mg
Ibuprofen (IVIb)	IV[c]	400-800 mg every 6 h as needed	3,200 mg

BW: body weight; max: maximum. [a] Inject slowly and deeply in the muscle. [b] Bolus push over at least 15 seconds. [c] Infuse over at least 30 minutes. Source: References 8, 9.

In many patients, ketorolac is a very effective non-opioid analgesic for use in medical or surgical patients with moderate to severe pain. Practical guidelines are shown in the Table. The maximum recommended daily dose for the patient presented in this question is 60mg per day IV.

References:

© 2022 McEvoy MD et al. *JAMA Network Open.*

1 Yee JP, Koshiver JE, Allbon C, Brown CR. Comparison of intramuscular ketorolac tromethamine and morphine sulfate for analgesia of pain after major surgery. *Pharmacotherapy* 1986; **6**: 253-61
2 Cepeda MS, Carr DB, Miranda N, Diaz A, Silva C, Morales O. Comparison of morphine, ketorolac, and their combination for postoperative pain: results from a large, randomized, double-blind trial. *Anesthesiology* 2005; **103**: 1225-32

Question 29: Scheduled multimodals [McEvoy/Ehrenfeld]
Question: An opioid-naïve 24 yo F with history of cesarean delivery (CD) undergoes repeat CD. Which of the following represents the best initial plan for postoperative analgesia?

Answers:

A. scheduled acetaminophen, ibuprofen, and gabapentin with oral opioids PRN
B. scheduled acetaminophen and ibuprofen with oral opioids PRN
C. oral opioids, acetaminophen, and ibuprofen PRN
D. intravenous patient-controlled analgesia (PCA) for 24 hours

Key Point: Multimodal non-opioid analgesics, particularly acetaminophen and ibuprofen, have been shown to reduce opioid consumption by 30-50% when used in a scheduled regimen. These medications should be scheduled prior to initiating any opioid therapy and opioids should initially be ordered PRN. Recommendations are to add gabapentin if initial first-line scheduled use of multimodal analgesics is not sufficient.

Table 2
Suggested analgesic protocol for post–cesarean delivery in-hospital pain management

Setting	Drug	Dose and Route	Prescribing Information
Standard care[a] (prescribed at time of surgery)	Neuraxial morphine	Preferred: intrathecal morphine 100–150 μg or epidural morphine 2–3 mg after delivery	With intrathecal hyperbaric bupivacaine 12 mg and fentanyl 15 μg<br>With epidural 2% lidocaine 15–25 mL (±bicarbonate and epinephrine) ± fentanyl 50–100 μg
	NSAIDs	Ibuprofen 600 mg PO (or ketorolac 15 mg IV if NPO)	Every 6 h (scheduled) for 48–72 h after cesarean delivery
	Acetaminophen	Acetaminophen 650 mg PO (or IV if NPO)	Every 6 h (scheduled) for 48–72 h after cesarean delivery
	Oral opioids	Oxycodone 5–10 mg PO	As needed for breakthrough pain: VNPS ≤4/10: 5 mg VNPS >4/10: 10 mg
Ongoing or severe postoperative pain[b]	IV opioids	IV morphine, fentanyl, or hydromorphone	Intermittent IV boluses or IV patient-controlled analgesia
	Regional anesthesia	Bilateral TAP block	0.25% ropivacaine 20–25 mL per side
	Oral adjuvants	Gabapentin	600 mg PO rescue dose (300 mg PO every 8 h for ongoing severe pain)
		Dexamethasone	4–8 mg PO

Rationale:
Use of scheduled non-opioid multimodal analgesics such as acetaminophen, NSAIDs (e.g. ibuprofen or ketorolac), and gabapentin have been shown to decrease opioid requirements and improve patient satisfaction when used at appropriate doses. As with any medication, appropriate dosage should be administered. For most women, this will be ibuprofen 600-800mg PO q6h (ketorolac 15-30 mg IV q6h) and acetaminophen 1000 mg PO q6h. For gabapentin, in the peripartum period it is reasonable to start with an initial dose of 600 mg PO and then order 300 mg TID. However, as gabapentin clearly has a dose-response effect, rapid escalation in the inpatient setting to the highest does without side effects (e.g. sedation, dizziness) is appropriate.

In summary, ibuprofen (or other NSAID) and acetaminophen should be scheduled at the maximum appropriate dose for patient weight, whereas gabapentin can be started at a reasonable dose and quickly titrated up to the optimal dose if the initial multimodal agents are not sufficient (see Table).

When treating medical or surgical patients with acute or chronic pain, the **guiding principles** for use of multimodal medications include that they **should be a) added first, b) given as scheduled medications, c) on longer than any**

© 2022 McEvoy MD et al. *JAMA Network Open.*

other analgesic, and d) taken off of the pain regimen last. These principles include orders/prescriptions for the inpatient and post-discharge/outpatient settings.

References:

Sutton CD, et al. Anesthesiol Clin. 2017;35:107-124 (https://www-ncbi-nlm-nih-gov.proxy.library.vanderbilt.edu/pubmed/28131114)

© 2022 McEvoy MD et al. *JAMA Network Open.*

Question 30: APAP and safe dose question:
Question 30:
Acetaminophen has been shown to be effective at treating mild to moderate post-surgical pain. Which of the following would be an optimal dosing strategy for most effective pain control that does not exceed the recommended daily limit.

Answers:

1. 650mg every 6 hours
2. **1000mg every 6 hours**
3. 325mg every 4 hours
4. 1000 mg every 8 hours

Key Point: In a prospective, randomized, double-blind, placebo-controlled study of adults with post-surgical pain, 1000mg of acetaminophen was superior to 650mg dose for pain control.

Rationale:
Acetaminophen is one of the most widely used drugs in the world. Overdose can lead to serious liver injury and death. While exceeding the recommended limit of 4g per 24 hours of acetaminophen appears to be relatively rare in the outpatient setting (3% of usage days), it is more likely to occur when patients combine prescribed medications containing acetaminophen and OTC medications with acetaminophen. This could also occur with combinations containing NSAIDs, such as percodan (oxycodone plus aspirin), that are taken with other NSAIDs. Accordingly, in order to avoid potential overdose, patients should be clearly educated about the risks of combining over the counter pain medications with medications that contain combined formulations, such as Percocet (oxycodone and acetaminophen).

Of note, **in a prospective, randomized, double-blind, placebo-controlled study of adults with post-surgical pain, 1000mg of acetaminophen was superior to 650mg dose for pain control**.[2] The recommended dosing schedule for adults and children over the age of 12 is 650mg every 4 to 6 hours or 1000mg every 6 hours, not to exceed 4 grams in a 24-hour period. **Thus, in order to maximize the effects of acetaminophen, prescribing the patient a scheduled regimen of acetaminophen along with an opioid PRN for rescue analgesia may be safer and of greater benefit than using a combination medication such as Percocet.** Such an approach would follow the pain management principles outlined in the Function First, Opioids Last program: **1) Opioids should be added last, prescribed only as needed, on for the shortest period of time possible, and removed from the regimen first; 2) Non-opioids should be added first, given as scheduled medications, on for a longer timeframe than any other analgesic, and thus removed from the regimen last.**

References:

© 2022 McEvoy MD et al. *JAMA Network Open.*

1 Shiffman S, Rohay JM, Battista D, et al. Patterns of acetaminophen medication use associated with exceeding the recommended maximum daily dose. *Pharmacoepidemiol Drug Saf* 2015; **24**: 915-21
2 Qi DS, May LG, Zimmerman B, et al. A randomized, double-blind, placebo-controlled study of acetaminophen 1000 mg versus acetaminophen 650 mg for the treatment of postsurgical dental pain. *Clin Ther* 2012; **34**: 2247-58 e3

© 2022 McEvoy MD et al. *JAMA Network Open.*

Question 31: **Gabapentinoids Dosing**
A 35 y/o G1P1 female with h/o migraines, IBS and chronic pelvic pain is POD 7 s/p diagnostic laparoscopy. She reports uncontrolled pelvic pain that is the same as her preoperative pain level despite taking Ibuprofen 800mg q6h and Percocet 5mg q4h. You decide to prescribe pregabalin for neuropathic pain. What are the dosing considerations for pregabalin for this patient?

A. An appropriate starting dose for pregabalin is 75mg PO BID
B. Pregabalin should not be used for patients with any renal insufficiency, even with dosing adjustments.
C. Pregabalin has a lower bioavailability after oral administration than gabapentin.
D. The dose of pregabalin should only be increased once per month due to safety concerns.

Key Point: Based on prior research, a safe and effective starting dose in most patients is gabapentin 300mg PO TID or pregabalin 75mg PO BID. Doses can be uptitrated daily in the inpatient setting if monitoring for side effects (e.g. sedation/dizziness) and weekly in the outpatient setting.

Rationale: Gabapentin and pregabalin function as calcium channel antagonists, blocking the release of neurotransmitters. These medications are indicated in the treatment of neuropathic pain, including diabetic peripheral neuropathy, postherpetic neuralgia, central neuropathic pain and fibromyalgia. They have also been shown to be beneficial analgesic adjuncts in a number of postoperative settings. Despite the same mechanism of action, gabapentin and pregabalin have differing pharmacokinetic profiles, which alter effective dosing regimens. Following oral administration, gabapentin has zero-order absorption and its bioavailability varies from approximately 60% at a total daily dose of 900mg to only 33% at doses up to 3600mg per day. Pregabalin follows a linear absorption pattern with a bioavailability of over 90% at all doses. Both gabapentin and pregabalin have a half-life of 6 hours and **primarily undergo renal excretion**. This indicates that increasing doses of pregabalin will likely produce more predictable changes in pain improvement.

Based on prior research, a safe and effective starting dose in most patients is gabapentin 300mg PO TID. Doses can be uptitrated daily in the inpatient setting if monitoring for side effects (e.g. sedation/dizziness) and weekly in the outpatient setting. Serpell conducted a gabapentin dosing study with an initial total daily dose of 300mg, divided into three doses, and titrated up to a **total daily dose** of 900mg per day over the course of 3 days. There were minimal side effects for most patients at this dosing level. For patients who reported an improvement in pain at 900mg per day, further titration was increased over the course of 5 weeks to 2400mg per day. Patients receiving gabapentin showed a statistically significant improvement in pain scores compared to placebo with only mild to moderate side effects reported. The maximum recommended dosing for treatment of pain with gabapentin is currently 3600mg per day. Additionally, *Freynhagen et al.* investigated pregabalin dosing

© 2022 McEvoy MD et al. *JAMA Network Open.*

and determined that doses ranging from **150-600mg per day provided statistically significant improvement in pain scores**. Patients were started at 150mg per day divided into two doses and titrated weekly based on response and tolerability. The study showed a low dropout rate due to side effects. The current maximum recommended dose of pregabalin is 600mg per day, but some studies have reported higher doses. **Due to primary renal excretion with both gabapentin and pregabalin, dosing should be reduced based on creatinine clearance with additional doses given after hemodialysis**. When ordering as an inpatient at VUMC, q8h is preferred (i.e. 0600, 1400, 2200) due to uneven dosing intervals with TID (i.e. 1000, 1400, 2200).

References:

1. Serpell MG. *Pain* 2002; 99(3): 557-566.
2. Freynhagen R, et al. *Pain* 2005; 115(3): 254-63.
3. Moore RA, et al. *Cochrane Database Syst Rev* 2014; 27(4).
4. Moore RA, et al. *Cochrane Database Syst Rev* 2009; 8(3).

© 2022 McEvoy MD et al. *JAMA Network Open.*

Question 32: Gabapentinoids Side Effects
Question: Gabapentin therapy is initiated in a patient for treatment of radicular back pain. Which of the following side effects is the patient most likely to experience?

Answers:

- **A.** Rash
- **B. Somnolence**
- C. Nausea
- D. Confusion

Key Point: Systematic reviews of the literature concerning gabapentinoid use in medical and surgical patients support substantial analgesic benefit (30-50% reduction in pain scores) with relatively common minor side effects (e.g. somnolence), but no serious adverse events (e.g. respiratory depression) when compared to placebo.

Rationale: A Cochrane review of pregabalin demonstrated benefit at doses ranging from **300-600mg per day** for diabetic peripheral neuropathy (DPN), postherpetic neuralgia (PHN), central neuropathic pain (CNP) and fibromyalgia. Lower dosing regimens were needed for PHN and DPN compared to fibromyalgia in order to reach a **30-50% reduction in pain scores**. At a dose of 600mg/day, dizziness was the most common side effect, occurring in 27-46% of patients, with rates of somnolence ranging from 15-25%. Discontinuation due to intolerable side-effects were approximately 25%. With pregabalin side effects were surprisingly *not dose-dependent*.

A systematic review of **gabapentin** reported an average dosing regimen of **1200mg per day**. The number needed to treat (NNT) in order to provide a **50% pain reduction** was 5.9 persons for DPN and 8.0 for PHN. 11% of patients discontinued treatment due to side effects. Dizziness was the most common side effect occurring in 19% of patients with somnolence reported in 14% of patients. Additional side effects were reported at much lower rates in this review included peripheral edema and gait disturbance. Both systematic reviews reported rates of serious adverse events that were no more common than placebo.

Similar findings have been reported in surgical patients in a wide variety of surgeries (e.g. abdominal, orthopedic). The dose-response relationship is also very clear in these populations with substantial benefit starting with single preoperative doses of ≥600 mg PO or with daily dosing regimens as noted above. Of note, approximately 1% of patients taking gabapentin will develop significant hyponatremia (<130mEq/L). This typically resolves within 24 hours of stopping therapy.

In summary, systematic reviews of **gabapentinoid use in medical and surgical patients support substantial analgesic benefit (30-50% reduction in pain scores) at higher dosing regimens with relatively common minor side**

© 2022 McEvoy MD et al. *JAMA Network Open.*

effects (e.g. somnolence), but no serious adverse events (e.g. respiratory depression) when compared to placebo.

References:

1. Moore RA, et al. *Cochrane Database Syst Rev* 2014; 27(4).
2. Moore RA, et al. *Cochrane Database Syst Rev* 2009; 8(3).
3. Tiippana EM, et al. *Anesth & Analgesia* 2007; 104:1545-56.

© 2022 McEvoy MD et al. *JAMA Network Open.*

Question 33:
Question: An opioid-naïve patient takes one tab of hydrocodone 5 mg / acetaminophen 325 mg three times a day for five days for low back and radicular pain. Following resolution of the pain, what is the safest way for the patient to transition off of opioids?

Answer

A. Take half a hydrocodone tablet three times a day for three days, then discontinue
B. Taper frequency to 5 mg hydrocodone once a day for three days, then discontinue
C. Stop hydrocodone, take clonidine 0.1 mg PO twice a day for withdrawal symptoms
D. Stop hydrocodone, no tapering necessary

Key point:
After low-dose, short-duration therapy, opioids may be stopped without tapering. Additionally, per latest evidence, prescriptions should be as short as possible to reduce the likelihood of chronic opioid use and to reduce the risk of excess pills available for diversion in the community.

Rationale:
Per the latest CDC guidelines, 3 days of opioid therapy is often sufficient and >7 days is rarely needed for acute pain. If these recommendations are followed, opioid withdrawal is unlikely. Stopping opioids without tapering is acceptable under these circumstances. The incidence of withdrawal increases with longer durations and higher doses of opioids. Unlike benzodiazepine or alcohol withdrawal, opioid withdrawal is not life-threatening in patients with few comorbidities.

In attempting to achieve optimal analgesia for opioid-naïve patients in acute pain, the guiding principles include: **1) Opioids should be "added last, prescribed only as needed (PRN), on for the shortest period of time possible, and taken off of the analgesia regimen first; 2) Non-opioid analgesic medications should be "added first, ordered as scheduled medications, on longer than any other analgesic, and thus taken off of the regimen last."** We call this the **"Function First, Opioids Last" Program**.

For this patient, that would include scheduling acetaminophen and ibuprofen (or other NSAID) while using opioids only as needed and giving as few pills as possible. This patient likely would have had adequate analgesia if the same prescription was given with only 10 pills total.

References:
https://www.tn.gov/assets/entities/health/attachments/ChronicPainGuidelines.pdf
https://www.cdc.gov/mmwr/volumes/66/wr/mm6610a1.htm?s_cid=mm6610a1_e

Question 34: Risk of chronic use [McEvoy/Edwards]
Question: A 54-year-old opioid naïve man presents for colectomy. He is

© 2022 McEvoy MD et al. *JAMA Network Open.*

discharged home with a prescription for Percocet (oxycodone/acetaminophen). Which of the following is his approximate risk of chronic opioid use >3 months after surgery?

Answers:

A. 1%
B. 5%
C. 10%
D. 15%

Key Point: A recent *JAMA Surgery* paper highlighted that approximately 10% of opioid-naïve patients will become chronic opioid users after colectomy (defined as use >3 months from surgery).

Rationale: Based on the CDC Guidelines for Prescribing Opioids for Chronic Pain, literature supporting long-term opioid therapy for pain is limited; in fact, research suggests an increased risk for harms with long-term opioid use in surgical and medical patients.

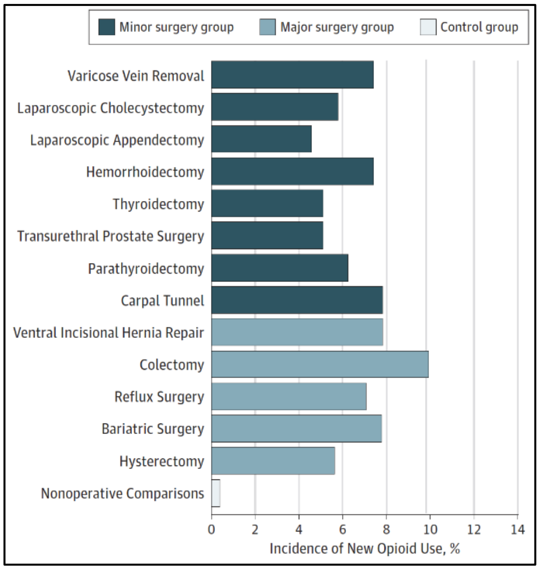

A recent report from Brummett et al. notes that approximately 5-7% of opioid-naïve patients undergoing major or minor surgery will become persistent opioid users (continued use >3 months from surgery). Of note, this rate was 10% for patients undergoing colectomy (see Figure).

Non-surgical patients are at even greater risk. Early opioid prescribing patterns for opioid-naïve medical patients have been found to be associated with the likelihood of long-term use. A recent report noted that in a representative sample of ~1.3 million opioid naïve, cancer-free adults who received at least a 1-day prescription for opioid pain relievers, the likelihood of chronic opioid use increased with each additional day of medication supplied starting with the third day, and risk of use at 1-year doubled with a 5-day prescription. The ***sharpest increases in chronic opioid use*** observed: 1) after the 5th and 31st day on therapy, 2) if a patient received a second opioid prescription, 3) an initial 10- or 30-day supply, or 4) if the prescription was over 700 morphine milligram equivalents (MME) cumulative dose (e.g. oxycodone 10mg PO q6h PRN pain x 14 days = 840 MME). The highest probability of continued opioid use at 1 and 3 years was observed among patients who were started on a long-acting opioid.

In summary, for cancer-free, previously opioid-naïve medical patients receiving a 10-day supply of opioids, approximately 20% are still using opioids after 1 year, and for patients receiving a 30-day supply as their initial opioid prescription, approximately 35% are still using opioids after 1 year. These rates are

© 2022 McEvoy MD et al. *JAMA Network Open.*

approximately 6-7% for all surgical patients.

Thus, care should be taken to a) maximize use of scheduled non-opioid analgesics, b) use opioids for the shortest duration possible, including the lowest dose, shortest duration, and fewest pills prescribed.

References:
https://www.cdc.gov/mmwr/volumes/66/wr/mm6610a1.htm?s_cid=mm6610a1_e
Brummett CM, et al. JAMA Surg. 2017;[Epub ahead of print]

© 2022 McEvoy MD et al. *JAMA Network Open.*

Question 35: Avoid Long-acting – [ALLEN/RICE]
Question: A 56-year-old female with chronic abdominal pain who takes immediate release morphine is admitted with complaints of poorly controlled pain. Which of the following best represents the risk of death in the subsequent 30 days if controlled release morphine is initiated as compared to starting gabapentin for this patient?

Answers:

A. No change in risk
B. 50% reduction in risk
C. Doubling of risk
D. Quadrupling of risk

Key point: Initiation of long acting opioids results in higher mortality from out-of-hospital death, cardiovascular death, unintentional overdose, and other causes of death. **The hazard ratio (HR) during the first 30 days of therapy with a long-acting opioid compared to non-opioid medications was 4.16 (95% CI, 2.27-7.63).**

Rationale: Long-acting opioids increase the risk of unintentional overdose deaths but also may increase mortality from cardiorespiratory and other causes. There is no evidence that long-acting opioids result in improved pain control, but evidence does exist for use of multimodal analgesics to improve pain control in non-cancer pain.

A recent study was performed in Tennessee comparing all-cause mortality for patients with chronic non-cancer pain (non-palliative nor end-of-life care), who were prescribed either long-acting opioids or alternative analgesics for moderate to severe chronic pain. Propensity score-matching was used to compare new episodes of prescriptions for long-acting opioids versus either analgesic anticonvulsants (e.g. gabapentinoids) or low-dose cyclic antidepressants (control medications). Total and cause-specific mortality were determined from death certificates. Of 22,912 new episodes of prescribed therapy for both long-acting opioids and control medications, the HR for total mortality was 1.64 (95% CI, 1.26-2.12) for those prescribed long-acting opioids. Increased risk was due to out-of-hospital deaths (HR, 1.90; 95% CI, 1.40-2.58). For out-of-hospital deaths other than unintentional overdose, the HR was 1.72 (95% CI, 1.24-2.39). **The HR for cardiovascular deaths was 1.65 (95% CI, 1.10-2.46). The HR during the first 30 days of therapy was 4.16 (95% CI, 2.27-7.63).**

This recent data, taken in context of all that is known about chronic opioid use, would suggest that maximizing non-opioid analgesics (e.g. acetaminophen, NSAIDs, gabapentinoids, and antidepressants) is preferable to starting long-acting opioids for non-cancer, non-palliative pain management. As this form of pain management can be complex and nuanced, it is appropriate to seek a consult from a pain management expert. In summary, prescription of long-acting

© 2022 McEvoy MD et al. *JAMA Network Open.*

opioids for chronic non-cancer pain compared to anticonvulsants or cyclic antidepressants was associated with a significantly increased risk of all-cause mortality, including deaths from causes other than overdose.

Reference:
Ray WA, et al. JAMA. 2016;315(22):2415-2419.

Question 36: opioid schedules II vs. IV
Question: Which of the following statements is correct concerning prescriptions for Schedule I-V medications in routine situations?
Answers:

A. Schedule I narcotic prescriptions must be written/printed to be filled
B. Schedule II narcotic prescriptions can be phoned in during an emergency as long as a written prescription is taken to the pharmacy within 14 days.
C. Schedule III narcotic prescriptions can be refilled up to 5 times in 6 months
D. Schedule IV narcotic prescriptions must be written/printed to be filled

Key Point: Prescriptions for **schedules III through V** controlled substances may by **written, or transmitted by fax.** Prescriptions for **schedules III and IV** controlled substances may be **refilled up to 5 times in 6 months.**

Rationale: Drugs and other substances that are considered controlled substances under the Controlled Substances Act (CSA) are divided into five schedules.

Schedule I narcotics have no currently accepted medical use in the United States, a lack of accepted safety for use under medical supervision, and a high potential for abuse (e.g. heroin, LSD).

Schedule II narcotics have a high potential for abuse which may lead to severe psychological or physical dependence (e.g. hydrocodone, hydromorphone (Dilaudid®), oxycodone (OxyContin®, Percocet®)). **Prescriptions for schedule II controlled substances must be written and signed by the practitioner. Prescriptions for schedule II controlled substances cannot be refilled. A new prescription must be issued. In emergency situations, a prescription for a schedule II controlled substance may be telephoned to the pharmacy and the prescriber must follow up with a written prescription being sent to the pharmacy within 7 days.**

Schedule III substances have a potential for abuse less than substances in Schedules I or II and abuse may lead to moderate or low physical dependence or high psychological dependence (e.g. products containing not more than 90 milligrams of codeine per dosage unit (Tylenol with Codeine®), and buprenorphine (Suboxone®).

Schedule IV substances have a low potential for abuse relative to substances in Schedule III (e.g. tramadol (Ultram), alprazolam (Xanax®), carisoprodol (Soma®), clonazepam (Klonopin®)). However, the combination of Schedule II and IV medications puts a patient at greater risk of drug-related overdose.

Schedule V substances have low risk of abuse and prescriptions for **schedule V** controlled substances may be **refilled as authorized by the practitioner.**

Prescriptions for **schedules III through V** controlled substances may by **written, or transmitted by fax.** Prescriptions for **schedules III and IV** controlled substances may be **refilled up to 5 times in 6 months.** All prescriptions for schedule II-IV medications

© 2022 McEvoy MD et al. *JAMA Network Open.*

should include the **dose, frequency, and number of pills to be filled,** as well as the **maximum number of pills to be taken in a 24 hour period**.

For treating acute conditions, the fewest number of pills expected to be needed should be prescribed in order to reduce potential for abuse, dependence, or diversion. **One strategy would be to prescribe tramadol for short courses of therapy (Schedule IV) rather than oxycodone (Schedule II), as a refill of tramadol could be given by fax or phone without necessitating a return to clinic or hospital.**

References: **https://www.deadiversion.usdoj.gov/schedules/index.html**

© 2022 McEvoy MD et al. *JAMA Network Open.*

Question 37: Tramadol – benefits [MCEVOY/EDWARDS]

Question: Which of the following is TRUE concerning pain relief with tramadol 100 mg PO combined with an NSAID (non-steroidal anti-inflammatory drug) for immediate postoperative pain control?

Answers:

A. No difference between tramadol+NSAID and placebo
B. No difference between tramadol+NSAID versus tramadol alone, but better than placebo
C. No difference between tramadol+NSAID versus NSAID alone, but better than placebo
D. Better pain control with use of tramadol+NSAID versus placebo or either alone

Key Point: A recent Cochrane review concerning treatment of acute postoperative pain concluded that a single oral dose of dexketoprofen 25 mg (an NSAID) plus tramadol 75 mg provided good levels of pain relief with long duration of action to more people than placebo or the same dose of dexketoprofen or tramadol alone. The magnitude of the effect was similar to other good analgesics. Adverse event rates were low.

Rationale: In the setting of musculoskeletal pain, the atypical, centrally acting analgesic tramadol (Ultram) offers important benefits. Its multi-modal effect results from a dual mode of action, i.e., opioid and monoaminergic mechanisms, with efficacy in both nociceptive and neuropathic pain. Moreover, fewer instances of side effects such as constipation, respiratory depression, and sedation occur than with traditional opioids. Tramadol is now regarded as a first-line analgesic for many musculoskeletal and post-surgical indications.

Accordingly, a recent Cochrane review concerning treatment of acute postoperative pain concluded that a single oral dose of dexketoprofen 25 mg (an NSAID) plus tramadol 75 mg provided good levels of pain relief with long duration of action to more people than placebo or the same dose of dexketoprofen or tramadol alone. The magnitude of the effect was similar to other good analgesics. Adverse event rates were low. Dexketoprofen 25mg PO is approximately equal to ibuprofen 600 mg PO.

Additional benefits of using tramadol for acute postoperative or non-surgical pain in addition to scheduled non-narcotic multimodal analgesics is that it is a Schedule IV medication, which allows for refills to be authorized by phone or fax. Thus, a prescription for a very short course of tramadol could be given at discharge (e.g. 50mg q6h PO x 2-3 days, total pills = 12) and then refilled over the phone without the patient having to return for a written prescription, if needed. This would allow for safer narcotic prescribing for the patient and the community.

© 2022 McEvoy MD et al. *JAMA Network Open.*

However, it should be noted that there is a risk of serotonin syndrome with tramadol use for patients who are taking SSRIs, SNRIs, and MAOIs. Even with the benefits listed above, tramadol use should be avoided in patients taking these medications.

References:

Qaseem A, et al. Ann Intern Med. 2017 (https://www-ncbi-nlm-nih-gov.proxy.library.vanderbilt.edu/pubmed/28192789)

Derry S, et al. Cochrane Database Syst Rev. 2016:CD012232. (https://www-ncbi-nlm-nih-gov.proxy.library.vanderbilt.edu/pubmed/27654994)

© 2022 McEvoy MD et al. *JAMA Network Open.*

Question 38: Tramadol contraindications [MCEVOY/EDWARDS]

Question: Which of the following medications is NOT safe to use in a pain regimen along with tramadol?

Answers:

A. Celecoxib (Celebrex)
B. Duloxetine (Cymbalta)
C. Pregabalin (Lyrica)
D. Acetaminophen (Tylenol)

Key Point: Tramadol use with SSRIs, SNRIs, and MAOIs should be undertaken with caution, if at all.

Rationale: Since chronic pain syndromes and depression simultaneously occur in many patients, it is likely that tramadol and an SSRI, SNRI, or MAOI could be used together. Concurrent use of these drugs is known to increase the **risk for seizures and serotonin syndrome**. Tramadol is a mu-opioid receptor agonist as well as an inhibitor of the reuptake of norepinephrine and serotonin in the central nervous system (CNS). SSRIs contribute to this interaction by increasing serotonin levels in the CNS and may also inhibit the metabolism of tramadol via CYP2D6, thereby increasing the concentration of tramadol. The coadministration of tramadol and SSRI antidepressants should be prescribed with caution, if at all. This interaction is also possible with SNRIs and MAOIs.

Additionally, as with any analgesic medication that can cause sedation, such as tramadol or other opioids, patient risk should be assessed for combinations causing sedation, such as combining gabapentinoids or alpha-2 agonists with opioids.

References:

Beakley BD. Tramadol, Pharmacology, Side Effects, and Serotonin Syndrome: A Review. Pain Physician 2015; 18:395-400 (http://www.painphysicianjournal.com/current/pdf?article=MjM3OA%3D%3D&journal=89)

http://www.ebmconsult.com/articles/tramadol-interaction-ssri-serotonin-syndrome-mechanism

© 2022 McEvoy MD et al. *JAMA Network Open.*

Question 39: OPIOIDS: RISK WITH OTHER SEDATIVES [MCEVOY/EDWARDS]

Question: A 32 yo female is being discharged home after resolution of a flare of her Crohn's disease. She has taken hydrocodone and alprazolam for over two years for low back pain and muscle spasms. Which of the following statements is correct regarding the combination of hydrocodone and alprazolam as you prepare her post-discharge analgesic regimen?

Answers:

A. This combination is an effective treatment for long-term musculoskeletal pain and increasing the hydrocodone dose temporarily for acute on chronic pain does not increase patient risk
B. As this combination has been used for long-term treatment of pain in this patient, there is no benefit of adding non-opioid multimodal analgesics for acute on chronic pain exacerbations
C. This combination increases the risk for serious adverse effects such as respiratory depression and death and thus any increase of opioid dosing for acute or chronic pain should be undertaken with caution
D. As this combination is not an effective treatment for musculoskeletal pain and poses risk to the patient, these should be discontinued and a different analgesic combination should be used.

Key Point: Combinations of opioids and benzodiazepines places patients at greater risk of serious adverse events and death than the use of either alone. In a period of acute pain, increasing doses of these medications, especially upon discharge, can place the patient at increased risk of adverse events.

Rationale: In 2016, the FDA required changes to drug labeling to require box warnings to health professionals and patients regarding the serious risks associated with combined use of opioids and benzodiazepines. Neither opioids or benzodiazepines have been shown to be an effective treatment for chronic low back pain and their use, especially in combination, is associated with serious risk for respiratory depression and death. **Utilizing these medications for physiologic "legitimate" pain does not reduce the risk for physiologic dependence, addiction, or serious adverse events. In fact, combinations of opioids and benzodiazepines places patients at greater risk of serious adverse events and death than the use of either alone. Based upon these risks, the most recent guidelines from the American Society of Interventional Pain Physicians recommends to "not combine opioids with sedative hypnotics, benzodiazepines, or barbiturates for chronic, non-cancer pain unless there is a specific medical indication for the combination."**

Increasing doses of these medications, especially upon discharge to a non-monitored setting, can place the patient at increased risk of adverse events. Although continuing this medication combination would not be "absolutely

© 2022 McEvoy MD et al. *JAMA Network Open.*

contraindicated," in this scenario the physician should encourage a multidisciplinary treatment plan to address the acute postoperative pain in the setting of chronic low back pain. The long-term plan should explore addiction risk factors and transition away from potentially harmful and addictive medications. **The short-term plan should take into account the increased risk of adverse events with these combinations and consider: 1) appropriate scheduled use of non-sedating non-opioid analgesics, 2) coordinating with the healthcare professional who is providing the chronic narcotics to the patient, 3) patient education concerning the goal of optimal analgesia, which takes into account functional recovery and side effects in addition to pain ratings, and 4) consideration of consultation with a chronic pain medicine expert.**

References

Jones J, et al. *Drug and Alcohol Dependence,* 2012;125:8-18
Manchikanti L, et al. *Pain Physician,* 2017;20:S3-S92.
Scott MJ, et al. *Perioperative Med*, 2017

© 2022 McEvoy MD et al. *JAMA Network Open.*

Question 40: OPIOIDS: CHRONIC PAIN/OPIOID USE [McEVOY/EDWARDS]
Question: A 38 yo female is admitted to your service. Review of her medical record reveals that she has used extended-release morphine for several years to manage chronic low back and pelvic pain. Based on this information alone, what is the approximate likelihood that this patient has a recent history of opioid MISUSE?

Answers:
A. 5%
B. 15%
C. 25%
D. 35%

Key Point: Opioid **misuse** implies a use that is contrary to what is recommended, such as taking a medication more often than prescribed. Of chronic pain patients, about 1 in 4 (21-29%) have a recent history of opioid misuse.

Rationale:
Current research suggests that about 1 in 10 (8-12%) chronic pain patients manifest behavior consistent with opioid addiction. Of chronic pain patients, about 1 in 4 (21-29%) have a recent history of opioid misuse. Medical terminology in the field of addiction medicine is rapidly evolving and sometimes confusing.

Opioid **misuse** implies a use that is contrary to what is recommended, such as taking a medication more often than prescribed.

Opioid **abuse** is the intentional use of an opioid for nonmedical purposes, for the euphoric effects or to "get high."

Opioid **addiction** is a primary, chronic and relapsing brain disease characterized by an individual pathologically pursuing reward and/or relief by substance use. The term addiction implies a pattern of maladaptive behavior characterized by ongoing use despite harm or potential for harm, and this is present in about 10% of chronic opioid users. There is a significant negative stigma attached to the word addiction, so in an effort to destigmatize patients, the more appropriate term to use is "substance use disorder," or more specifically "opioid use disorder." The DSM-5 utilizes terminology focused upon "substance use disorders", rather than addiction, and divides these into categories based upon severity: mild, moderate, and severe.

Physical dependence upon an opioid is a physiological consequence of chronic use and is not necessarily indicative of behavioral patterns of misuse, abuse, or addiction.

© 2022 McEvoy MD et al. *JAMA Network Open.*

References

Vowles et al. Pain, 2015;156:4
Webster L, et al. Avoiding Opioid Abuse While Managing Pain; 2007.

© 2022 McEvoy MD et al. *JAMA Network Open.*

List of Vanderbilt Learning Healthcare System Platform Investigators

Vanderbilt University Medical Center and Vanderbilt University, Nashville, TN – Gordon Bernard*, Robert Dittus, Shon Dwyer, Robert Freundlich, Cheryl Gatto, Frank Harrell, Paul Harris, Tina Hartert, Jim Hayman, Catherine Ivory, Kevin Johnson, Ruth Kleinpell, Sunil Kripalani, Christopher Lindsell*, Lee Ann Liska, Patrick Luther, Jay Morrison, Thomas Nantais, Mariann Piano, Jill Pulley, Kris Rehm, Todd Rice*, Russell Rothman, Matt Semler, Robin Steaban, Philip Walker, Consuelo Wilkins, Adam Wright, and Autumn Zuckerman

* Denotes members of the QuizTime Trial Study Team

List of Vanderbilt Committee on Opioid Monitoring and Stewardship

Tyler Barrett*, Michael Costello, David Edwards*, R. Scott Frankenfield, Alexander Hawkins, Sterling Haring, Jason Hessler, Elizabeth Huggins, Carin McAbee, Puneet Mishra, Hayley Rector, Jennifer Slayton, and Irving Zamora

* Denotes members of the QuizTime Trial Study Team

© 2022 McEvoy MD et al. *JAMA Network Open.*

eFigure 1: Example of QuizTime User Interface

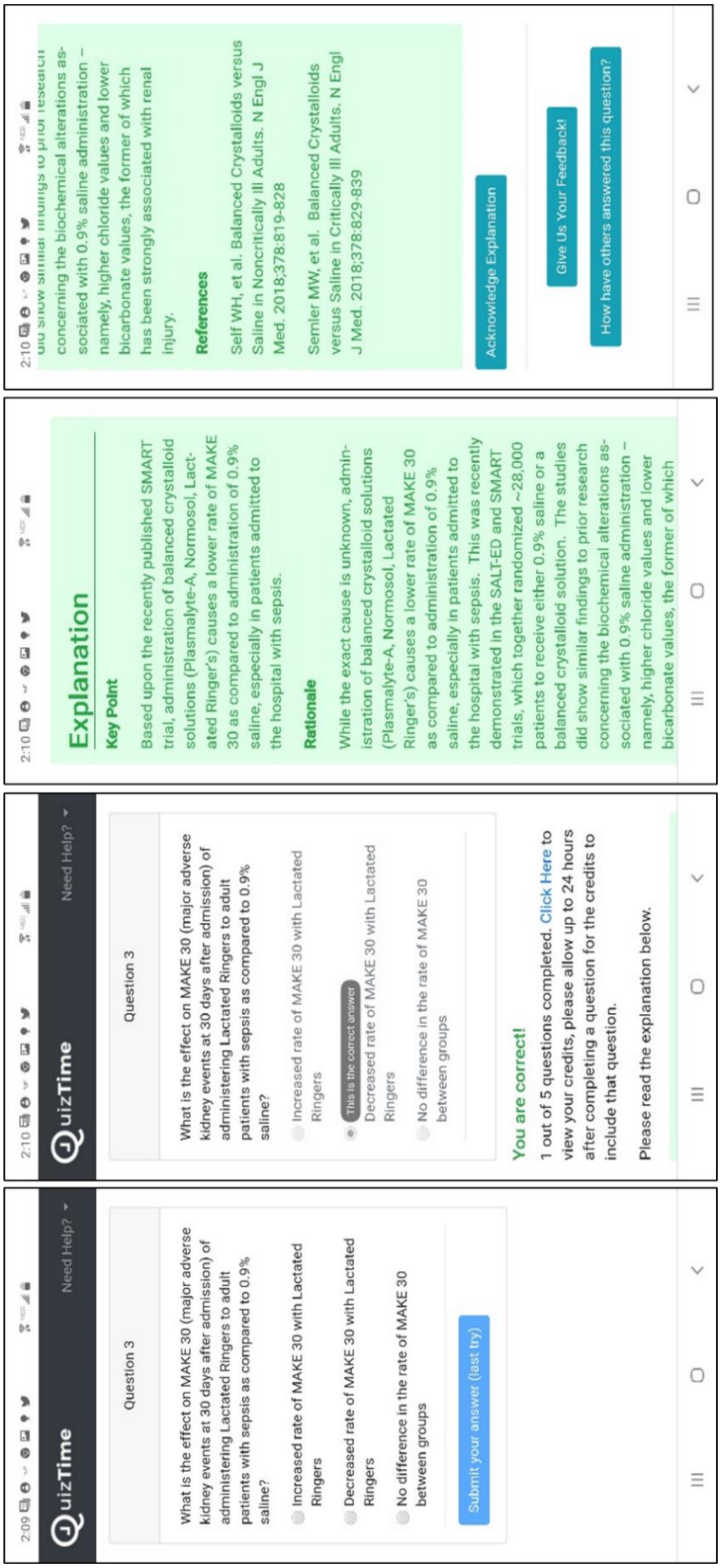

© 2022 McEvoy MD et al. *JAMA Network Open.*

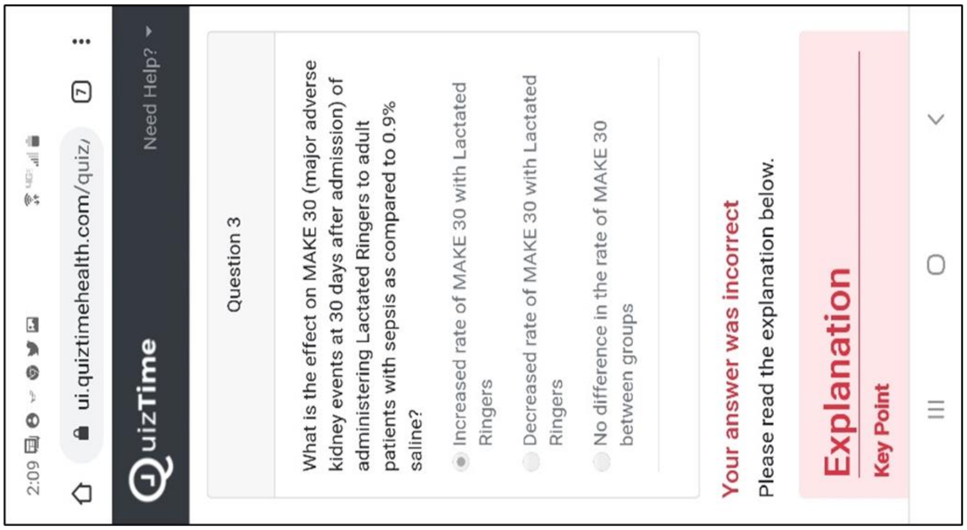

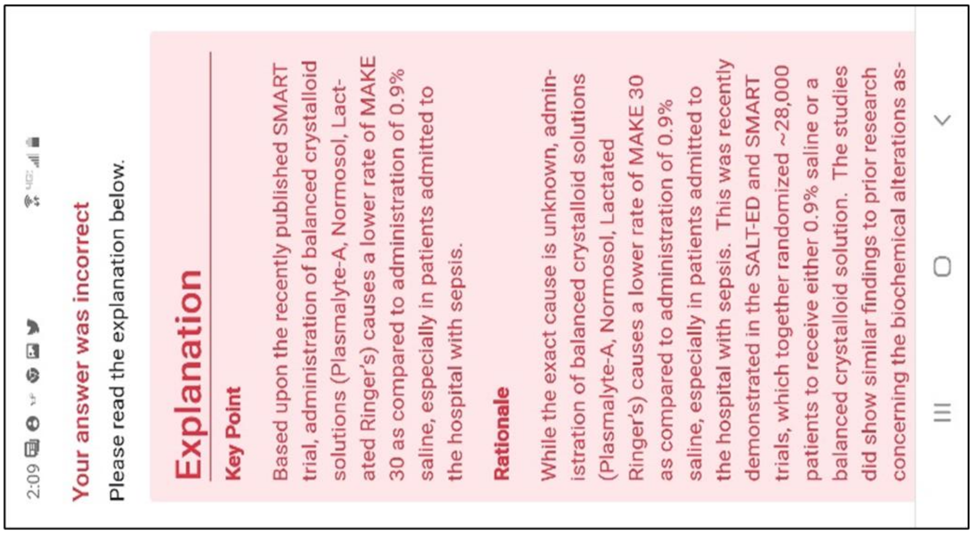

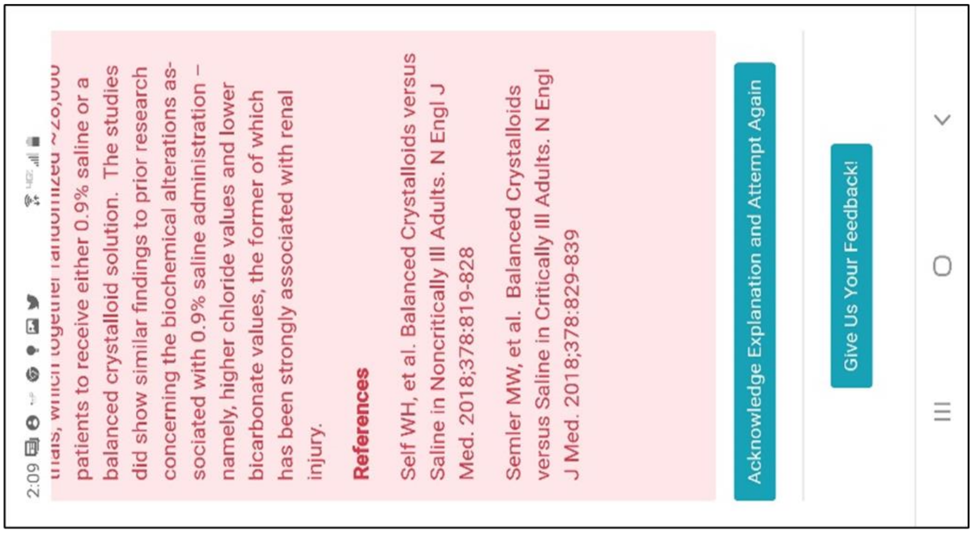

© 2022 McEvoy MD et al. *JAMA Network Open.*

eTable 1: Learner Analytics

		Group 1	Group 2
		N (%)	N (%)
Intravenous Fluids	Delivered	4250 (100)	4092 (100)
	Opened	3078 (72.4)	2520 (61.6)
	Answered (of opened)	2804 (91.1)	2337 (92.7)
	Correct of answered	1830 (65.3)	1525 (65.3)
	Correct of opened	1830 (59.5)	1525 (60.5)
	Second attempt sent	753 (17.7)	632 (15.4)
	Second attempt opened	742 (98.5)	632 (100)
	Second attempt answered (of opened)	740 (99.7)	628 (99.4)
	Correct of second attempt answered	665 (89.9)	562 (89.5)
	Correct of second attempt opened	665 (89.6)	562 (88.9)
Function First, Opioids Last	Delivered	4329 (100)	4197 (100)
	Opened	3384 (78.2)	2801 (66.7)
	Answered (of opened)	3130 (92.5)	2614 (93.3)
	Correct of answered	2083 (66.5)	1737 (66.6)
	Correct of opened	2083 (62.0)	1737 (61.6)
	Second attempt sent	846 (19.5)	697 (16.6)
	Second attempt opened	832 (98.3)	697 (100)
	Second attempt answered (of opened)	826 (99.3)	695 (99.7)
	Correct of second attempt answered	716 (89.4)	621 (86.7)
	Correct of second attempt opened	716 (89.1)	621 (86.1)

The colors indicate the order in which each group completed the learning modules with green indicating the learning module/quiz that was completed first and gold indicating the learning module/quiz that was completed second (i.e. Group 1 completed the intravenous fluids module first and the opioid/pain medication module second).

© 2022 McEvoy MD et al. *JAMA Network Open.*
