# Supplementary material for: Effect of Smartphone App–Based Education on Clinician Prescribing Habits in a Learning Health Care System: A Randomized Cluster Crossover Trial
Source: JAMA Netw Open. 2022 Jul 26;5(7):e2223099. doi: 10.1001/jamanetworkopen.2022.23099 (PMC9327570; doi:10.1001/jamanetworkopen.2022.23099)
Supplement: Supplement 3. — Nonauthor Collaborators. The Vanderbilt Learning Healthcare System Platform Investigators and the Vanderbilt Committee on Opioid Monitoring and Stewardship [file jamanetwopen-e2223099-s003.pdf]

Supplemental Online Content: Nonauthor Collaborators

*Indicates required information. Only first name, last name, and suffix will appear in PubMed.

*Group Name(s): The Vanderbilt Learning Healthcare System Platform Investigators and the Vanderbilt Committee on Opioid Monitoring and Stewardship							
***First Name and Middle Initial(s)**	***Last Name**	***Suffix (eg, Jr, III)**	Academic Degrees	Institution	Location (city, state/province, country)	Role or Contribution, eg, chair, principal investigator	Group (if more than 1 Group listed in the byline) and/or Subgroup (eg, Steering Committee)
Robert	Dittus						Vanderbilt Learning Healthcare System Platform Investigators
Shon	Dwyer						Vanderbilt Learning Healthcare System Platform Investigators
Robert	Freundlich						Vanderbilt Learning Healthcare System Platform Investigators
Cheryl	Gatto						Vanderbilt Learning Healthcare System Platform Investigators
Frank	Harrell						Vanderbilt Learning Healthcare System Platform Investigators
Paul	Harris						Vanderbilt Learning Healthcare System Platform Investigators
Tina	Hartert						Vanderbilt Learning Healthcare System Platform Investigators
Jim	Hayman						Vanderbilt Learning Healthcare System Platform Investigators
Catherine	Ivory						Vanderbilt Learning Healthcare System Platform Investigators

Supplemental Online Content: Nonauthor Collaborators

*Indicates required information. Only first name, last name, and suffix will appear in PubMed.

***First Name and Middle Initial(s)**	***Last Name**	***Suffix (eg, Jr, III)**	Academic Degrees	Institution	Location (city, state/province, country)	Role or Contribution, eg, chair, principal investigator	Group (if more than 1 Group listed in the byline) and/or Subgroup (eg, Steering Committee)
Kevin	Johnson						Vanderbilt Learning Healthcare System Platform Investigators
Ruth	Kleinpell						Vanderbilt Learning Healthcare System Platform Investigators
Sunil	Kripalani						Vanderbilt Learning Healthcare System Platform Investigators
Lee Ann	Liska						Vanderbilt Learning Healthcare System Platform Investigators
Patrick	Luther						Vanderbilt Learning Healthcare System Platform Investigators
Jay	Morrison						Vanderbilt Learning Healthcare System Platform Investigators
Thomas	Nantais						Vanderbilt Learning Healthcare System Platform Investigators
Mariann	Piano						Vanderbilt Learning Healthcare System Platform Investigators
Jill	Pulley						Vanderbilt Learning Healthcare System Platform Investigators
Kris	Rehm						Vanderbilt Learning Healthcare System Platform Investigators

Supplemental Online Content: Nonauthor Collaborators

*Indicates required information. Only first name, last name, and suffix will appear in PubMed.

***First Name and Middle Initial(s)**	***Last Name**	***Suffix (eg, Jr, III)**	Academic Degrees	Institution	Location (city, state/province, country)	Role or Contribution, eg, chair, principal investigator	Group (if more than 1 Group listed in the byline) and/or Subgroup (eg, Steering Committee)
Russell	Rothman						Vanderbilt Learning Healthcare System Platform Investigators
Matthew	Semler						Vanderbilt Learning Healthcare System Platform Investigators
Robin	Steaban						Vanderbilt Learning Healthcare System Platform Investigators
Consuelo	Wilkins						Vanderbilt Learning Healthcare System Platform Investigators
Adam	Wright						Vanderbilt Learning Healthcare System Platform Investigators
Autumn	Zuckerman						Vanderbilt Learning Healthcare System Platform Investigators
Michael	Costello						Vanderbilt Committee on Opioid Monitoring and Stewardship
R. Scott	Frankenfield						Vanderbilt Committee on Opioid Monitoring and Stewardship
Alexander	Hawkins						Vanderbilt Committee on Opioid Monitoring and Stewardship
Sterling	Haring						Vanderbilt Committee on Opioid Monitoring and Stewardship

*Indicates required information. Only first name, last name, and suffix will appear in PubMed.

***First Name and Middle Initial(s)**	***Last Name**	***Suffix (eg, Jr, III)**	Academic Degrees	Institution	Location (city, state/province, country)	Role or Contribution, eg, chair, principal investigator	Group (if more than 1 Group listed in the byline) and/or Subgroup (eg, Steering Committee)
Jason	Hessler						Vanderbilt Committee on Opioid Monitoring and Stewardship
Elizabeth	Huggins						Vanderbilt Committee on Opioid Monitoring and Stewardship
Carin	McAbee						Vanderbilt Committee on Opioid Monitoring and Stewardship
Puneet	Mishra						Vanderbilt Committee on Opioid Monitoring and Stewardship
Hayley	Rector						Vanderbilt Committee on Opioid Monitoring and Stewardship
Jennifer	Slayton						Vanderbilt Committee on Opioid Monitoring and Stewardship
Irving	Zamora						Vanderbilt Committee on Opioid Monitoring and Stewardship
