# Supplementary material for: Effect of Smartphone App–Based Education on Clinician Prescribing Habits in a Learning Health Care System: A Randomized Cluster Crossover Trial
Source: JAMA Netw Open. 2022 Jul 26;5(7):e2223099. doi: 10.1001/jamanetworkopen.2022.23099 (PMC9327570; doi:10.1001/jamanetworkopen.2022.23099)
Supplement: Supplement 4. — Data Sharing Statement [file jamanetwopen-e2223099-s004.pdf]

Data Sharing Statement

McEvoy. Effect of Smartphone App-Based Education on Clinician Prescribing Habits in a Learning Health Care System. *JAMA Netw Open*. Published July 26, 2022. doi:10.1001/jamanetworkopen.2022.23099

Data
Data available: Yes
Data types: Deidentified participant data, Data dictionary
How to access data: This trial is a prospective RCT concerning an educational intervention with clinicians, not patients. However, data can be made available of individual participants.
When available: With publication

Supporting Documents
Document types: Statistical/analytic code, Informed consent form
How to access documents: We will make them available through a website hosted by Vanderbilt University or can be posted on a website hosted by JAMA Network.
When available: With publication

Additional Information
Who can access the data: researchers whose proposed use of the data has been approved
Types of analyses: for analysis of results for approved research purposes (e.g. meta-analysis or systematic reviews)
Mechanisms of data availability: after approval and with a signed data agreement
